# Goondoxazoles A–C: Anthelmintic Spiroketal Polyketide Alkaloids and Other Benzoxazoles from Australian Pasture Soil-Derived *Streptomyces* spp.

**DOI:** 10.3390/antibiotics15030302

**Published:** 2026-03-17

**Authors:** Shengbin Jin, David F. Bruhn, Erica J. Burkman, Cynthia T. Childs, Jianying Han, Zeinab G. Khalil, Yovany Moreno, Angela A. Salim, Kaumadi Samarasekera, Marcelo M. P. Tangerina, Robert J. Capon

**Affiliations:** 1 Institute for Molecular Bioscience, The University of Queensland, St Lucia, QLD 4072, Australiakaumadi@sjp.ac.lk (K.S.); marcelomptang@gmail.com (M.M.P.T.); 2 Boehringer Ingelheim Animal Health, USA Inc., 1730 Olympic Drive, Athens, GA 30601, USA; david.bruhn@boehringer-ingelheim.com (D.F.B.); erica.burkman@boehringer-ingelheim.com (E.J.B.);; 3 Botany Department, Institute of Biosciences, University of São Paulo-USP, São Paulo 05508-090, SP, Brazil

**Keywords:** anthelmintic, citizen science, *Dirofilaria immitis*, *Haemonchus contortus*, microbial natural product, polyketide, spiroketal, alkaloid, benzoxazole, Soils for Science, *Streptomyces*

## Abstract

**Background/Objectives/Methods:** A bioassay-informed investigation of the Australian pasture soil-derived *Streptomyces* sp. S4S-00193A39 yielded the anthelmintic principals as three new spiroketal polyketide alkaloids, goondoxazoles A–C (**1**–**3**), with structures assigned by detailed spectroscopic analysis. **Results:** A structure–activity relationship based on the ability to inhibit the motility of *Dirofilaria immitis* microfilariae (mf) revealed a positive correlation for the benzoxazole moiety present in **2** and **3** (EC_50_ 55–85 nM) versus the ring-opened aminobenzoic acid moiety evident in **1** (EC_50_ 1.38 µM). This hypothesis was strengthened by extension of the SAR assessment to the known benzoxazole natural products A-33583 (**12**), UK-1 (**13**) and nataxazole (**14**), and the new analogue 5-hydroxynataxazole (**15**), which were isolated in our lab from three additional Australian pasture soil-derived *Streptomyces* spp. Of note, while the benzoxazole methyl esters **13**–**15** exhibited approximately 9- to 65-fold lower potency against *D. immitis* mf compared with **2** and **3**, the carboxylic acid substituted benzoxazole **12** displayed comparable activity (EC_50_ 72 nM) against *D. immitis* mf, and >5-fold improved potency against *D. immitis* L4 larvae (EC_50_ 0.43 µM). **Conclusions:** These observations reveal the promising anthelmintic potential (against *D. immitis*) for the new structurally complex and chiral goondoxazoles (e.g., **2** and **3**), and demonstrate that this effect can be replicated, even improved, by simpler, achiral benzoxazole microbial natural products (e.g., **12**).

## 1. Introduction

**Figure 1:**
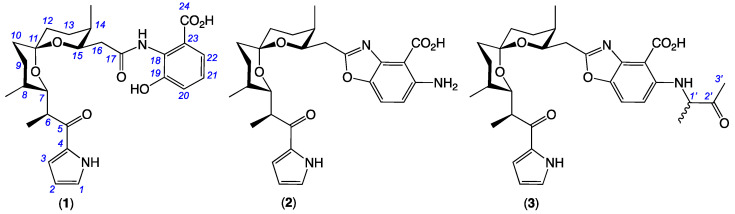
Goondoxazoles A–C (**1**–**3**) isolated from *Streptomyces* sp. S4S-00193A39.

*Dirofilaria immitis*, the causative agent of heartworm disease, represents one of the most significant and potentially fatal veterinary parasitic infections globally, primarily affecting the pulmonary arteries and right hearts of canines and felines [1]. Current control strategies rely heavily on the monthly administration of macrocyclic lactones (e.g., ivermectin, moxidectin, milbemycin oxime and selamectin), which are highly effective at eliminating the larvae before they reach the heart [2,3]. During investigations into bioactive natural products from Australian soil-derived microbes, a library of bacterial and fungal isolates (×1893) was assembled from pasture soils (×103) collected from cattle and sheep stations across Australia. Solvent extracts prepared from ISP2 and M1 agar plate cultivations of pure isolates were assessed for their ability to inhibit the motility of dog heartworm *Dirofilaria immitis* microfilariae (mf), and/or the development of livestock gastrointestinal *Haemonchus contortus* L1–L3 larvae. Active extracts were further prioritized by chemical profiling to differentiate new from known and rare from common natural product classes. This discovery strategy has proved highly productive, as demonstrated in recent reports on the anthelmintic polyketide goondapyrones from *Streptomyces* sp. S4S-00196A10 [4], carbocyclic *ansa*-polyketide goondomycins from *Streptomyces* sp. S4S-00052A05 [5], *spiro*-isoindolinone goondicones from *Streptomyces* sp. S4S-00185A06 [6], and terpenyl-quinolin-4(1H)-one goondolinones from *Actinomadura* sp. S4S-00245B09 [7]. This current report describes a chemical investigation prompted by *Streptomyces* sp. S4S-00193A39, which was prioritized on the basis of the selective inhibition of *D. immitis* mf (100% at 2.5 µg/mL) and the detection of natural products with molecular formula unprecedented in the bacterial natural product scientific literature. Following cultivation profiling, fractionation of an optimized scaled-up cultivation of S4S-00193A39 yielded the anthelmintic principals as goondoxazoles A–C (**1**–**3**) (Figure 1), new examples of a rare class of pyrrolo-spiroketal polyketide, known examples of which are limited to **4**–**11**. Structures were assigned to **1**–**3** on the basis of detailed spectroscopic analysis, biosynthetic considerations and literature comparisons. A structure–activity relationship (SAR) assessment of **1**–**3** suggested a correlation between the benzoxazole moiety and the inhibition of both *D. immitis* mf and L4 larvae. This SAR hypothesis was further validated and expanded to other pasture soil-derived *Streptomyces* spp. that yielded an array of natural products, A-33583 (**12**), UK-1 (**13**), nataxazole (**14**) and 5-hydroxynataxazole (**15**), incorporating a benzoxazole moiety and exhibiting selective activity against *D. immitis* mf and L4 larvae.

## 2. Results and Discussion

### 2.1. Structure Elucidation

A UPLC-DAD (210 nm) chromatogram of the EtOAc extract prepared from an ISP2 agar plate cultivation of S4S-00193A39 revealed several co-metabolites with distinctive UV-vis (DAD) chromophores (Figure S3), with high resolution mass measurements attributing molecular formula (C_27_H_34_N_2_O_7_, C_27_H_33_N_3_O_6_, C_31_H_39_N_3_O_7_) unprecedented in the bacterial natural products scientific literature (SciFinder). To further assess molecular novelty, we used a Global Natural Products Social (GNPS) [8] molecular networking approach to compare with ISP2/M1 agar plate extracts prepared from 108 Goondicum soil-derived microbes independently prioritized as exhibiting anthelmintic activity, as well as an internal library of 1957 soil-derived microbes, revealing S4S-00193A39 as the sole producer of the target chemistry (Figure S4). A miniaturized cultivation profiling approach (MATRIX) [9] determined 333 solid-phase agar as optimal for the production of this chemistry (Figure S5), with fractionation of scaled-up cultivation yielding **1**–**3**.

**Figure 2:**
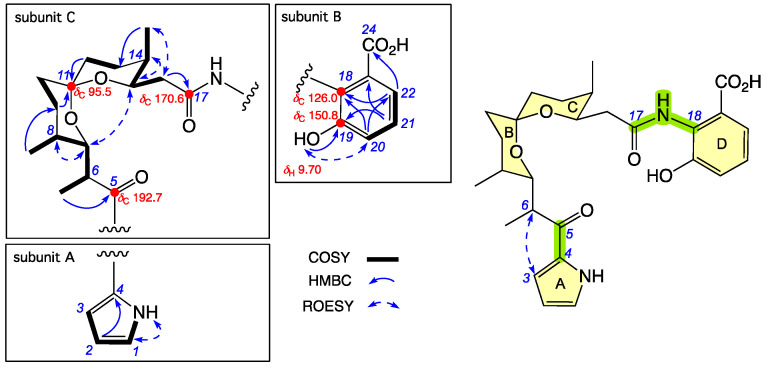
Selected 2D NMR (DMSO-*d*_6_) correlations and chemical shifts for **1**. Highlights: NMR chemical shifts (red); rings A–D (yellow); connections between subunits A–C (green).

HRESI(+)MS measurement established a molecular formula for **1** ([M + H]^+^ Dmmu +2.0, C_27_H_34_N_2_O_7_) requiring 12 double-bond equivalents (DBEs). Analysis of the NMR (DMSO-*d*_6_) data for **1** (Table 1 and Table S2, and Figures S7–S12) revealed resonances for one ketone (*δ*_C_ 192.7), two carbonyl groups (*δ*_C_ 168.5, *δ*_C_ 170.6), and ten sp^2^ carbons, accounting for eight DBEs and requiring that **1** be tetracyclic. Further analysis of the NMR data revealed resonances and correlations attributed to a monosubstituted pyrrole moiety accounting for one ring, with the regiochemistry of substitution evident from a single highly deshielded sp^2^ methine (*δ*_H_ 7.03, H-1; *δ*_C_ 125.2, C-1) and two less deshielded sp^2^ methines (*δ*_H_ 6.14, H-2, *δ*_H_ 6.90, H-3; *δ*_C_ 109.5, C-2, *δ*_C_ 116.4, C-3) (Figure 2, subunit A).

The NMR data also revealed a disubstituted benzoic acid moiety, accounting for a second ring, with the contiguous nature of the three aromatic sp^2^ methines (H-20, H-21 and H-22) evident from their respective *J* coupling and COSY correlations. The presence and regiochemistry of a phenolic moiety was evident from HMBC correlations from a deshielded exchangeable proton (*δ*_H_ 9.70, 19-OH) and an aromatic methine (*δ*_H_ 7.13, dd, H-21), to a quaternary aromatic carbon (*δ*_C_ 126.0, C-18) and a highly deshielded (oxygenated) quaternary aromatic carbon (*δ*_C_ 150.8, C-19); and a ROESY correlation between the same exchangeable proton (19-OH) and an adjacent aromatic methine (*δ*_H_ 7.09, d, H-20). The regiochemistry of the carboxylic acid moiety was evident from an HMBC correlation from the remaining aromatic methine (*δ*_H_ 7.35, H-22) to the carboxylic acid carbonyl (*δ*_C_ 168.5, C-24) (Figure 2, subunit B).

The remaining elements of C_16_H_25_NO_4_ could be assembled into a polyketide chain extending from a ketone (*δ*_C_ 192.7, C-5) through a spiroketal (*δ*_C_ 95.5, C-11) to an amide carbonyl (*δ*_C_ 170.6, C-17), accounting for the remaining two rings. NMR chemical shifts and correlations also established pendant 2°-methyl at C-6, C-8 and C-14, while ROESY correlations between H-7 and H-15, H-7 and H-8, H-14 and H-15, and H_2_-16 and 14-Me, established the relative configuration across all chiral centers in subunit C, with the exception of C-6 (Figure 2, subunit C).

A ROESY correlation between H-3 and H-6 allowed connectivity of subunits A and C, leaving a C-17 to C-18 amide linkage as the only option for connecting subunits A+C to B (Figure 2, green highlights). Interestingly, **1** shares the same chiral subunit C as the biosynthetically and structurally related natural product X-14885A (**4**), first reported in 1983 from *Streptomyces antibioticus* and assigned a structure (relative configuration) on the basis of an X-ray crystallographic analysis [10,11]. The ^1^H NMR (CDCl_3_) chemical shifts reported for the 6-Me (*δ*_H_ 0.96, *δ*_C_ 13.2), 8-Me (*δ*_H_ 0.99, *δ*_C_ 10.6) and 14-Me (*δ*_H_ 0.96, *δ*_C_ 10.9) in **4** closely match those for 6-Me (*δ*_H_ 0.87, *δ*_C_ 13.0), 8-Me (*δ*_H_ 0.96, *δ*_C_ 10.8) and 14-Me (*δ*_H_ 0.88, *δ*_C_ 11.1) in **1** (Table S3, Figures S13 and S14), suggesting a common relative configuration, including C-6. Comparison of the [α]_D_ (CHCl_3_) reported for **4** (+177.0) [11] with that for **1** (+110.9) supports a common absolute configuration, allowing the full structure for goondoxazole A (**1**) to be assigned as shown. Note: As the [α]_D_ reported for **4** in 1983 [11] was acquired from the Na salt, this raised concern about whether the [α]_D_ would be the same (or different) for the free base versus a salt (see prior documentation of salts driving [α]_D_ variability for viridicatumtoxin [12]). To address this concern, [α]_D_ measurements were acquired from an authentic sample of the commercially available ionophore calcimycin (**5**) in different solvents in the absence and presence of Ca^2+^ (Table S13), revealing significant variability and validating our initial concerns. To test the significance of this [α]_D_ variability on **1**, measurements were repeated on CHCl_3_ in the absence and presence of Na^+^ (Table S13). While there were differences, these variations were such that they did not invalidate the view that **1** and **4** share the same absolute configuration. It is also worth noting that while an absolute configuration is presented in the scientific literature for **4** (to be in common with all other known members of this structure class, see below), we could find no definitive report of experimental data to back up this assignment, which appears to rely on biogenetic considerations.

**Figure 3:**
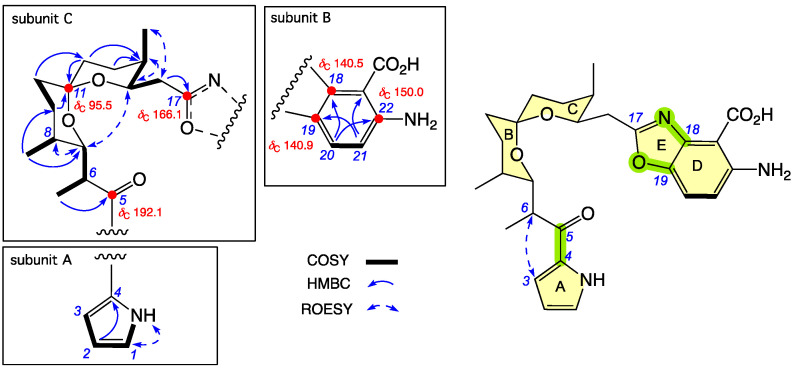
Selected 2D NMR (DMSO-*d*_6_) correlations and chemical shifts for **2**. Highlights: NMR chemical shifts (red); rings A–E (yellow); connections between subunits A–C and B–C (green).

HRESI(+)MS measurement established a molecular formula for **2** ([M + H]^+^ Dmmu ±0.0, C_27_H_34_N_3_O_6_). Comparison of the NMR (DMSO-*d*_6_) data for **2** (Table 1 and Table S4, and Figures S16–S21) with **1** revealed a near identical subunit A + C, with the only minor difference being a slightly lower ^13^C NMR chemical shift for C-17 in **2** (*δ*_C_ 166.1) compared with **1** (*δ*_C_ 170.6), while comparable ROESY correlations and chemical shifts revealed that **2** and **1** share a common relative configuration across subunit C (Figure 3, subunits A and C). The principle NMR differences between **2** and **1** were associated with subunit B, and included an increased level of aromatic substitution as evidenced by (i) a deshielded quaternary C-22 (*δ*_C_ 150.0) in **2** replacing H-22 (*δ*_H_ 7.35) in **1** and leaving only two ortho coupled aromatic protons H-20 (*δ*_H_ 7.66, d, *J* 9.0 Hz) and H-21 (*δ*_H_ 6.80, d, *J* 9.0 Hz) in **2**; and (ii) the absence of the ^1^H NMR resonance for the 19-OH, accompanied by significant changes in the ^13^C NMR chemical shifts for C-18 (D*d*_C_ + 14.5) and C-19 (D*d*_C_ − 9.9) in **2** versus **1** (Figure 3, subunit B). Collectively these observations suggest that **2** incorporates an amino-benzoxazole moiety in common with that reported for *N*-demethyl calcimycin (**6**). Unfortunately, the absence of reported NMR data for **6** precludes a comparison; however, a high level of concordance was observed in the ^1^H and ^13^C NMR (CDCl_3_) data for subunit B in **2** and calcimycin (**5**) [13] (Table S5). A ROESY correlation between H-3 and H-6 allowed assembly of the combined subunit A+C, mandating the final assembly of subunits A+C to B through a benzoxazole moiety (Figure 3, green highlight). In addition to biomimetic considerations, as the [α]_D_ (MeOH) for **2** (+78.8) compared well with that for the co-metabolite **1** (+65.2), we conclude that they share the same absolute configuration, allowing the structure for goondoxazole B (**2**) to be assigned as shown.

**Figure 4:**
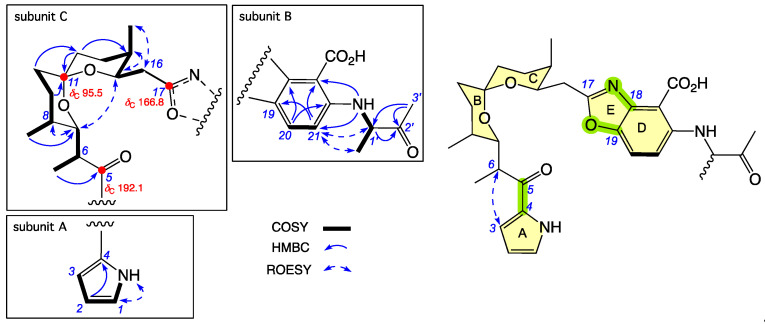
Selected 2D NMR (DMSO-*d*_6_) correlations and chemical shifts for **3**. Highlights: NMR chemical shifts (red); rings A–E (yellow); connections between subunits A–C and B–C (green).

HRESI(+)MS measurement established a molecular formula for **3** ([M + H]^+^ Dmmu –2.9, C_27_H_34_N_3_O_6_) requiring 14 DBE. Comparison of the NMR (DMSO-*d*_6_) data for **3** (Table 1 and Table S6, and Figures S25–S30) with **2** revealed identical subunits A, B and C, with the principal difference attributed to *N*-alkylation of subunit B with a 3-amino-2-butanone moiety, with the regiochemistry evident from HMBC correlations from a disubstituted amine (*δ*_H_ 8.44, 22-NH) to both C-21 and C-23, and ROESY correlations between H-21 and both H-1′ and 1′-Me (Figure 4). Based on biomimetic considerations and the [α]_D_ (MeOH) for **3** (+7.5), we proposed that **3** has the same absolute configuration as the co-metabolites **1** and **2**. That said, examination of the ^1^H NMR (methanol-*d*_4_) data for **3** (Figure S31) revealed a keto–enol tautomerism-mediated deuterium exchange of H-1′, requiring that goondoxazole C (**3**) exists as an equilibrating C-1′ epimeric (1:1) mixture, as indicated.

### 2.2. Reported Structure Family

**Figure 5:**
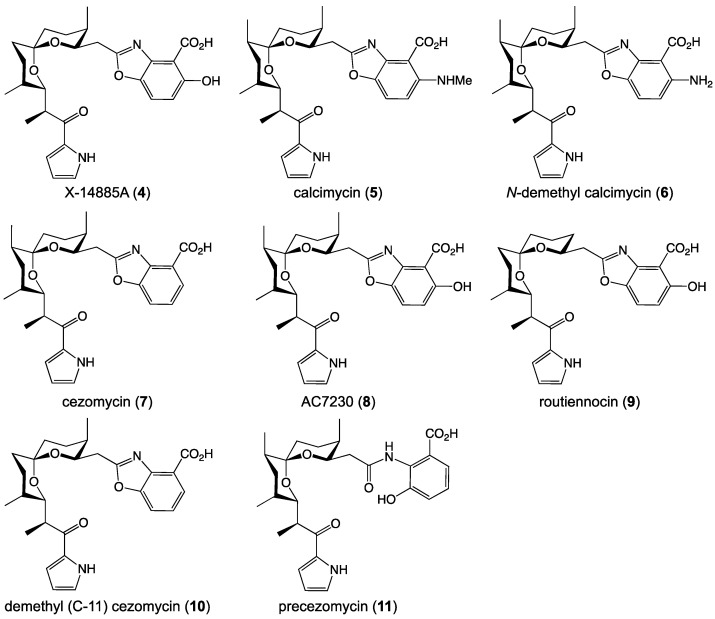
Known natural products belonging to calcimycin family.

The goondoxazoles A–C (**1**–**3**) belong to a relatively rare (but nevertheless well-known) class of PKS-NRPS-derived natural products, known examples of which are limited to only eight natural products (Figure 5). Calcimycin (A23187, **5**) was first reported in 1972 from *Streptomyces chartreusis* [14], with its structure and absolute configuration confirmed in 1974 by X-ray diffraction [15]. Since then, **5** has found broad application as a potent ionophore (calcium) and biochemical reagent used to probe cell physiology—being mentioned >14,000 publications and contributing to the understanding of calcium ions as second messengers, controlling muscle cell contraction, neurotransmitter release and other essential biological process. Additional natural product members of this structure class are limited to; *N*-demethyl calcimycin (**6**), reported in 1979 as a biotransformation product of **5** by *Streptomyces chartreusis* NRRL 3882 [16]; cezomycin (**7**), reported in 1982 from *Streptomyces chartreusis* NRRL 3882 with modified culture medium [13]; X-14885A (**4**), reported in 1983 from *Streptomyces* sp. X-14885 [11]; AC7230 (**8**), reported in 1987 from *Dactylosporangium* sp. AC7230 [17]; CP-61,405 first reported in 1988 from *Streptomyces routienni* [18], and renamed routiennocin (**9**) in a 1992 report on its total synthesis [19]; demethyl (C-11) cezomycin (**10**), first reported in 2003 with an incorrect structure from a *Frankia* sp. AiPs1 [20], and revised in 2013 by total synthesis [21]; and precezomycin (**11**), reported in 2024 from *Kitasatospora putterlickiae* [22].

### 2.3. Biological Assays and Parallel Studies

Goondoxazoles A–C (**1**–**3**) exhibit little to no growth inhibitory activity against human colorectal (SW620) and lung (NCI-H460) carcinoma cells, or developmental inhibition of the gastrointestinal nematode parasite *H. contortus* (Figure S56, Table 2 and Table S14). More significantly, and much like calcimycin (**5**), goondoxazoles A–C (**1**–**3**) are nM selective inhibitors of *D. immitis* mf and mM inhibitors of *D. immitis* L4 larvae motility (Table 2). These observations reveal a structure–activity relationship (SAR) where benzoxazoles **2** and **3** are significantly more potent against *D. immitis* mf than the substituted benzoic acid **1**. Interestingly, in parallel studies, we had encountered other benzoxazoles in extracts of other pasture soil-derived microbes, some prioritized for their activity against *D. immitis* mf.

**Figure 6:**
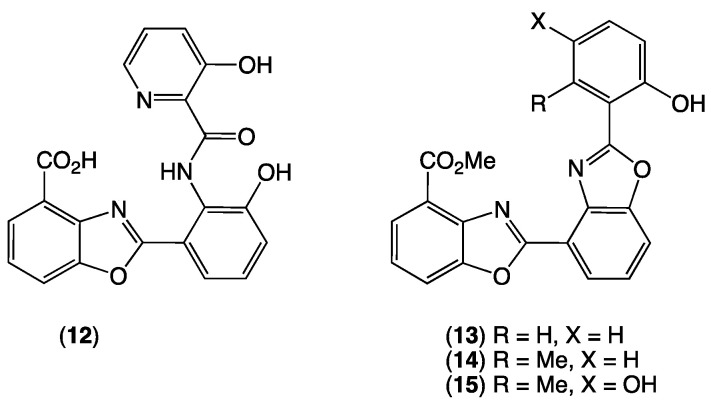
Known benzoxazole microbial natural products from *Streptomyces* spp. S4S-00200B03, CMB-MRB574 and CMB-GD066.

For example, fractionation of an M1 agar cultivation of the Goondicum pasture soil-derived *Streptomyces* sp. S4S-00200B03 (*D. immitis* mf, 96% at 25 mg/mL) yielded the anthelmintic principle as the known benzoxazole A-33583 (**12**) (Table S8 and Figures S34–S38), first reported in 1984 from the Alaskan soil-derived *Streptomyces* sp. NRRL 12068 [23]. Similarly, fractionation of an ISP2 agar plate cultivation of a New South Wales sheep pasture soil-derived *Streptomyces* sp. CMB-MRB574 (*D. immitis* mf, 100% at 25 mg/mL; *H. contortus* L1–L3 larvae, 97% at 25 mg/mL) yielded several classes of metabolite, including the known benzoxazole UK-1 (**13**) (Tables S9 and S10 and Figures S40–S44), first reported in 1993 from a Japanese soil *Streptomyces* sp. [24,25], and subsequently synthesized in 1997 [26], and revealed to be a magnesium ion-dependent DNA binding agent and inhibitor of human topoisomerase II in 1999 [27]. Finally, in a parallel investigation targeting new natural products, fractionation of an ISP2 cultivation of the Goondicum pasture soil-derived *Streptomyces* sp. CMB-GD066 yielded the known benzoxazole nataxazole (**14**) (Table S11 and Figures S45–S49), first reported in 2008 from a Brazilian soil-derived *Streptomyces* sp. Tü 6176 [28], and the subject of biosynthetic investigations in 2015 [29,30] and 2020 [31], along with the new analogue 5-hydroxynataxazole (**15**) (Table S12 and Figures S51–S54). While **13** and **14** displayed activity against *D. immitis* mf, this was at least 10-fold less than **2** and **3**. By contrast, **12** displayed comparable potency against *D. immitis* mf as **2** and **3**, but with significantly improved potency against *D. immitis* L4 larvae (Figure 6, Table 2).

In conclusion, an anthelmintic bioassay informed chemical analysis of the Australian pasture soil-derived *Streptomyces* sp. S4S-00193A39 yielded the active agents as new spiroketal polyketide alkaloids, goondoxazoles A–C (**1**–**3**). Goondoxazoles B (**2**) and C (**3**) were particularly noteworthy, displaying low nM potency against *D. immitis* mf (EC_50_ 55–85 nM) and low mM potency against *D. immitis* L4 larvae (EC_50_ ~2 mM), comparable to the structurally related and well-known ionophore calcimycin (**5**). The 16-–25-fold reduced potency against *D. immitis* mf exhibited by goondoxazole A (**1**), which lacked the benzoxazole moiety common to **2** and **3**, suggesting a possible SAR correlation. To explore this hypothesis, chemical investigation of two Australian pasture soil-derived microbes, also prioritized for activity against *D. immitis*, yielded the structurally simpler, achiral and known benzoxazoles, A-33583 (**12**) and UK-1 (**13**), with another Australian pasture soil-derived *Streptomyces* yielding the known and new benzoxazoles, nataxazole (**14**) and 5-hydroxynataxazole (**15**), respectively. Benzoxazoles **12**–**15** exhibited a selective inhibition of *D. immitis*, with A-33853 (**12**) being especially potent against *D. immitis* mf and L4 larvae—superior to the goondoxazoles. Of interest, a 2008 report described **12** as exhibiting comparable potency against the human blood parasite *Leishmania donovani* (responsible for the disease leishmaniasis) (IC_50_ 0.08 μM) [32]. This latter activity no doubt informed subsequent interest in A-33853, including reports in 2015 on the characterization of the biosynthetic gene cluster [30]; in 2021 on an *E. coli*-based biosynthetic platform to expand the structural diversity of natural benzoxazoles [33]; in 2022 on alternative benzoxazole assembly in anaerobic bacteria [34]; and in 2023 on heterologous production in the host bacterium *Myxococcus xanthus* [35]. Our discovery that benzoxazoles such as goondoxazoles and A-33853 exhibit potent and selective activity against the dog heartworm *D. immitis* reinforces the view that natural product benzoxazoles occupy privileged bioactive chemical space.

## 3. Materials and Methods

For general experimental details, see Supplementary Materials.

### 3.1. Collection of Soils and Isolating Microbes

Details on the collection of soil samples, and the isolation of microbes, are available in the Supplementary Materials.

### 3.2. Chemical Profiling (UPLC-DAD and UPLC-QTOF)

Details on chemical profiling (UPLC-DAD and UPLC-QTOF) are available in the Supplementary Materials.

### 3.3. Chemical Profiling (GNPS Molecular Networking)

Details on chemical profiling (GNPS molecular networking) are available in the Supplementary Materials.

### 3.4. Anthelmintic Activity Profiling

Extracts prepared from soil-derived microbes were subjected to a preliminary assessment of their ability to inhibit the motility of *D. immitis* mf, and *H. contortus* L1–L3 larvae development (see assay details below). This current report focuses on three prioritized bacterial isolates that exhibited promising levels of anthelmintic activity; *Streptomyces* sp. S4S-00193A39 (*D. immitis*, 100% at 2.5 µg/mL), *Streptomyces* sp. S4S-00200B03 (*D. immitis*, 96% at 25 mg/mL) and *Streptomyces* sp. CMB-MRB574 (*D. immitis*, 100% at 25 mg/mL, *H. contortus*, 97% at 25 mg/mL); and a fourth isolate, *Streptomyces* sp. CMB-GD066, that produced known and new benzoxazoles.

### 3.5. Taxonomy of S4S-00193A39, S4S-00200B03, CMB-MRB574 and CMB-GD066

Genomic DNA was extracted and analyzed and the data were used to build a phylogenetic tree to establish the taxonomy of S4S-00193A39, S4S-00200B03, CMB-GD066 and CMB-MRB574 using the protocols as summarized in the Supplementary Materials.

### 3.6. Phylogenetic Analysis of S4S-00193A39, S4S-00200B03, CMB-MRB574 and CMB-GD066

A phylogenetic tree obtained by PhyML (version 3.0) maximum likelihood analysis was constructed using the top similar 16S rRNA sequences displayed after BLAST on the Refseq RNA NCBI database using S4S-00193A39 16S rRNA as the query. The JC69 model was used to infer phylogeny sequences [36]. Sequence alignments were produced with the MUSCLE program [37]. A phylogenetic tree was constructed using the UGENE program using the aforementioned models and visualized using Ugene’s tree view [38]. BLAST analysis (NCBI database) showed that the amplified 16S rRNA sequence for S4S-00193A39 was a 99.49% match with *Streptomyces scabrisporus* strain 173877 (accession number: EU570570), prompting its taxonomic classification as *Streptomyces* sp. S4S-00193A39 (accession number: PV650902) (Figures S1 and S2). Using the same protocols, BLAST analysis (NCBI database) showed that the amplified 16S rRNA sequence for S4S-00200B03 was a 91.51% match with *Streptomyces macrosporeus* strain 1061 (accession number: HQ607419), prompting its taxonomic classification as *Streptomyces* sp. S4S-00200B03 (accession number: PV652627) (Figures S1 and S2); CMB-GD066 was a 99.43% match with *Streptomyces huiliensis* strain SCA2-4 (accession number: NR_181624), prompting its taxonomic classification as *Streptomyces* sp. CMB-GD066 (accession number: PV650423) (Figures S1 and S2); and CMB-MRB574 was a 98.81% match with *Streptomyces* sp. strain MK-30 (accession number: AB691771), prompting its taxonomic classification as *Streptomyces* sp. CMB-MRB574 (accession number: PV650434) (Figures S1 and S2).

### 3.7. Cultivation Profiling (MATRIX)

S4S-00193A39 was subjected to cultivation profiling in a 24-well plate (MATRIX) [9] to arrive at optimal cultivation conditions, using the protocols as summarized in the Supplementary Materials.

### 3.8. Scale-Up Cultivation and Fractionation of S4S-00193A39

Details on scale-up cultivation and fermentation of S4S-00193A39 are available in the Supplementary Materials.

*Goondoxazole A* (**1**). Brown oil; ${\left[\alpha \right]}_{D}^{24}$ + 110.9 (*c* 0.15, CHCl_3_), ${\left[\alpha \right]}_{D}^{24}$ + 65.2 (*c* 0.15, MeOH); 1D and 2D NMR (DMSO-*d*_6_) (Table 1 and Table S2, Figures S7–S12), 1D NMR (CDCl_3_) (Table S3, Figure S13 and S14); HRESIMS *m*/*z* 499.2459 [M + H]^+^ (calcd for C_27_H_35_N_2_O_7_, 499.2439).

*Goondoxazole B* (**2**). Brown oil; ${\left[\alpha \right]}_{D}^{24}$ − 99.0 (*c* 0.2, CHCl_3_), ${\left[\alpha \right]}_{D}^{24}$ + 78.8 (*c* 0.2, MeOH); 1D and 2D NMR (DMSO-*d*_6_) (Table 1 and Table S4, Figures S16–S21), 1D NMR (CDCl_3_) (Table S5, Figures S22 and S23); HRESIMS *m*/*z* 496.2442 [M + H]^+^ (calcd for C_27_H_34_N_3_O_6_, 496.2442). Note: embleyamycin D, which possesses a structure identical to goondoxazole B, was published while this manuscript was under review [39]. 

*Goondoxazole C* (**3**). Brown oil; ${\left[\alpha \right]}_{D}^{24}$ − 6.2 (*c* 0.1, CHCl_3_), ${\left[\alpha \right]}_{D}^{24}$ + 7.5 (*c* 0.1, MeOH); 1D and 2D NMR (DMSO-*d*_6_) (Table 1 and Table S6, Figures S25–S30), ^1^H NMR (methanol-*d*_4_) (Figure S31); HRESIMS *m*/*z* 566.2832 [M + H]^+^ (calcd for C_31_H_40_N_3_O_7_, 566.2861).

*Calcimycin* (**5**). Authentic standard of calcimycin (as free acid) was purchased from Life Technologies Australia Pty Ltd. (Scoresby, Australia). ${\left[\alpha \right]}_{D}^{24}$ − 48.3 (*c* 0.1, CHCl_3_), ${\left[\alpha \right]}_{D}^{24}$ + 58.4 (*c* 0.1, MeOH); ^1^H NMR (DMSO-*d*_6_) (Table S7, Figure S33).

### 3.9. Scale-Up Cultivation and Fractionation of S4S-00200B03

Details on scale-up cultivation and fermentation of S4S-00200B03 are available in the Supplementary Materials.

A-33853 (**12**). Light yellow amorphous powder; 1D and 2D NMR (DMSO-*d*_6_) (Table S8, Figures S34–S38); HRESIMS *m*/*z* 414.0711 [M + Na]^+^ (calcd for C_20_H_13_N_3_NaO_6_, 414.0697).

### 3.10. Scale-Up Cultivation and Fractionation of CMB-MRB574

Details on scale-up cultivation and fermentation of CMB-MRB574 are available in the Supplementary Materials.

UK-1 (**13**). Light yellow amorphous powder; 1D and 2D NMR (DMSO-*d*_6_) (Tables S9 and S10, Figures S40–S44); HRESIMS *m*/*z* 409.0803 [M + Na]^+^ (calcd for C_22_H_14_N_2_NaO_5_, 409.0795).

### 3.11. Scale-Up Cultivation and Fractionation of CMB-GD066

Details on scale-up cultivation and fermentation of CMB-GD066 are available in the Supplementary Materials.

*Nataxazole* (**14**). Light yellow amorphous powder; 1D and 2D NMR (DMSO-*d*_6_) (Table S11, Figures S45–S49); HRESIMS *m*/*z* 423.0966 [M + Na]^+^ (calcd for C_23_H_16_N_2_NaO_5_, 423.0951).

*5-hydroxynataxazole* (**15**). Light yellow amorphous powder; 1D and 2D NMR (DMSO-*d*_6_) (Table S12, Figures S51–S54); HRESIMS *m*/*z* 439.0903 [M + Na]^+^ (calcd for C_23_H_16_N_2_NaO_6_, 439.0901).

### 3.12. Cytotoxicity Assays

Details on cytotoxicity assays are available in the Supplementary Materials (Figure S56).

### 3.13. D. immitis Microfilariae Motility Inhibition Assay

Details on the *D. immitis* microfilariae motility inhibition assay are available in the Supplementary Materials.

### 3.14. Inhibition of Motility of D. immitis L4 Larvae Assay

Details on the inhibition of motility of *D. immitis* L4 larvae assay are available in the Supplementary Materials.

### 3.15. H. contortus L1–L3 Larvae Development Assay (LDA)

Details on the *H. contortus* L1–L3 larvae development assay (LDA) are available in the Supplementary Materials.

## 4. Conclusions

This study demonstrates that there is still much to learn from the defensive natural products that have evolved and are encoded within the livestock pasture microbiome. It also validates an integrated platform of biological, chemical and cultivation profiling as an efficient strategy for exploring bioactive microbial natural products. In particular, this study demonstrates the anthelmintic properties of a selection of pasture soil-derived *Streptomyces;* the new stereochemically complex spiroketal polyketide benzoxazoles, goondoxazoles B–C (**2**–**3**), against *D. immitis* mf; and the known and structurally far simpler achiral benzoxazole, A-33583 (**12**), against *D. immitis* mf and L4 life stages.

## Figures and Tables

**Table 1:** 1D NMR (DMSO-*d*_6_) data for **1**–**3**.

	(1)		(2)		(3)	
Pos.	*δ*_C_	*δ*_H_, Mult (*J* in Hz)	*δ*_C_	*δ*_H_, Mult (*J* in Hz)	*δ*_C_	*δ*_H_, Mult (*J* in Hz)
1	125.2	7.03, br s	125.0	7.01, br s	125.1	7.01, br s
2	109.5	6.14, ddd (*3.7*, *2.3*, *2.2*)	109.4	6.12, ddd (*3.6*, *2.4*, *2.3*)	109.4	6.12, ddd (*3.5*, *2.5*, *2.3*)
3	116.4	6.90, br s	116.2	6.84, br s	116.3	6.84, br s
4	132.6	-	132.6	-	132.6	-
5	192.7	-	192.1	-	192.2	-
6	42.0	3.20, dq (*10.3*, *6.9*)	41.6	3.09, dq (*10.3*, *6.9*)	41.6	3.08, dqd (*10.5*, *6.8*, *1.5*)
7	72.4	3.98, dd (*10.3*, *2.2*)	72.3	3.34 *, m	72.4	3.32 *, m
8	26.5	1.65, m	26.4	1.40 ^A^, m	26.4	1.39 ^A^, m
9	25.6	*a*. 2.01, dddd (*14.5*, *13.5*, *5.3*, *4.4*)	25.5	*a.* 1.74, dddd (*13.8*, *13.3*, *5.4*, *4.0*)	25.5	*a*. 1.74, m
		*b*. 1.28 ^A^, m		*b*. 1.22 ^B^, m		*b*. 1.23 ^B^, m
10	29.4	*a*. 1.45, ddd (*14.7*, *13.7*, *4.4*)	29.2	*a*. 1.41 ^A^, m	29.2	*a*. 1.41 ^A^, m
		*b*. 1.31 ^A^, m		*b*. 1.26 ^B^, m		*b*. 1.28 ^B^, m
11	95.5	-	95.5	-	95.5	-
12	29.1	*a*. 1.38 ^B^, m	28.9	*a*. 1.37 ^C^, m	28.9	*a*. 1.37 ^C^, m
		*b*. 1.13, m		*b*. 1.11 ^D^, m		*b*. 1.11 ^D^, m
13	25.3	*a*. 1.38 ^B^, m	25.3	*a*. 1.36 ^C^, m	25.3	*a*. 1.36 ^C^, m
		*b*. 1.04, m		*b*. 1.07 ^D^, m		*b*. 1.08 ^D^, m
14	29.0	1.53, m	29.5	1.54, m	29.5	1.54, m
15	67.4	4.14, ddd (*8.9*, *5.3*, *2.4*)	68.5	4.13, ddd (*9.6*, *4.0*, *3.2*)	68.5	4.13, m
16	40.9	*a*. 2.44, dd (*14.4*, *8.9*)	32.4	*a*. 2.94, dd (*14.6*, *9.6*)	32.5	*a*. 2.96, dd (*14.6*, *9.6*)
		*b*. 2.35, dd (*14.4*, *5.3*)		*b*. 2.90, dd (*14.6*, *4.0*)		*b*. 2.92, dd (*14.6*, *4.1*)
17	170.6	-	166.1	-	166.8	-
18	126.0	-	140.5	-	141.4	-
19	150.8	-	140.9	-	141.2	-
20	121.2	7.09, d (*8.0*)	116.8	7.66, d (*9.0*)	117.1	7.78, d (*9.0*)
21	125.7	7.13, dd (*8.0*, *7.7*)	114.1	6.80, d (*9.0*)	109.5	6.67, dd (*9.0*, *1.2*)
22	121.4	7.35, dd (*8.0*, *1.5*)	150.0	-	147.8	-
23	126.3	-	97.8	-	98.8	-
24	168.5	-	167.4	-	167.8	-
6-Me	13.4	0.83 ^C^, br d (*6.9*)	13.2	0.64, d (*6.9*)	13.2	0.61, dd (*7.2*, *6.8*)
8-Me	10.8	0.92, d (*6.9*)	10.7	0.85, d (*6.9*)	10.7	0.85, br d (*6.9*)
14-Me	11.1	0.83 ^C^, br d (*6.9*)	10.9	0.88, d (*6.9*)	10.9	0.89, br d (*7.0*)
1′	-	-	-	-	57.3	4.46, dq (*7.4*, *6.9*)
2′	-	-	-	-	208.4	-
3′	-	-	-	-	26.0	2.18, d (*0.8*)
1′-Me	-	-	-	-	17.3	1.38 ^C^, d (*6.9*)
19-OH	-	9.70, br s	-	-	-	-
1-NH	-	11.71, br s	-	11.76, br s	-	11.75, br s
22-NH	-	-	-	-	-	8.44, br t (*7.4*)

^A–D^ Resonances with the same superscript overlap, * resonance obscured by solvent.

**Table 2:** Anthelmintic activity of **1**–**3**, **5** and **12**–**15** (EC_50_ µM).

Compound	*D. immitis* mf Motility	*D. immitis* L4 Motility	*H. contortus* L1–L3 Development
goondoxazole A (**1**)	1.38	4.21	>25
goondoxazole B (**2**)	0.085	2.22	>25
goondoxazole C (**3**)	0.055	2.83	23
calcimycin (**5**)	0.027	0.25	25
A-33853 (**12**)	0.072	0.43	>25
UK-1 (**13**)	0.85	>25	>25
nataxazole (**14**)	0.75	>25	>25
5-hydroxynataxazole (**15**)	3.60	3.60	>25
gramicidin *	0.10	-	-
monensin *	-	0.40	-
ivermectin *	-	-	0.0005

- not tested, * positive controls.

## Data Availability

The raw NMR data for the new compounds, goondoxazoles A–C (**1**–**3**) and 5-hydroxynataxazole (**15**), have been deposited into the Natural Products Magnetic Resonance Database (NP-MRD; www.np-mrd.org).

## Supplementary Materials

The following supporting information can be downloaded at: https://www.mdpi.com/article/10.3390/antibiotics15030302/s1, bacterial taxonomy and phylogenetic analysis for *Streptomyces* spp. S4S-00193A39, S4S-00200B03, CMB-MRB574 and CMB-GD066, together with NMR and HRESIMS data for **1**–**3** and **12**–**15**.
