# Supplementary material for: Goondoxazoles A–C: Anthelmintic Spiroketal Polyketide Alkaloids and Other Benzoxazoles from Australian Pasture Soil-Derived Streptomyces spp
Source: Antibiotics (Basel). 2026 Mar 17;15(3):302. doi: 10.3390/antibiotics15030302 (PMC13024270; doi:10.3390/antibiotics15030302)
Supplement: Supplementary file 1 [file antibiotics-15-00302-s001.zip › antibiotics-4088268-supplementary.pdf]

SUPPORTING INFORMATION

Goondoxazoles A–C: Anthelmintic Spiroketal Polyketide Alkaloids and Other Benzoxazoles from Australian Pasture Soil-Derived *Streptomyces* spp.

Shengbin Jin,[†] David F Bruhn,[‡] Erica J. Burkman,[‡] Cynthia T. Childs,[‡] Jianying Han,[†] Zeinab G. Khalil,[†] Yovany Moreno,[‡] Angela A. Salim,[†] Kaumadi Samarasekera,[†] Marcelo M.P. Tangerina,[†§] and Robert J. Capon[†*]

[†]Institute for Molecular Bioscience, The University of Queensland, St Lucia, QLD 4072, Australia

[‡] Boehringer Ingelheim Animal Health, USA Inc. 1730 Olympic Drive, Athens, GA 30601, USA

[§] Botany Department, Institute of Biosciences, University of São Paulo-USP, São Paulo 05508-090, SP, Brazil.

* Corresponding author. Tel.: +61 7 3346 2980; e-mail: r.capon@uq.edu.au

List of figures

Figure S1. 16S rRNA sequences of *Streptomyces* sp. S4S-00193A39, S4S-00200B03, CMB-MRB574 and CMB-GD066, and their BLAST search (closest match) on 16S rRNA sequences. 8
Figure S2. Phylogenetic tree of S4S-00193A39, S4S-00200B03, CMB-MRB574 and CMB-GD066........ 9
Figure S3. UPLC-DAD (210 nm) chromatogram of the EtOAc extract of an ISP2 cultivation of *Streptomyces* sp. S4S-00193A39. 11
Figure S4. GNPS analysis of S4S-00193A39 with compounds related nodes. (A): GNPS molecular network for the library of ×704 isolates form ×19 Goondicum soil samples; (B): GNPS molecular network for an expanded Capon lab library of an additional ×1957 microbial extracts. 12
Figure S5. Top: UPLC-DAD chromatograms of a media composition study for S4S-00193A39 in (A) D400, (B) 333, (C) SD, (D) CG, (E) ISP2, (F) M1; (peaks painted by the identical colour with their UV-vis spectrums). i: agar; ii: static broth; iii: shaking broth; iv: media blank; *: calibrant: Fmoc-Phe-OH, 20 μg/mL. Bottom: image of MATRIX culture plates (a: agar, b: static broth, c: shaking broth). 13
Figure S6. Isolation scheme for 100 plates of 333 agar medium cultivation of S4S-00193A39. (a) Trituration [Hexane (-1-1), DCM (-1-2) and MeOH (-1-3)], (b) Prep HPLC; Prep C_8, Zorbax RX-C_8, 10-100% MeCN/H_2O (0.01% TFA) in 20 mins, 20 mL/min, 40 fractions, (c) Semi-prep HPLC; Semi-prep C_{18}, Zorbax SB-C_{18}, 65% MeCN/H_2O (0.01% TFA) in 20 mins, 3 mL/min, (d) Semi-prep HPLC; Semi-prep C_{18}, Zorbax SB-C_{18}, 60% MeCN/H_2O (0.01% TFA) in 20 mins, 3 mL/min........ 14
Figure S7. ^{1}H NMR (600 MHz, DMSO-d_6) spectrum of goondoxazole A (**1**)........ 17
Figure S8. ^{13}C NMR (150 MHz, DMSO-d_6) and UV-vis (inset) spectra of goondoxazole A (**1**)........ 17
Figure S9. HSQC NMR (DMSO-d_6) spectrum of goondoxazole A (**1**). 18
Figure S10. HMBC NMR (DMSO-d_6) spectrum of goondoxazole A (**1**). 18
Figure S11. COSY NMR (DMSO-d_6) spectrum of goondoxazole A (**1**). 19
Figure S12. ROESY NMR (DMSO-d_6) spectrum of goondoxazole A (**1**)........ 19
Figure S13. ^{1}H NMR (600 MHz, $CDCl_3$) spectrum of goondoxazole A (**1**). 21
Figure S14. ^{13}C NMR (150 MHz, $CDCl_3$) spectrum of goondoxazole A (**1**). 21
Figure S15. HRESIMS spectrum for goondoxazole A (**1**)........ 22
Figure S16. ^{1}H NMR (600 MHz, DMSO-d_6) spectrum of goondoxazole B (**2**)........ 24
Figure S17. ^{13}C NMR (150 MHz, DMSO-d_6) and UV-vis (inset) spectra of goondoxazole B (**2**)........ 24
Figure S18. HSQC NMR (DMSO-d_6) spectrum of goondoxazole B (**2**). 25
Figure S19. HMBC NMR (DMSO-d_6) spectrum of goondoxazole B (**2**). 25
Figure S20. COSY NMR (DMSO-d_6) spectrum of goondoxazole B (**2**). 26
Figure S21. ROESY NMR (DMSO-d_6) spectrum of goondoxazole B (**2**)........ 26
Figure S22. ^{1}H NMR (600 MHz, $CDCl_3$) spectrum of goondoxazole B (**2**). 28
Figure S23. ^{13}C NMR (150 MHz, $CDCl_3$) spectrum of goondoxazole B (**2**)........ 28
Figure S24. HRESIMS spectrum for goondoxazole B (**2**)........ 29
Figure S25. ^{1}H NMR (600 MHz, DMSO-d_6) spectrum of goondoxazole C (**3**)........ 31
Figure S26. ^{13}C NMR (150 MHz, DMSO-d_6) and UV-vis (inset) spectra of goondoxazole C (**3**)........ 31
Figure S27. HSQC NMR (DMSO-d_6) spectrum of goondoxazole C (**3**). 32
Figure S28. HMBC NMR (DMSO-d_6) spectrum of goondoxazole C (**3**). 32
Figure S29. COSY NMR (DMSO-d_6) spectrum of goondoxazole C (**3**). 33
Figure S30. ROESY NMR (DMSO-d_6) spectrum of goondoxazole C (**3**)........ 33
Figure S31. ^{1}H NMR (600 MHz, methanol-d_4) spectrum of goondoxazole C (**3**). 34
Figure S32. HRESIMS spectrum for goondoxazole C (**3**)........ 35
Figure S33. ^{1}H NMR (600 MHz, DMSO-d_6) spectrum of calcimycin (**5**). 37
Figure S34. ^{1}H NMR (600 MHz, DMSO-d_6) spectrum of A-33853 (**12**). 39
Figure S35. ^{13}C NMR (150 MHz, DMSO-d_6) spectrum of A-33853 (**12**)........ 39
Figure S36. HSQC NMR (DMSO-d_6) spectrum of A-33853 (**12**). 40
Figure S37. HMBC NMR (DMSO-d_6) spectrum of A-33853 (**12**). 40
Figure S38. COSY NMR (DMSO-d_6) spectrum of A-33853 (**12**). 41
Figure S39. HRESIMS spectrum for A-33853 (**12**)........ 42
Figure S40. ^{1}H NMR (600 MHz, DMSO-d_6) spectrum of UK-1 (**13**)........ 44
Figure S41. ^{1}H NMR (600 MHz, $CDCl_3$) spectrum of UK-1 (**13**). 45
Figure S42. HSQC NMR (DMSO-d_6) spectrum of UK-1 (**13**)........ 45
Figure S43. HMBC NMR (DMSO-d_6) spectrum of UK-1 (**13**)........ 46

Figure S44. COSY NMR (DMSO-d_6) spectrum of UK-1 (**13**). 46
Figure S45. ^{1}H NMR (600 MHz, DMSO-d_6) spectrum of nataxazole (**14**). 48
Figure S46. ^{13}C NMR (150 MHz, DMSO-d_6) spectrum of nataxazole (**14**). 48
Figure S47. HSQC NMR (DMSO-d_6) spectrum of nataxazole (**14**). 49
Figure S48. HMBC NMR (DMSO-d_6) spectrum of nataxazole (**14**). 49
Figure S49. COSY NMR (DMSO-d_6) spectrum of nataxazole (**14**). 50
Figure S50. HRESIMS spectrum for nataxazole (**14**). 51
Figure S51. ^{1}H NMR (600 MHz, DMSO-d_6) spectrum of 5-hydroxynataxazole (**15**). 53
Figure S52. HSQC NMR (DMSO-d_6) spectrum of 5-hydroxynataxazole (**15**). 53
Figure S53. HMBC NMR (DMSO-d_6) spectrum of 5-hydroxynataxazole (**15**). 54
Figure S54. COSY NMR (DMSO-d_6) spectrum of 5-hydroxynataxazole (**15**). 54
Figure S55. HRESIMS spectrum for 5-hydroxynataxazole (**15**). 55
Figure S56. Cytotoxicity of **1–3**, **5** and **12-15**. 58

List of tables

Table S1. MATRIX media compositions. 10
Table S2. 1D and 2D NMR (DMSO-d_6) data for goondoxazole A (**1**). 16
Table S3.1D NMR ($CDCl_3$) comparison of goondoxazole A (**1**) and X-14885A (**4**). 20
Table S4. 1D and 2D NMR (DMSO-d_6) data for goondoxazole B (**2**). 23
Table S5. ^{13}C NMR ($CDCl_3$) comparison of goondoxazole B (**2**) and calcimycin (**5**). 27
Table S6. 1D and 2D NMR (DMSO-d_6) data for goondoxazole C (**3**). 30
Table S7. ^{1}H NMR (DMSO-d_6) data for goondoxazole A (**1**) and calcimycin (**5**). 36
Table S8. 1D and 2D NMR (DMSO-d_6) data for A-33853 (**12**). 38
Table S9. 1D and 2D NMR (DMSO-d_6) data for UK-1 (**13**). 43
Table S10. ^{1}H NMR ($CDCl_3$) for UK-1 (**13**) and literature data for UK-1 44
Table S11. 1D and 2D NMR (DMSO-d_6) data for nataxazole (**14**). 47
Table S12. 1D and 2D NMR (DMSO-d_6) data for 5-hydroxynataxazole (**15**). 52
Table S13.The rotation of **1–3** and calcimycin before and after treated metal ion. 56
Table S14. Cytotoxicity of **1–3**, **5** and **12-15** (IC_{50} μM). 59

General experimental details

Chiroptical measurements ($[\alpha]_D$) were obtained on a JASCO P-1010 polarimeter in a 100 × 2 mm cell at specified temperatures. ECD spectra were acquired on a Jasco J-810 spectropolarimeter (163–900 nm) as 0.5 mg/mL solutions in MeOH. Nuclear magnetic resonance (NMR) spectra were acquired on a Bruker Avance 600 MHz spectrometer with a ^{1}H optimized 5mm triple resonance TCI CryoProbe (He) designed for ^{1}H observation with ^{13}C and ^{15}N decoupling. In all cases spectra were acquired at 25°C in solvents as specified with referencing to residual solvent ^{1}H or ^{13}C NMR resonances (DMSO-d_6: δ_H 2.50 and δ_C 39.5; $CDCl_3$: δ_H 7.26 and δ_C 77.2). High-resolution ESIMS spectra were obtained on a Bruker micrOTOF mass spectrometer by direct injection in MeOH at 3 µL/min using sodium formate clusters as an internal calibrant. High performance liquid chromatography-diode array-mass spectrometry (HPLC-DAD-MS) data were acquired on an Agilent 1260 series separation module equipped with a diode array detector and an Agilent G6125B series LC/MSD mass detector (Agilent Poroshell 120 SB-C_8 2.7 µm, 3.0 × 150 mm column, gradient elution at 0.8 mL/min over 6.5 min from 90% H_2O/MeCN to 100% MeCN with a constant 0.05% formic acid/MeCN modifier). Semi-preparative HPLCs were performed using Agilent 1100 series HPLC instruments with corresponding detectors, fraction collectors and software inclusively. UPLC chromatograms were obtained on Agilent 1290 infinity UPLC system equipped with a diode array multiple wavelength detector (Zorbax SB-C_8 RRHD 1.8 µm, 2.1 × 50 mm column, gradient elution at 0.417 mL/min over 2.50 min from 90% H_2O/MeCN to 100% MeCN with a constant 0.01% TFA/MeCN modifier). UPLC-QTOF analysis was performed on UPLC-QTOF instrument comprising of an Agilent 1290 Infinity II UPLC (Zorbax SB-C_8 RRHD 1.8 µm, 2.1 × 50 mm column, gradient elution at 0.417 mL/min over 2.50 min from 90% H_2O/MeCN to 100% MeCN with a constant 0.1% formic acid/MeCN modifier) coupled to an Agilent 6545 Q-TOF. MS/MS analysis was performed on the same instrument for ions detected in the full scan at an intensity above 1000 counts at 10 scans/s, with an isolation width of 4 ~*m/z* using a fixed collision energy and a maximum of 3 selected precursors per cycle. Chemicals were purchased from Merck unless otherwise specified. Analytical-grade solvents were used for solvent extractions. Chromatography solvents were of HPLC grade supplied by Merck and filtered/degassed through 0.45 µm polytetrafluoroethylene (PTFE) membrane prior to use. Deuterated solvents were purchased from Cambridge Isotopes. Microorganisms were manipulated

under sterile conditions using a Laftech class II biological safety cabinet and incubated in either MMM Friocell incubators (Lomb Scientific) or an Innova 42R incubator shaker (John Morris).

Collection of soils and isolating microbes

Soil samples (×103) were collected from Goondicum Pastoral, a cattle station situated in an extinct volcanic crater near the headwaters of the Burnett River, Queensland, Australia, in part under the auspices of the Australian Soils for Science (S4S) citizen science initiative, as well as from multiple sheep stations across Australia. Soils were used to inoculate ISP2 and M1 agar mother plates, which after incubation at 27 °C for 14 days, and manual colony picking, yielded isolates (×1,893) that were cultivated on fresh ISP2 or M1 agar plates (media choice based on that of the source mother plate). All microbes were cryopreserved at –80 °C, and an EtOAc extract prepared from the single agar plate cultivation was dried, resuspended in DMSO and archived at –20 °C.

Taxonomic identification

Genomic DNA was extracted from an ISP2 agar plate cultivation of target bacteria using the DNeasy Blood & Tissue Kit (Qiagen) as per the manufacturer's protocol. The 16S rRNA genes q amplified by PCR using the universal primers 27F (5'-AGAGTTTGATCCTGGCTCAG-3') and 1492R (5'- TACGGCTACCTTCTTACGACTT-3') purchased from Sigma-Aldrich. The PCR mixture (50 µL) contained genomic DNA (2 µL, 20–40 ng), EmeraldAmp GT PCR Master Mix (2XPremix) (25 µL), primer (0.2 µM, each), and H_2O (up to 50 µL). PCR was performed using the following conditions: initial denaturation at 95°C for 2 min, 40 cycles in series of 95°C for 20 s (denaturation), 56°C for 20 s (annealing) and 72°C for 30 s (extension), followed by one cycle at 72°C for 5 min. The PCR products were purified with PCR purification kit (Qiagen) and sequenced.

S4S-00193A39

TGCAAGTCGAACGCTGAAGCCCTT-
GGGGTGGATGAGTGGCGAACGGGTGAGTAACACGTGGGCAACCTGCCCTGCACTCTGGGATAACTTC
GGGAAACCGGAGCTAATACCGGATAACATCCTCCTCCGCATGGTGGGGGGTTGAAAGTTCCGGCGGTG
CAGGATGGGCCCGCGGCCTATCAGCTTGTTGGTGGGGTAGTGGCCTACCAAGGCGACGACGGGTAGCC
GGCCTGAGAGGGCGACCGGCCACACTGGGACTGAGACACGGCCCAGACTCCTACGGGAGGCAGCAGT
GGGGAATATTGCGCAATGGGCGAAAGCCTGACGCAGCGACGCCGCGTGAGGGATGACGGCCTTCGGG
TTGTAAACCTCTTTCAGCAGGGAAGAAGCGAAAGTGACGGTACCTGCAGAAGAAGCACCGGCTAACT
ACGTGCCAGCAGCCGCGGTAATACGTAGGGTGCGAGCGTTGTCCGGAATTATTGGGCGTAAAGAGCTC
GTAGGCGGCCTGTCGCGTCGGATGTGAAAACTCGGGGCTTAACCCCGAGCCTGCATTCGATACGGGCA
GGCTAGAGTTCGGCAGGGGAGACTGGAATTCCTGGTGTAGCGGTGAAATGCGCAGATATCAGGAGGA
ACACCGGTGGCGAAGGCGGGTCTCTGGGCCGATACTGACGCTGAGGAGCGAAAGCGTGGGGAGCGAA
CAGGATTAGATACCCTGGTAGTCCACGCCGTAAACGTTGGGAACTAGGTGTGGGCGACATTCCACGTC
GTCCGTGCCGCAGCTAACGCATTAAGTTCCCCGCCTGGGGAGTACGGCCGCAAGGCTAAAACTCAAAG
GAATTGACGGGGGCCCGCACAAGAGGCGGAGCATGTGGCTTAATTCGACGCAACGCGAAGAACCTTA
CCAAGGCTTGACATACACCGGAAACATCTGGAGACAGGTGCCCCTTTTGGTCGGTGTACAGGTGGTGC
ATGGCTGTCGTCAGCTCGTGTCGTGAGATGTTGGGTTAAGTCCCGCAACGAGCGCAACCCTCGTTCTGT
GTTGCCAGCATGCCCTTCGGGGTGATGGGGACTCACAGGAGACTGCCGGGGTCAACTCGGAAGAAGG
TGGGGACGACGTCAAGTCATCATGCCCCTTATGTCTGGGCTGC-ACGTGCTACATGGCCGG

Description	Scientific Name	Max Score	Total Score	Query Cover	E value	Per. Ident	Acc. Len	Accession
Streptomyces scabrisporus strain 173877 16S ribosomal RNA gene, partial sequence	Embleya scabrispora	2122	2122	100%	0.0	99.49%	1445	EU570570.1
Embleya scabrispora strain NBRC 100760 16S ribosomal RNA, partial sequence	Embleya scabrispora	2122	2122	100%	0.0	99.49%	1449	NR_112597.1
Embleya sp. NBC_00888 chromosome, complete genome	Embleya sp. NBC_00888	2122	8486	100%	0.0	99.49%	8051820	CP108784.1
Streptomyces scabrisporus strain HBUM174877 16S ribosomal RNA gene, partial sequence	Embleya scabrispora	2122	2122	100%	0.0	99.49%	1432	EU841545.1
Embleya scabrispora strain QTP241032 16S ribosomal RNA gene, partial sequence	Embleya scabrispora	2117	2117	100%	0.0	99.40%	1432	PP837087.1
Embleya scabrispora strain BCCO 10_1496 16S ribosomal RNA gene, partial sequence	Embleya scabrispora	2117	2117	100%	0.0	99.40%	1411	KP718597.1
Streptomyces scabrisporus strain HBUM174859 16S ribosomal RNA gene, partial sequence	Embleya scabrispora	2117	2117	100%	0.0	99.40%	1431	FJ486391.1
Embleya sp. NBC_00896 chromosome, complete genome	Embleya sp. NBC_00896	2117	8463	100%	0.0	99.40%	7405279	CP108776.1
Embleya sp. AN120556 16S ribosomal RNA gene, partial sequence	Embleya sp. AN120556	2117	2117	100%	0.0	99.40%	1397	PP099785.1
Streptomyces sp. strain NF-813 16S ribosomal RNA gene, partial sequence	Streptomyces sp.	2117	2117	100%	0.0	99.40%	1478	MH362775.1
Streptomyces sp. MI-5.1 P51 partial 16S rRNA gene, isolate MI-5.1 P51	Streptomyces sp. MI-5.1 P51	2117	2117	100%	0.0	99.40%	1476	FN550137.1
Streptomyces scabrisporus strain Bn035 16S ribosomal RNA gene, partial sequence	Embleya scabrispora	2117	2117	100%	0.0	99.40%	1406	KC440853.1
Streptomyces sp. MI-5.1 P62 partial 16S rRNA gene, isolate MI-5.1 P62	Streptomyces sp. MI-5.1 P62	2117	2117	100%	0.0	99.40%	1476	FN550140.1

S4S-00200B03

CTTACCATGCAAGTCGAACCGATGAATCCACTGTAAGTGGGGATTAGTGGCGAACGGGTGAGTAACCC
GTTGGGCAATCTGCCCTGCACTCTGGGACAAGCCCTGGAAACGGGGTCTAATACCGGATACTGACCCG
CCTGGGCATCCAGGCGGTTCAAAAGCTCCGGCGGTGCAGGATGACCCCGCGGCCTATCACCTTGTTGG
TGAGGTAACGGCTCACCAAGGCAACAACGGGTAGCCGGCCTGAAAGGGCAACCGGCCACACTGGGAC
TGAAACACGGCCCAAACTCCTACGGGAGGCAGCAGGGGGGAATATTGCACAAGGGGCAAAAGCCTGA
TGCACCGACCCCGCGTGAGGAATGACGGCCTTCGGGTTGTAAACCTCTTTCAGCAGGGAAAAAGCGAA
AGTGACGGTACCTGCAAAAAAACCGCCGGCTAACTACGTGCCAGCACCCGCGGTAATACGTAGGGCG
CGAGCGTTGTCCGGAATTATTGGGCGTAAAAAGCTCGTAGGCGGTTTGTCCCGTCGGTTGTGAAAGCC
CGGGGCTTAACCCCGGGTCTGCATTCAATACGGGCAGGCTAGAGTTCGGTAGGGAAAATCGAAATTCC
TGGTGTAGCGGTGAAATGCCCAGATATCAGGAGGAACACCGGTGGCAAAGGCGGATCTCTGGGCCAA
TACTGACCCTGAGGAGCGAAAGCGTGGGGAGCGAACAGGATTAGAATACCCTGGTAGTCCACGCCGT
AAAACGGGTGGGAAACTAGGGTGTGGGGCGACATTCCACGTCGTCCGGTGCCCGCAGCTAAACGCAT
TTAAGGTGCCCCCGCCCTGGGGGAAGTACGGGCCCGCAAGGGCTTAAAAACCTCAAAAGGGAAATTT
GAACGGGGGGGCCCCGCCACAAAGCGGGCGGAAGCCATGGTGGGCTTTAATTTCGAACGCCAACCGC
CGAAAGAAACCCTTTACCCAAGGGCTTTGAACATTACCACCCGGGAAAAACCGTTCTGGGAAGAACA
GGGCCGCCCCCCTTT

	Description	Scientific Name	Max Score	Total Score	Query Cover	E value	Per. Ident	Acc. Len	Accession
☐	Actinobacterium C7 16S ribosomal RNA gene, partial sequence	actinobacterium C7	1291	1291	95%	0.0	91.56%	1439	HM209312.1
☐	Streptomyces macrosporeus strain 1061 16S ribosomal RNA gene, partial sequence	Streptomyces macrosporeus	1282	1282	94%	0.0	91.51%	1473	HQ607419.1
☐	Streptomyces albogriseolus strain HBUM83454 16S ribosomal RNA gene, partial sequence	Streptomyces albogriseolus	1275	1275	95%	0.0	91.25%	1441	EU841557.1
☐	Streptomyces macrosporeus strain 1169 16S ribosomal RNA gene, partial sequence	Streptomyces macrosporeus	1273	1273	90%	0.0	92.14%	1478	HQ607434.1
☐	Streptomyces macrosporeus strain 1172 16S ribosomal RNA gene, partial sequence	Streptomyces macrosporeus	1271	1271	96%	0.0	90.95%	1477	HQ607435.1
☐	Streptomyces atrovirens strain 5-2 16S ribosomal RNA gene, partial sequence	Streptomyces atrovirens	1266	1266	94%	0.0	91.29%	1423	KJ571043.1
☐	Streptomyces sp. GX9 16S ribosomal RNA gene, partial sequence	Streptomyces sp. GX9	1262	1262	93%	0.0	91.49%	1410	JF830633.1
☐	Streptomyces sp. MS-1 16S ribosomal RNA gene, partial sequence	Streptomyces sp. SLBN-134	1251	1251	95%	0.0	90.83%	1399	JN578482.1
☐	Streptomyces macrosporeus strain 1162 16S ribosomal RNA gene, partial sequence	Streptomyces macrosporeus	1251	1251	95%	0.0	90.83%	1471	HQ607431.1
☐	Streptomyces sp. 3187 16S ribosomal RNA gene, partial sequence	Streptomyces sp. 3187	1249	1249	95%	0.0	90.75%	1490	DQ663148.1
☐	Streptomyces fungicidicus strain TXX3120 chromosome, complete genome	Streptomyces fungicidicus	1249	7373	97%	0.0	90.48%	6740768	CP023407.1
☐	Streptomyces sp. MBRC-34 16S ribosomal RNA gene, partial sequence	Streptomyces sp. MBRC-34	1249	1249	95%	0.0	90.75%	1513	KC179795.1
☐	Streptomyces macrosporeus strain 14312 16S ribosomal RNA gene, partial sequence	Streptomyces macrosporeus	1249	1249	95%	0.0	90.75%	1490	EF371436.1
☐	Streptomyces sp. strain 6A 16S ribosomal RNA gene, partial sequence	Streptomyces sp.	1249	1249	95%	0.0	90.75%	1462	MK456473.1
☐	Streptomyces fungicidicus strain F9 chromosome, complete genome	Streptomyces fungicidicus	1249	7373	97%	0.0	90.48%	6740828	CP086175.1
☐	Streptomyces macrosporeus strain 52 16S ribosomal RNA gene, partial sequence	Streptomyces macrosporeus	1249	1249	95%	0.0	90.75%	1490	EF063469.1

CMB-MRB574

TGACAAGTCGTACGATGAACCACTTCGGTGGGGATTAGTGGCGAACGGGTGAGTAACCACCGTGGGC
AATCTGCCCTGCACTCTGGGACAAGCCCTGGAAACGGGGTCTAATACCGGATACGAGTCTCCAAGGCA
TCTTGGAGACTGTAAAGCTCCGGCGGTGCAGGATGAGCCCGCGGCCTATCAGCTTGTTGGTGAGGTAG
TGGCTCACCAAGGCGACGACGGGTAGCCGGCCTGAGAGGGCGACCGGCCACACTGGGACTGAGACAC
GGCCCAGACTCCTACGGGAGGCAGCAGTGGGGAATATTGCACAATGGGCGAAAGCCTGATGCAGCGA
CGCCGCGTGAGGGATGACGGCCTTCGGGTTGTAAACCTCTTTCAGCAGGGAAGAAGCGAGAGTGACG
GTACCTGCAGAAGAAGCGCCGGCTAACTACGTGCCAGCAGCCGCGGTAATACGTAGGGCGCAAGCGT
TGTCCGGAATTATTGGGCGTAAAGAGCTCGTAGGCGGCTTGTCACGTCGGTTGTGAAAGCCCGGGGCT
TAACCCCGGGTCTGCAGTCGATACGGGCAGGCTAGAGTTCGGTAGGGGAGATCGGAATTCCTGGTGTA
GCGGTGAAATGCGCAGATATCAGGAGGAACACCGGTGGCGAAGGCGGATCTCTGGGCCGATACTGAC
GCTGAGGAGCGAAAGCGTGGGGAGCGAACAGGATTAGATACCCTGGTAGTCCACGCCGTAAACGGTG
GGCACTAGGTGTGGGCGACATTCCACGTCGTCCGTGCCGCAGCTAACGCATTAAGTGCCCCGCCTGGG
GAGTACGGCCGCAAGGCTAAAACTCAAAGGAATTGACGGGGGCCCGCACAAGCGGCGGAGCATGTGG
CTTAATTCGACGCAACGCGAAGAACCTTACCAAGGCTTGACATACACCGGAAAACTCTGGAGACAGG
GTCCCCCTTGTGGTCGGTGTACAGGTGGTGCATGGCTGTCGTCAGCTCGTGTCGTGAGATGTTGGGTTA
AGTCCCGCAACGAGCGCAACCCTTGTCCCGTGTTGCCAGCAGGCCCTTGTGGTGCTGGGGACTCACGG
GAGACCGCCGGGGTCAACTCGGAAGAAGGTGGGGACGACGTCAAGTCATCATGCCCCTTATGTCTTGG
GCTGCCACGTGCTACAATGGCCGGTACATGAGCTGCGA-C-
CGTGAGGTGGAACGAATCTCAAAAAGCCGGTCTCATTTCGAATGGGG-
TGCAACTCGACCCCTGAAGTCGGAATCC

	Description	Scientific Name	Max Score	Total Score	Query Cover	E value	Per. Ident	Acc. Len	Accession
☐	Streptomyces sp. MK-30 gene for 16S rRNA, partial sequence	Streptomyces sp. MK-30	2241	2241	99%	0.0	98.81%	1433	AB691771.1
☐	Streptomyces sp. SAT1, complete genome	Streptomyces sp. SAT1	2241	13441	100%	0.0	98.74%	7472530	CP015849.1
☐	Streptomyces sp. MK-19 gene for 16S ribosomal RNA, partial sequence	Streptomyces sp. MK-19	2235	2235	99%	0.0	98.73%	1487	AB770481.1
☐	Streptomyces sp. HW31 16S ribosomal RNA gene, partial sequence	Streptomyces sp. HW31	2230	2230	99%	0.0	98.65%	1424	KF194345.1
☐	Streptomyces andamanensis strain KC-112 16S ribosomal RNA, partial sequence	Streptomyces andamanensis	2230	2230	99%	0.0	98.65%	1500	NR_149230.1
☐	Streptomyces andamanensis strain T33M3 16S ribosomal RNA gene, partial sequence	Streptomyces andamanensis	2230	2230	99%	0.0	98.65%	1447	MT367757.1
☐	Streptomyces sp. PsTaAH124 16S ribosomal RNA gene, partial sequence	Streptomyces sp. PsTaAH124	2209	2209	99%	0.0	98.41%	1378	KJ889047.1
☐	Streptomyces sp. gene for 16S rRNA, partial sequence, strain: NBRC 13931	Streptomyces sp.	2207	2207	99%	0.0	98.34%	1453	AB184550.1
☐	Streptomyces andamanensis strain PCU 347 16S ribosomal RNA gene, partial sequence	Streptomyces andamanensis	2207	2207	99%	0.0	98.57%	1363	MT760033.1
☐	Streptomyces sp. Xyl84 DNA, chromosome 1, complete sequence	Streptomyces sp. Xyl84	2207	13225	100%	0.0	98.26%	7211145	AP029182.1
☐	Streptomyces sahachiroi gene for 16S rRNA, partial sequence, strain: NBRC 13928	Streptomyces sahachiroi	2207	2207	99%	0.0	98.34%	1476	AB184548.1
☐	Streptomyces andamanensis strain PCU 347 16S ribosomal RNA gene, partial sequence	Streptomyces andamanensis	2202	2202	99%	0.0	98.49%	1364	MT758159.1
☐	Streptomyces andamanensis strain I-SB-06 16S ribosomal RNA gene, partial sequence	Streptomyces andamanensis	2198	2198	98%	0.0	98.71%	1318	OR243788.1

CMB-GD066

TGCAAGTCGAACGATGAACCTCCTTCGGGAGGGGATTAGTGGCGAACGGGTGAGTAACACGTGGGCA
ATCTGCCCTGCACTCTGGGACAAGCCCTGGAAACGGGGTCTAATACCGGATACGACCACTGAGCGCAT
GCTCGGTGGTGGAAAGCTCCGGCGGTGCAGGATGAGCCCGCGGCCTATCAGCTTGTTGGTGGGGTGAT
GGCCTACCAAGGCGACGACGGGTAGCCGGCCTGAGAGGGCGACCGGCCACACTGGGACTGAGACACG
GCCCAGACTCCTACGGGAGGCAGCAGTGGGGAATATTGCACAATGGGCGAAAGCCTGATGCAGCGAC
GCCGCGTGAGGGATGACGGCCTTCGGGTTGTAAACCTCTTTCAGCAGGGAAGAAGCGAAAGTGACGG
TACCTGCAGAAGAAGCGCCGGCTAACTACGTGCCAGCAGCCGCGGTAATACGTAGGGCGCAAGCGTT
GTCCGGAATTATTGGGCGTAAAGAGCTCGTAGGCGGCTTGTCGCGTCGGATGTGAAAGCCCGGGGCTT
AACCCCGGGTCTGCATTCGATACGGGCAGGCTAGAGTTCGGTAGGGGAGATCGGAATTCCTGGTGTAG
CGGTGAAATGCGCAGATATCAGGAGGAACACCGGTGGCGAAGGCGGATCTCTGGGCCGATACTGACG
CTGAGGAGCGAAAGCGTGGGGAGCGAACAGGATTAGATACCCTGGTAGTCCACGCCGTAAACGTTGG
GAACTAGGTGTGGGCGACATTCCACGTCGTCCGTGCCGCAGCTAACGCATTAAGTTCCCCGCCTGGGG
AGTACGGCCGCAAGGCTAAAACTCAAAGGAATTGACGGGGGCCCGCACAAGCAGCGGAGCATGTGGC
TTAATTCGACGCAACGCGAAGAACCTTACCAAGGCTTGACATACACCGGAAAGCATTAGAGATAGTGC
CCCCCTTGTGGTCGGTGTACAGGTGGTGCATGGCTGTCGTCAGCTCGTGTCGTGAGATGTTGGGTTAAG
TCCCGCAACGAGCGCAACCCTTGTTCTGTGTTGCCAGCATGCCCTTCGGGGTGATGGGGACTCACAGG
AGACTGCCGGGGTCAACTCGGAGGAAGGTGGGGACGACGTCAAGTCATCATGCCCCTTATGTCTTGGG
CTGCC--CGTGCTAC-
ATGGCCGGTAAAATGAGCTGCGATACCGCGAGGTGGAGCGAATCTCAAAAAGCCGGTCTCAGTTCGG
AATGGG

	Description	Scientific Name	Max Score	Total Score	Query Cover	E value	Per. Ident	Acc. Len	Accession
☐	Streptomyces huiliensis strain SCA2-4 16S ribosomal RNA, partial sequence	Streptomyces huiliensis	2244	2244	100%	0.0	99.43%	1523	NR_181624.1
☐	Streptomyces huiliensis strain SCA2-4 16S ribosomal RNA gene, partial sequence	Streptomyces huiliensis	2244	2244	100%	0.0	99.43%	1523	MW547058.1
☐	Streptomyces sp. strain KIB-H1992 16S ribosomal RNA gene, partial sequence	Streptomyces sp.	2230	2230	100%	0.0	99.35%	1373	MN180858.1
☐	Streptomyces sp. HBUM171191 16S ribosomal RNA gene, partial sequence	Streptomyces sp. HBUM 1...	2220	2220	100%	0.0	99.03%	1481	EU119195.1
☐	Streptomyces mobaraensis NBRC 13819 = DSM 40847 chromosome, complete genome	Streptomyces mobaraensi...	2206	15419	100%	0.0	98.87%	7574085	CP072827.1
☐	Streptomyces verticillus strain ATCC 15003 16S ribosomal RNA gene, partial sequence	Streptomyces verticillus	2206	2206	100%	0.0	98.87%	1398	KT377025.1
☐	Streptomyces mobaraensis strain NRRL B-3729 16S ribosomal RNA, partial sequence	Streptomyces mobaraensis	2200	2200	100%	0.0	98.79%	1539	NR_043830.1
☐	Streptomyces mobaraensis gene for 16S rRNA, partial sequence, strain: NBRC 13476	Streptomyces mobaraensis	2200	2200	100%	0.0	98.79%	1475	AB184430.1
☐	Streptomyces mobaraensis strain DRM1 16S ribosomal RNA gene, partial sequence	Streptomyces mobaraensis	2200	2200	100%	0.0	98.79%	1492	OR816118.1
☐	Streptomyces mobaraensis strain DSM 40587 chromosome, complete genome	Streptomyces mobaraensis	2200	15394	100%	0.0	98.79%	7638929	CP083590.1
☐	Streptomyces mobaraensis strain KKP 1387 16S ribosomal RNA gene, partial sequence	Streptomyces mobaraensis	2200	2200	100%	0.0	98.79%	1400	PP340959.1
☐	Streptomyces mobaraensis NBRC 13819 = DSM 40847 16S ribosomal RNA, partial sequence	Streptomyces mobaraensi...	2200	2200	100%	0.0	98.79%	1473	NR_112524.1
☐	Streptomyces mobaraensis strain DB13 16S ribosomal RNA gene, partial sequence	Streptomyces mobaraensis	2200	2200	100%	0.0	98.79%	1462	KX443339.1

Figure S1. 16S rRNA sequences of *Streptomyces* sp. S4S-00193A39, S4S-00200B03, CMB-MRB574 and CMB-GD066, and their BLAST search (closest match) on 16S rRNA sequences.

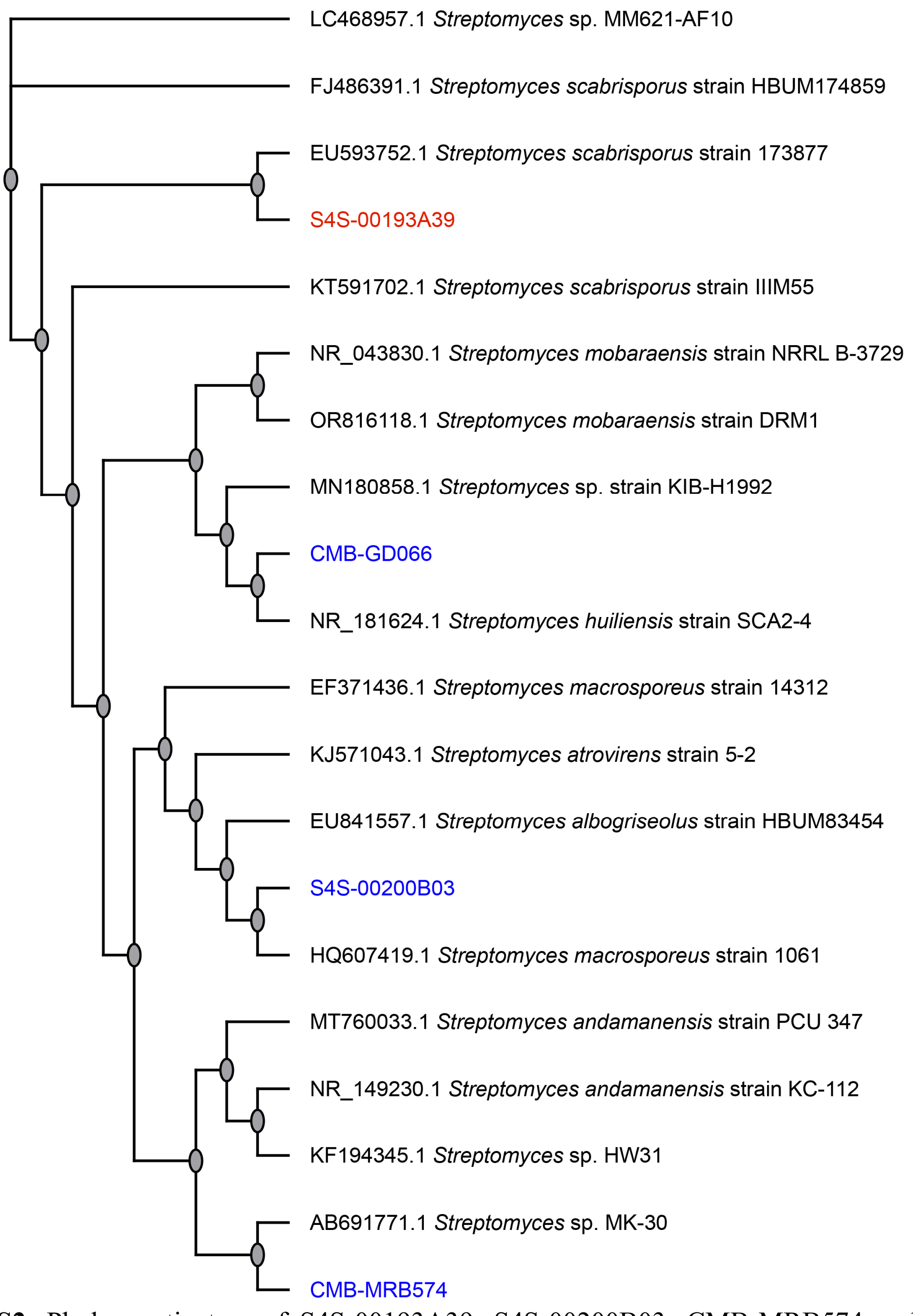

Figure S2. Phylogenetic tree of S4S-00193A39, S4S-00200B03, CMB-MRB574 and CMB-GD066.

Table S1. MATRIX media compositions.

Medium	Composition (per Litre)
**333**	Glucose (5.0 g), Peptone (3.0 g), Soluble Starch (10.0 g), Yeast extract (3.0 g), $CaCO_3$ (2.0 g), Agar (20.0 g)
**Glycerol Casein Agar (CGA)**	Glycerol (Chem-Supply) (30.0 g), Casein peptone (Amyl) (2.0 g), K_2HPO_4(Chem-Supply) (1.0 g), NaCl (Chem-Supply) (1.0 g), $MgSO_4.7H_2O$ (AnalaR) (0.5 g), Trace element solution (5.0 mL)*, Agar (Amyl) (20.0 g)<br><br>***Trace element solution:**<br>$CaCl_2.2H_2O$ (3.0 g), $FeC_6O_7H_5$ (1.0 g), $MnSO_4$ (0.2 g), $ZnCl_2$ (0.1 g), $CuSO_4.5H_2O$ (0.025 g), $Na_2B_4O_7.10H_2O$ (0.02 g), $CoCl_2$ (0.004 g), $Na_2MoO_4.2H_2O$ (0.01 g), Distilled H_2O (1 L) (Filter sterilize)
**D400**	Glucose (5.0 g), Malt extract (3.0 g), Peptone (3.0 g), Soluble Starch (20.0 g), Yeast extract (5.0 g), $CaCO_3$ (3.0 g), Agar (20.0 g)
**Glucose Yeast Extract Starch (GYA)**	Yeast extract (Difco) (4.0 g), Malt extract (Difco) (10.0 g), Glucose (Country Brewers) (4.0 g), $CaCO_3$ (Univar Ajax) (2.0 g), Soluble starch (Difco) (20.0 g), Agar (Amyl) (20.0 g), Adjust pH to 7.3
**ISP2 + Mannitol Agar (IMA)**	Yeast extract (Difco) (4.0 g), Malt extract (Difco) (10.0 g), Glucose (Country Brewers) (4.0 g), Mannitol (Amyl) (40.0 g), Agar (Amyl) (20.0 g), Adjust pH to 7.3
**ISP-2**	Yeast extract (Difco) (4.0 g), Malt extract (Difco) (10.0 g), Glucose (Country Brewers) (4.0 g), Agar (Amyl) (18.0 g), Adjust pH to 7.3
**M1**	Peptone (2.0 g), Yeast extract (4.0 g), Starch (10.0 g), Agar (18.0 g), pH 7.0
**M2**	Mannitol (40.0 g), Maltose (40.0 g), Yeast extract (10.0 g), K_2HPO_4 (2.0 g), $MgSO_4.7H_2O$ (0.5 g), $FeSO_4.7H_2O$ (0.01 g), Agar (20 g)
**SDA**	Peptic digest of animal tissue (5.0 g), Pancreatic digest of casein (5.0 g), Dextrose (40.0 g), Agar (15 g), pH 5.6
**YES**	Sucrose (150 g), Yeast extract (20 g), $MgSO_4.7H_2O$ (0.5 g), $ZnSO_4.7H_2O$ (0.01 g), $CuSO_4.5H_2O$ (0.005 g), Agar (20.0 g)
**Modified YEME media**	Yeast extract (Difco) (3.0 g), Bacto peptone (Difco) (5.0 g), Oxoin malt extract (3.0 g), Glucose (10.0 g), Sucrose (170.0 g), Agar (15.0 g)

Chemical profiling (UPLC-DAD and UPLC-QTOF)

UPLC-DAD chemical profiling involved injection of an aliquot of each analyte (2 µL at ~1 mg/mL in MeOH) through an Agilent 1290 infinity UPLC system (Zorbax SB-C_8 RRHD 1.8 µm, 2.1 × 50 mm column, eluting at 0.417 mL/min, 2.50 min gradient elution from 90% H_2O/MeCN to 100% MeCN with a constant 0.01% TFA modifier) equipped with a diode array multiple wavelength detector (DAD).

Aliquots (1 µL) of individual EtOAc extracts (100 µg/mL in MeOH) were analysed on UPLC-QTOF instrument comprising an Agilent 1290 Infinity II UPLC (Zorbax SB-C_8 RRHD 1.8 µm, 2.1×50 mm column, gradient elution at 0.417 mL/min over 2.50 min from 90% H_2O/MeCN to 100% MeCN with a constant 0.1% formic acid/MeCN modifier) coupled to an Agilent 6545 QTOF mass detector. MS/MS fragmentation for GNPS analysis was performed on the same machine for ions detected in the full scan range at an intensity above 200 counts at 5 scans/sec, with an isolation width of ~4 *m/z* using fixed collision energy and a maximum of 3 selected precursors per cycle. General instrument parameters included gas temperature at 325°C, drying gas at 10 L/min, nebulizer at 20 psi, sheath gas temperature at 400°C, fragmentation voltage at 180 V and skimmer at 45 V.

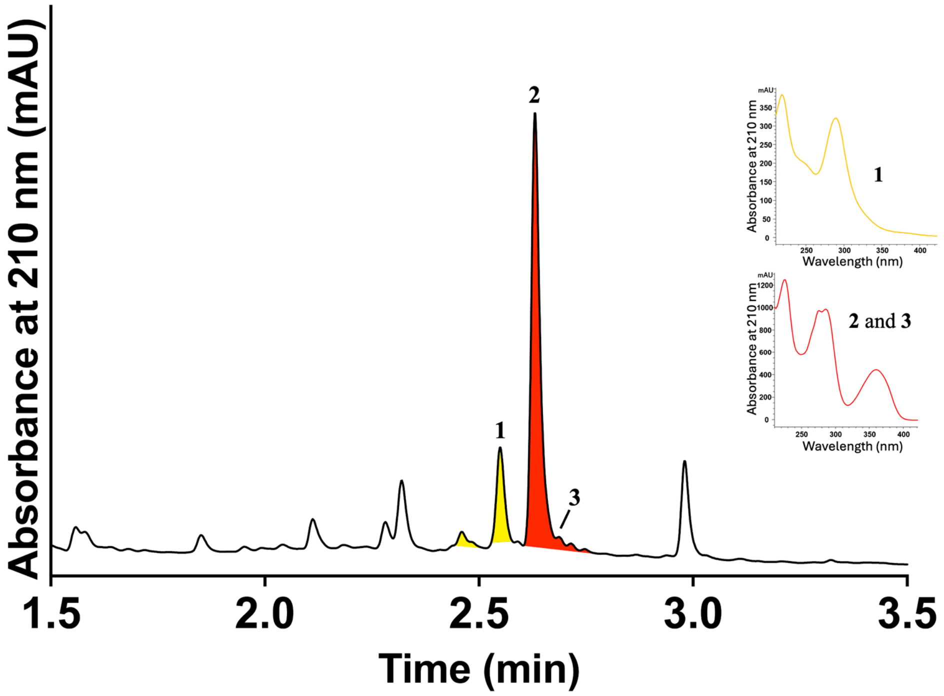

Figure S3. UPLC-DAD (210 nm) chromatogram of the EtOAc extract of an ISP2 cultivation of *Streptomyces* sp. S4S-00193A39.

Chemical Profiling (GNPS Molecular Networking)

Aliquots (1 µL) of individual EtOAc extracts (100 µg/mL in MeOH) were analysed on an Agilent 6545 UPLC-QTOF equipped with an Agilent 1290 Infinity II UPLC system (Zorbax SB-C_8 RRHD1.8 µm, 2.1 × 50 mm column, gradient elution at 0.417 mL/min over 2.5 min from 90% H_2O/MeCN to MeCN with an isocratic 0.1% formic acid/MeCN modifier).

UPLC-QTOF-(+) MS/MS data acquired for all samples at a collision energy of 35 eV were converted from Agilent MassHunter data files (.d) to an mzXML file format using MSConvert software and transferred to the GNPS server. Molecular networking was performed using the GNPS data analysis workflow employing the spectral clustering algorithm with a cosine score of 0.7 and a minimum of 6 matched peaks. The resulting spectral network was imported into Cytoscape version 3.8.0 and visualized using a ball–stick layout, where nodes represent parent mass, and cosine score was reflected by edge thickness. Also, group abundances were set as pie charts, which reflected the intensity of MS signals

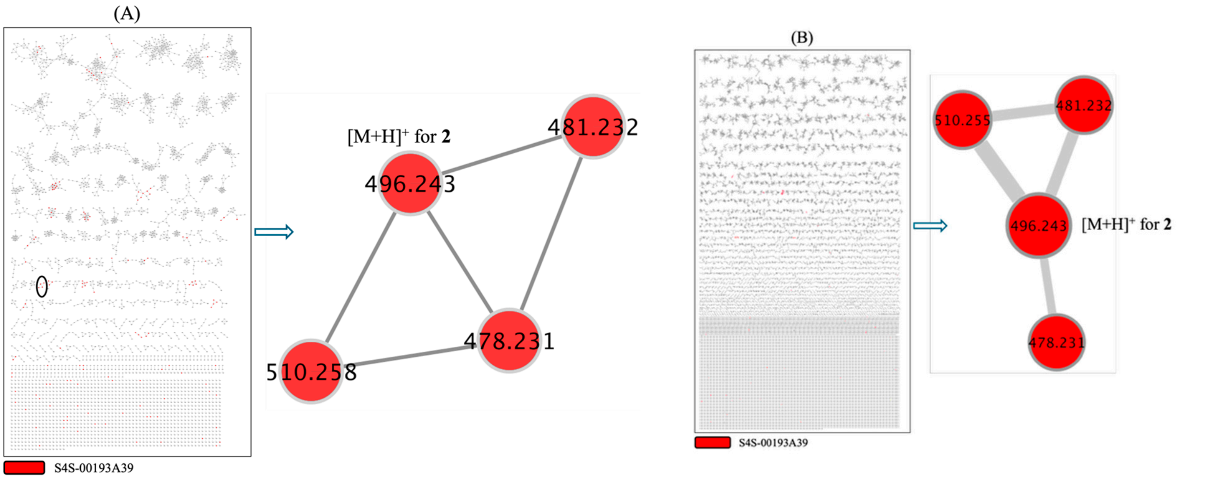

Figure S4. GNPS analysis of S4S-00193A39 with compounds related nodes. (A): GNPS molecular network for the library of ×704 isolates form ×19 Goondicum soil samples; (B): GNPS molecular network for an expanded Capon lab library of an additional ×1957 microbial extracts.

Cultivation profiling (MATRIX)

S4S-00193A39 was subjected to cultivation profiling in a 24-well plate (MATRIX) system using 11 different media compositions (Table S1) under solid agar (1.5 g), and static (1.5 mL) and shaken (1.5 mL, 190 rpm) broth formats at 27°C for 10 days. An additional set of control incubations were prepared from 11 different media solid phase cultivations without inoculation. Individual MATRIX wells were extracted in situ with EtOAc (2 mL) with the organic phases dried at 40°C under a stream of N_2, re-suspended in MeOH (100 µL). A portion of analyte was then subjected to GNPS chemical profiling (as described above), while a second portion was treated with an internal calibrant (Fmoc-Phe-OH, 20 µg/mL) and subjected to UPLC-DAD chemical profiling (as described above).

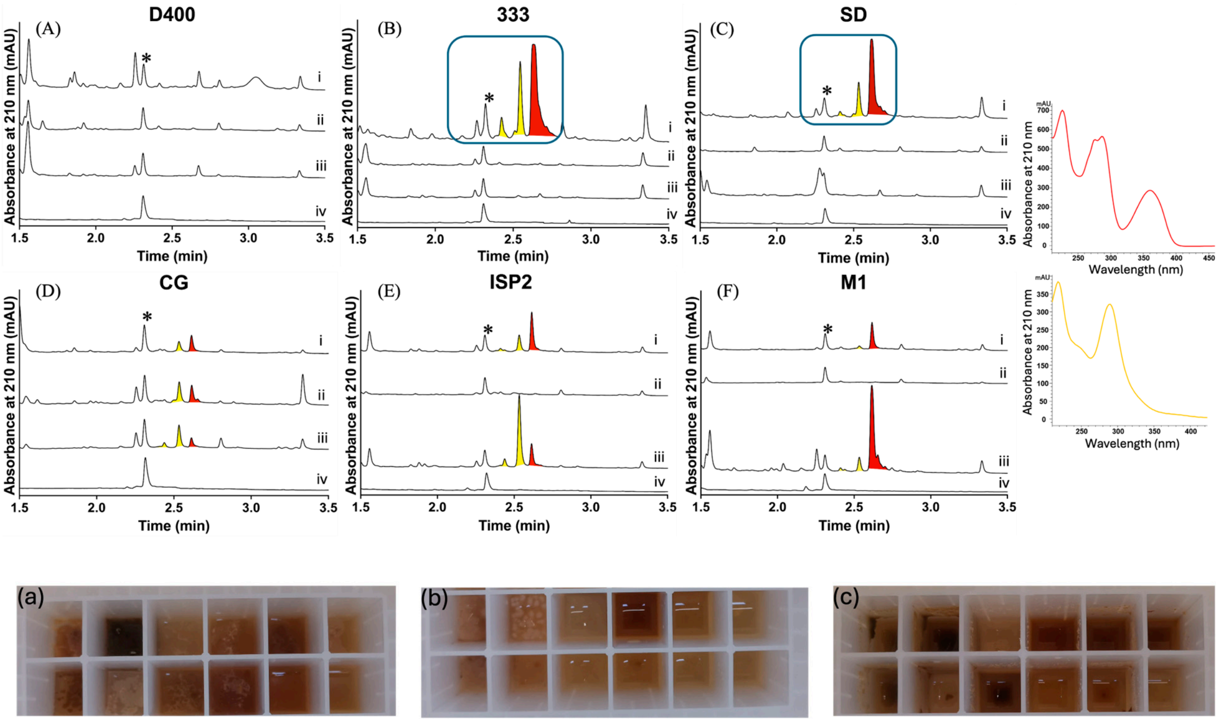

Figure S5. Top: UPLC-DAD chromatograms of a media composition study for S4S-00193A39 in (A) D400, (B) 333, (C) SD, (D) CG, (E) ISP2, (F) M1; (peaks painted by the identical colour with their UV-vis spectrums). i: agar; ii: static broth; iii: shaking broth; iv: media blank; *: calibrant: Fmoc-Phe-OH, 20 µg/mL. Bottom: image of MATRIX culture plates (a: agar, b: static broth, c: shaking broth).

Scale-up Cultivation and Fractionation of S4S-00193A39

A seed culture of S4S-00193A39 was prepared by inoculating 333 broth medium (70 mL) and shaking at 190 rpm at 30°C for 5 days. Aliquots of the seed culture (100 µL) were used to inoculate 333 agar plates (×100) and after incubation at 27°C for 14 days, the combined agar was extracted with EtOAc (2 × 500 mL) and the organic phase concentrated *in vacuo* to yield an extract (638.2 mg). This extract was then subjected to sequential trituration to afford after drying under nitrogen at 40°C *n*-hexane (84.1 mg), CH_2Cl_2 (319.5 mg) and MeOH (234.6 g) solubles, with the combined CH_2Cl_2 and MeOH solubles fractionated by preparative reverse-phase HPLC (Zorbax RX-C_8 7 µm, 21.2 × 250 mm column, 20 mL/min gradient elution over 20 min from 90% H_2O/MeCN to 100% MeCN, with a constant 0.01% TFA/MeCN modifier) to give 40 fractions. Fractions 23−24 were combined and subjected to semi-preparative reverse-phase HPLC (Zorbax SB-C_{18} 5 µm, 9.4×250 mm column, 3 mL/min gradient elution over 20 min at 60% MeCN/H_2O, with a constant 0.01% TFA/MeCN modifier) to give pure goondoxazole A (**1**) (2.3 mg, 0.36%) and goondoxazole B (**2**) (9.4 mg, 1.47%). Fraction 25 was subjected to semi-preparative reverse-phase HPLC (Zorbax SB-C_{18} 5 µm, 9.4×250 mm column, 3 mL/min gradient elution over 20 min at 65% MeCN/H_2O, with a constant 0.01% TFA/MeCN modifier) to give pure goondoxazole C (**3**) (1.5 mg, 0.24%).

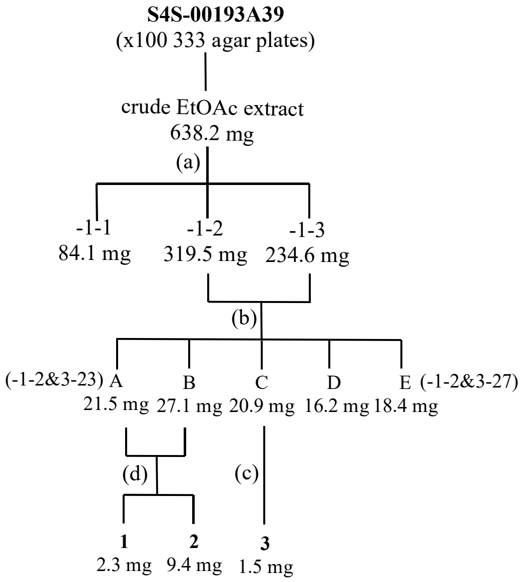

Figure S6. Isolation scheme for 333 agar medium cultivation of S4S-00193A39. (a) Trituration [Hexane (-1-1), DCM (-1-2) and MeOH (-1-3)], (b) Prep HPLC C_8, Zorbax RX-C_8, (c) Semi-prep HPLC C_{18}, Zorbax SB-C_{18}, (d) Semi-prep HPLC C_{18}, Zorbax SB-C_{18}.

Scale-up Cultivation and Fractionation of S4S-00200B03

A seed culture of S4S-00200B03 was used to inoculate ISP2 agar plates (×20) which were incubated at 27°C for 14 days, after which the combined agar was extracted with EtOAc (2 × 500 mL) and the organic phase concentrated *in vacuo* to yield an extract (69.3 mg). This extract was subjected to sequential trituration to afford after drying under nitrogen at 40°C *n*-hexane (12.8 mg), CH_2Cl_2 (38.67 mg) and MeOH (11.51 mg) solubles. The combined CH_2Cl_2 and MeOH solubles were fractionated by preparative reverse-phase HPLC (PrepHT Phenomenex C_8 5 µm, 10 × 250 mm column, 3 mL/min gradient elution over 20 min at 30% H_2O/MeCN, with a constant 0.01% TFA/MeCN modifier) to give A-33853 (**12**) (0.22 mg, 0.32%).

Scale-up Cultivation and Fractionation of CMB-MRB574

A seed culture of CMB-MRB574 was used to inoculate ISP2 agar plates (×150) which were incubated at 27°C for 14 days, after which the combined agar was extracted with EtOAc (2 × 500 mL) and the organic phase concentrated *in vacuo* to yield an extract (590.8 mg). This extract was subjected to sequential trituration to afford after drying under nitrogen at 40 °C *n*-hexane (65.2 mg), CH_2Cl_2 (320.3 mg) and MeOH (163.6 mg) solubles. The CH_2Cl_2 solubles were fractionated by preparative reverse-phase HPLC (PrepHT Phenomenex C_8 5 µm, 10 × 250 mm column, 3 mL/min gradient elution over 20 min at 30% H_2O/MeCN, with a constant 0.01% TFA/MeCN modifier) to give UK-1 (**13**) (0.8 mg, 0.14%).

Scale-up Cultivation and Fractionation of CMB-GD066

A seed culture of CMB-GD066 was used to inoculate ISP2 agar plates (×50) which were incubated at 27 °C for 14 days, after which the combined agar was extracted with EtOAc (2 × 500 mL) and the organic phase concentrated *in vacuo* to yield an extract (240.5 mg). This extract was subjected to sequential trituration to afford after drying under nitrogen at 40 °C *n*-hexane (6.7 mg), CH_2Cl_2 (211.4 mg) and MeOH (6.7 mg) solubles. The CH_2Cl_2 solubles were fractionated by preparative reverse-phase HPLC (as described above) to give pure nataxazole (**14**) (1.0 mg, 0.42%) and 5-hydroxynataxazole (**15**) (1.0 mg, 0.42%).

Spectroscopic characterisation of goondoxazole A (1)

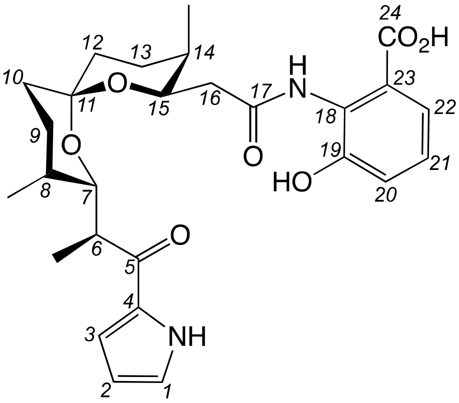

Table S2. 1D and 2D NMR (DMSO-d_6) data for goondoxazole A (**1**).

**Pos.**	δ_H, mult (*J* in Hz)	δ_C, type	COSY	^{1}H-^{13}C HMBC	ROESY
1	7.03, br s	125.2, CH	2, 1-NH	2, 3, 4	2, 1-NH
2	6.14, ddd (*3.7*, *2.3*, *2.2*)	109.5, CH	1, 3, 1-NH	1, 3, 4	1, 3
3	6.90, br s	116.4, CH	2, 1-NH	2, 4	2, 6
4	-	132.6, C			
5	-	192.7, C			
6	3.20, dq (*10.3*, *6.9*)	42.0, CH	7, 6-Me	5, 7, 6-Me	3, 6-Me, 8-Me
7	3.98, dd (*10.3*, *2.2*)	72.4, CH	6, 8	5, 6, 9, 11, 6-Me, 8-Me	8, 9a, 15, 6-Me, 19-OH
8	1.65, m	26.5, CH	7, 8-Me		9a, 9b, 7, 6-Me
9	*a*. 2.01, dddd (*14.5*, *13.5*, *5.3*, *4.4*)	25.6, CH_2	9b, 10a	8-Me	7, 8, 9b
	b. 1.28^A, m		9a	7, 11	8, 9a
10	a 1.45, ddd (*14.7*, *13.7*, *4.4*)	29.4, CH_2	9a, 10b	9, 11, 12	10b, 8-Me
	b 1.31^A, m		10a	9, 11, 12	10a, 8-Me
11	-	95.5, C			
12	*a*. 1.38^B, m	29.1, CH_2	12b		12b
	b. 1.13, m		12a, 13b	11, 14	12a
13	*a*. 1.38^B, m	25.3, CH_2	13b	14-Me	13b
	b. 1.04, m		12b, 13a	11, 14, 15, 14-Me	13a, 14
14	1.53, m	29.0, CH	15, 14-Me	14-Me	13b, 15, 16b
15	4.14, ddd (*8.9*, *5.3*, *2.4*)	67.4, CH	14, 16	16, 17, 14-Me	7, 14, 16a, 16b
16	*a*. 2.44, dd (*14.4*, *8.9*)	40.9, CH_2	15	14, 15, 17	15, 14-Me
	b. 2.35, dd (*14.4*, *5.3*)		15	14, 15, 17	15, 14-Me
17	-	170.6, C			
18	-	126.0, C			
19	-	150.8, C			
20	7.09, d (*8.0*)	121.2, CH	21	18, 22	22, 19-OH
21	7.13, dd (*8.0*, *7.7*)	125.7, CH	20, 22	19, 23	22
22	7.35, dd (*8.0*, *1.5*)	121.4, CH	21	18, 20, 24	20, 21
23	-	126.3, C			
24	-	168.5, C			
6-Me	0.83^C, br d (*6.9*)	13.4, CH_3	6	5, 6, 7	6, 7, 8
8-Me	0.92, d (*6.9*)	10.8, CH_3	8	7, 8, 9	6, 10a, 10b
14-Me	0.83^C, br d (*6.9*)	11.1, CH_3	14	13, 14, 15	16a, 16b
19-OH	9.70, br s	-		18$^\#$, 21$^\#$, 23$^\#$	7, 20
1-NH	11.71, br s	-	1, 2, 3	1$^\#$, 2, 3, 4$^\#$	1
17-NH	-	-			

$^{A-C}$ - resonance with the same superscript overlap, $^\#$ weak signals.

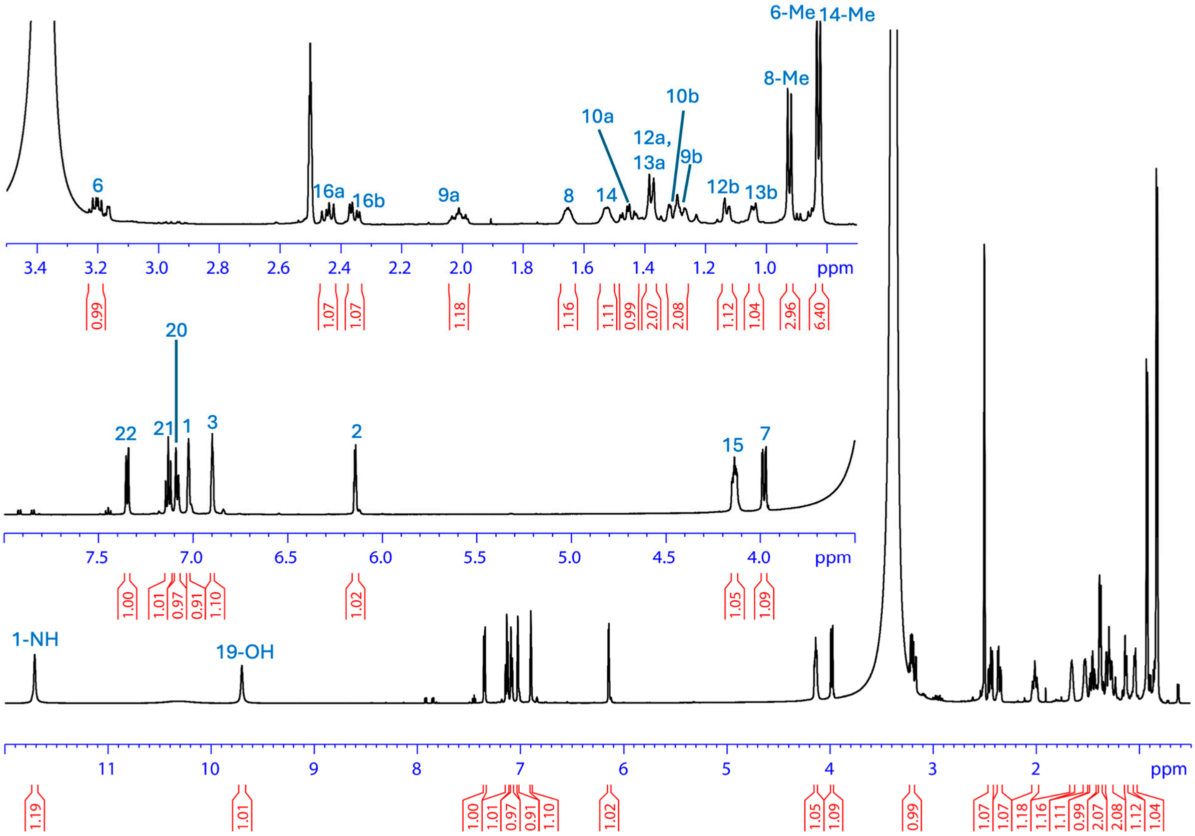

Figure S7. ^{1}H NMR (600 MHz, DMSO-d_6) spectrum of goondoxazole A (**1**).

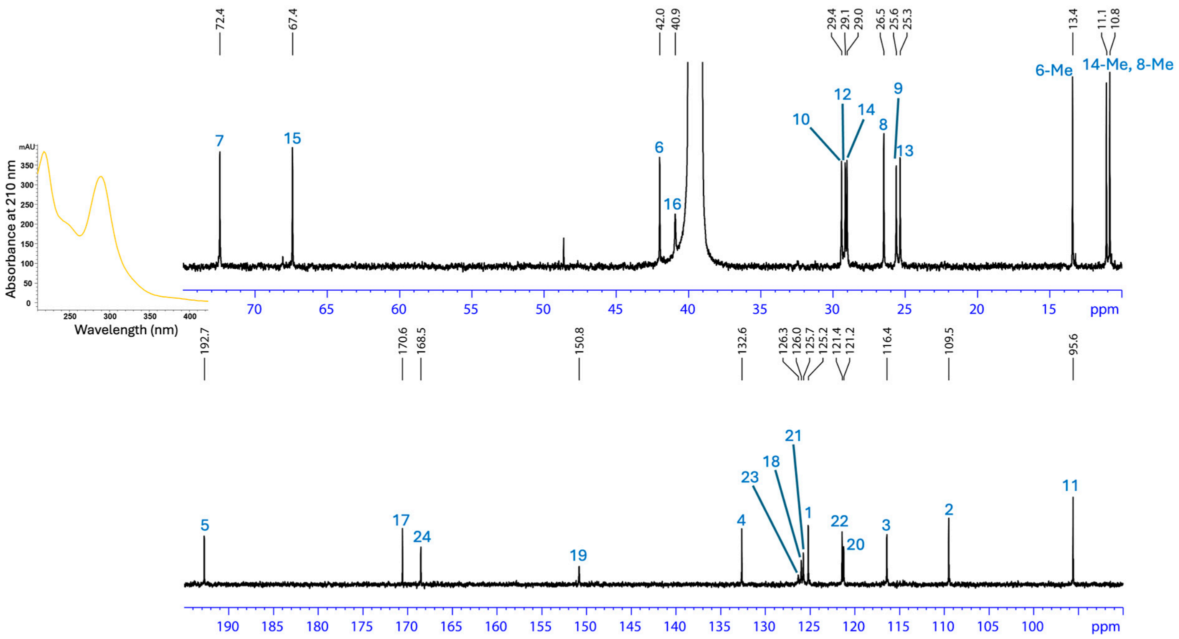

Figure S8. ^{13}C NMR (150 MHz, DMSO-d_6) and UV-vis (inset) spectra of goondoxazole A (**1**).

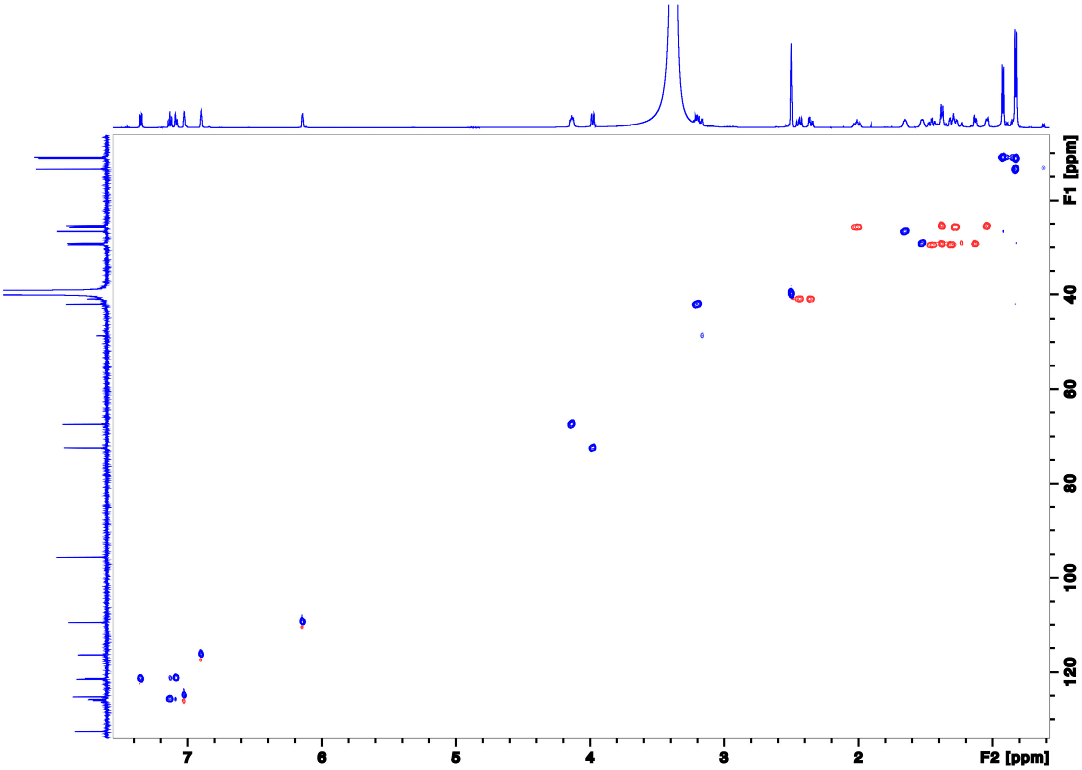

Figure S9. HSQC NMR (DMSO-d_6) spectrum of goondoxazole A (**1**).

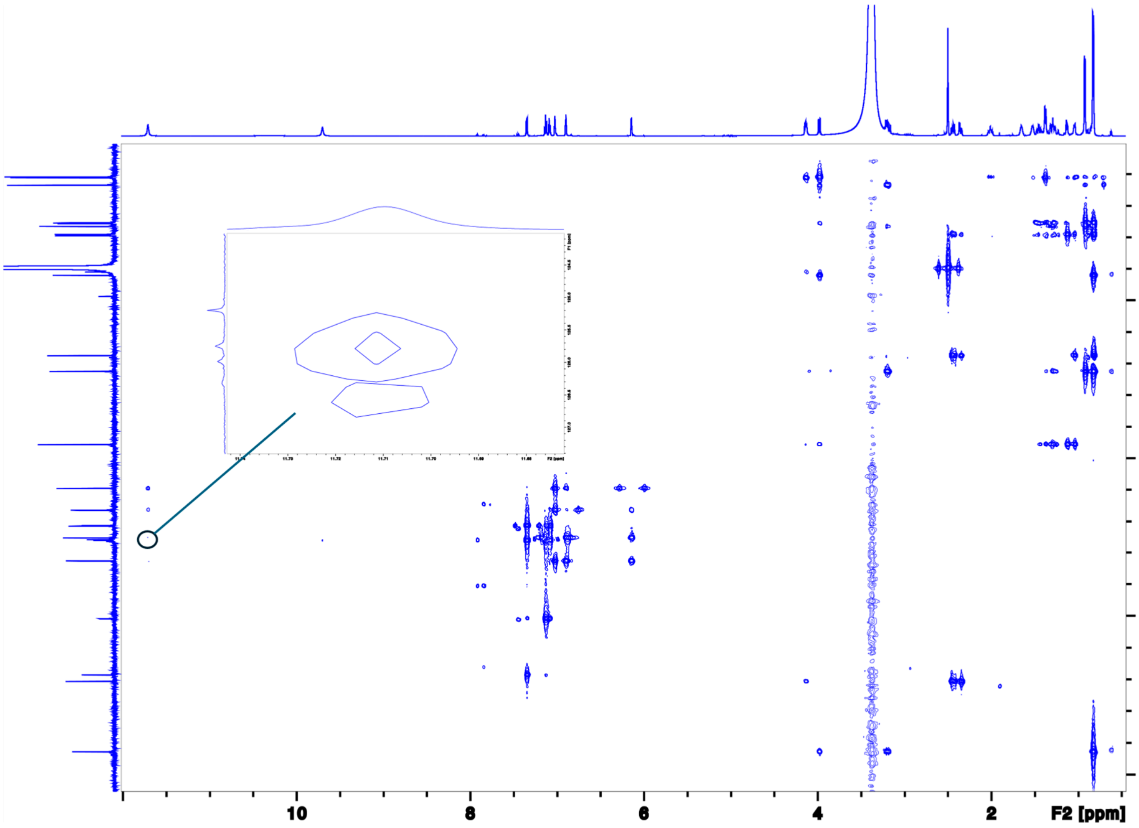

Figure S10. HMBC NMR (DMSO-d_6) spectrum of goondoxazole A (**1**).

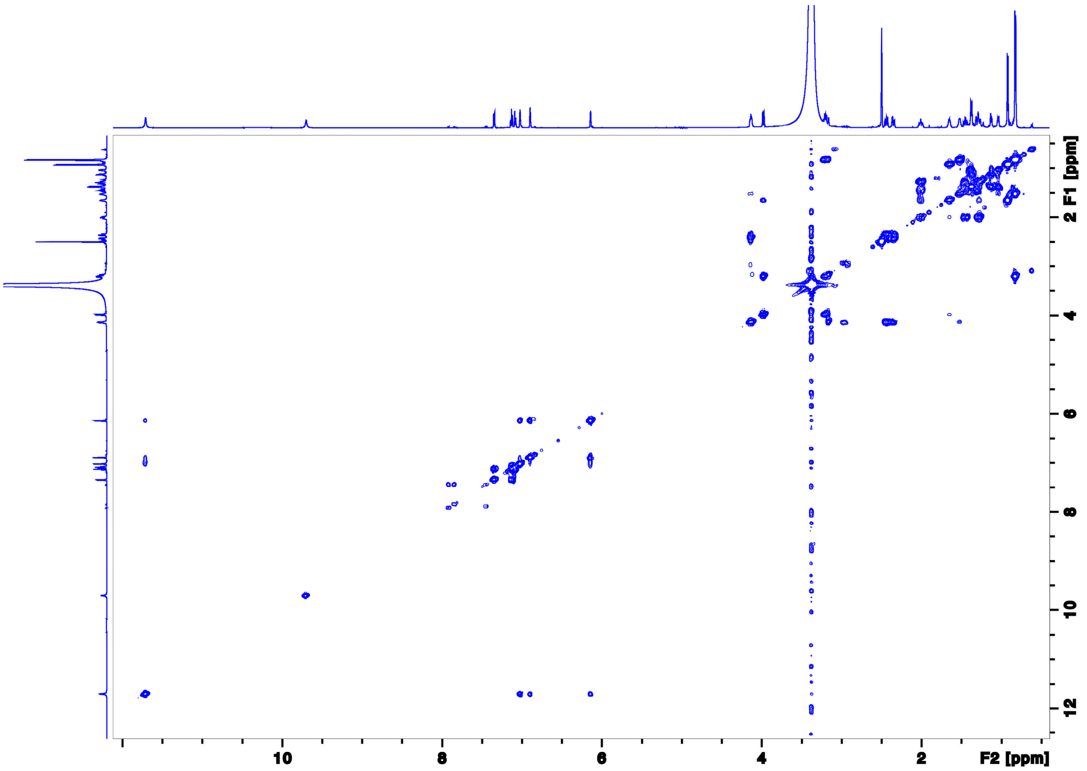

Figure S11. COSY NMR (DMSO-d_6) spectrum of goondoxazole A (**1**).

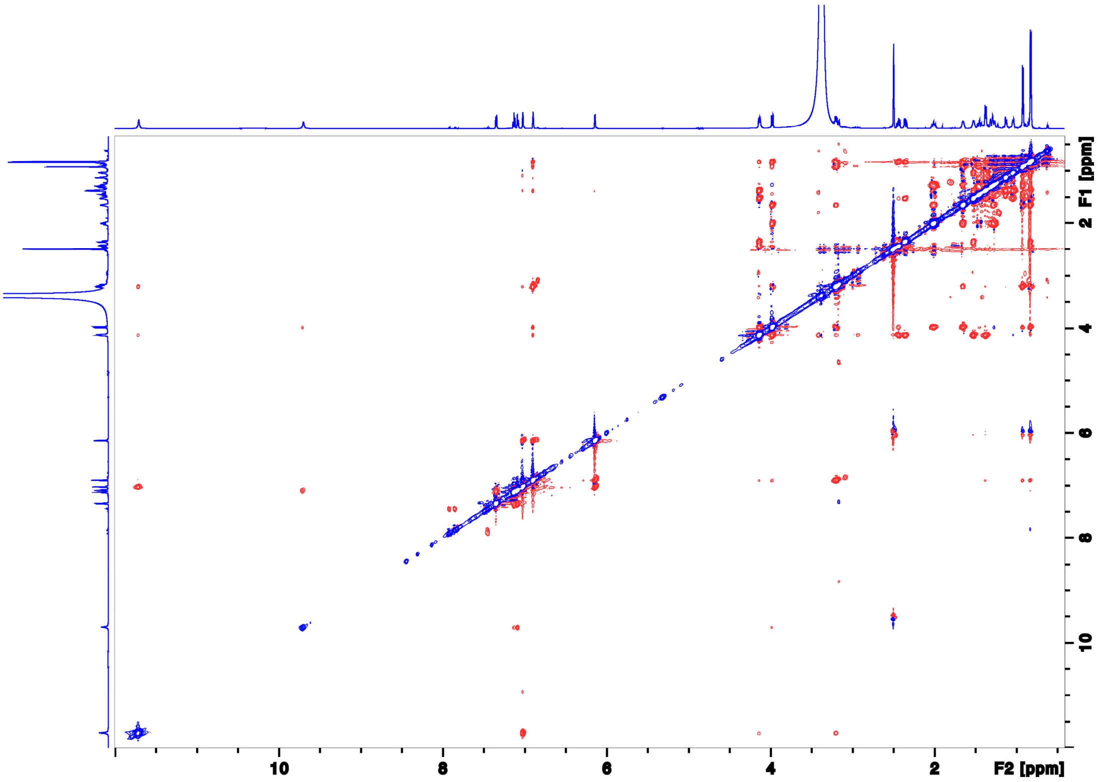

Figure S12. ROESY NMR (DMSO-d_6) spectrum of goondoxazole A (**1**).

Table S3. 1D NMR ($CDCl_3$) comparison of goondoxazole A (**1**) and X-14885A (**4**).

**Pos.**	**(1)** δ_C	**(1)** δ_H, mult (*J* in Hz)	**(4)**[#] δ_C	**(4)**[#] δ_H
1	126.3	7.04, br s	124.4	7.07
2	110.8	6.25, m	110.3	6.26
3	118.9	6.99, br s	117.5	6.93
4	133.1	-	133.1	-
5	195.7	-	194.0	-
6	43.1	3.22, dq (*10.2*, *6.9*)	42.6	3.23
7	74.0	3.92, br d (*10.2*)	73.3	3.62
8	27.1	1.77, m	27.0	1.65
9	26.2	*a*. 2.06, dddd (*14.7*, *13.8*, *5.1*, *4.3*)	29.5	*a*. 1.95
		b. 1.38[A], m		*b*. 1.40
10	30.0	*a*. 1.52, ddd (*14.8*, *13.5*, *4.3*)	26.1	*a*. 1.60
		b. 1.40[A], m		*b*. 1.44
11	96.6	-	96.5	-
12	29.8	*a*. 1.44[A], m	29.6	*a*. 1.51
		b. 1.21, br d (*12.6*)		*b*. 1.17
13	25.8	*a*. 1.28, m	25.7	*a*. 1.49
		b. 1.06, br d (*12.6*)		*b*. 1.30
14	29.6	1.46[A], m	29.3	1.58
15	69.1	3.98, m	68.8	4.30
16	42.1	*a*. 2.66, dd (*13.2*, *8.8*)	32.7	*a*. 3.17
		b. 2.50, dd (*13.2*, *6.4*)		*b*. 3.05
17	172.6	-	168.1	-
18	121.8	-	143.2	-
19	150.4	-	140.2	-
20	125.5	7.21, br d (*7.6*)	114.7	7.70
21	126.3	7.14, dd (*7.6*, *7.5*)	116.5	7.01
22	123.5	7.59, d (*7.5*)	159.7	-
23	128.0	-	101.8	-
24	170.3	-	169.5	-
6-Me	13.0	0.87, d (*6.0*)	13.2	0.96
8-Me	10.8	0.96, d (*6.9*)	10.6	0.99
14-Me	11.1	0.88, d (*6.3*)	10.9	0.96
19-OH	-	10.73, br s		
1-NH	-	10.43, br s		

[A] - resonance with the same superscript overlap, [#] J. Am. Chem. Soc., 1989, 111, 8598-8609.

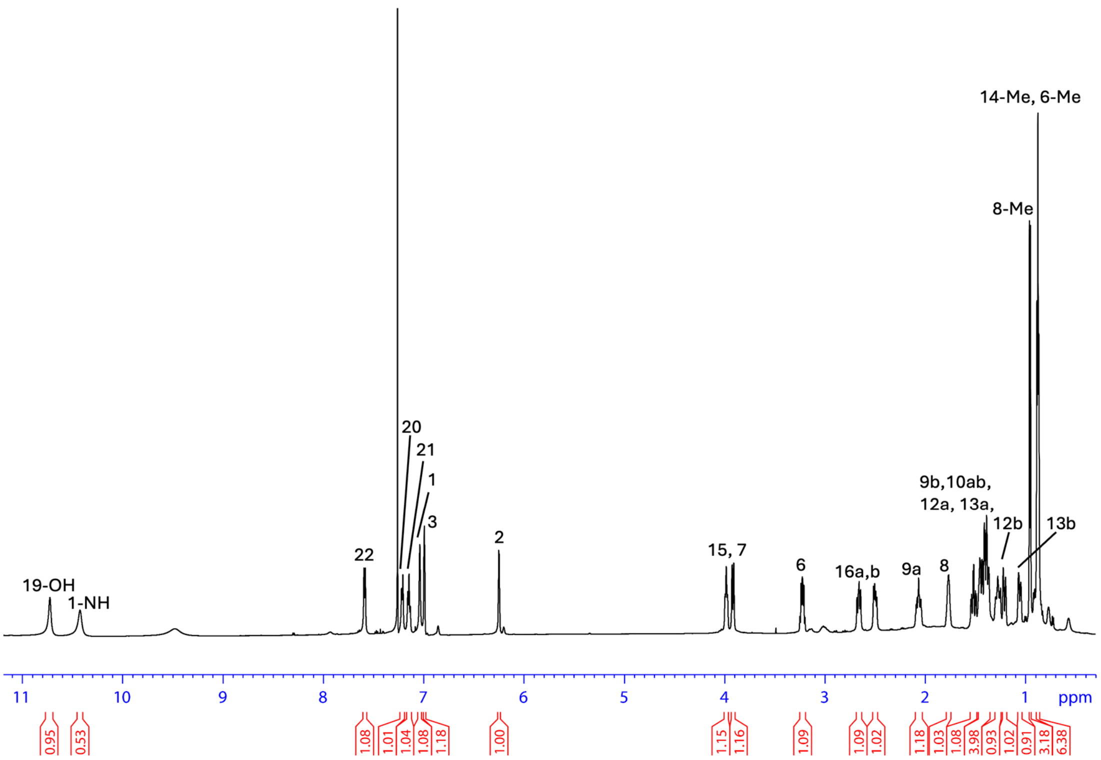

Figure S13. ^{1}H NMR (600 MHz, $CDCl_3$) spectrum of goondoxazole A (**1**).

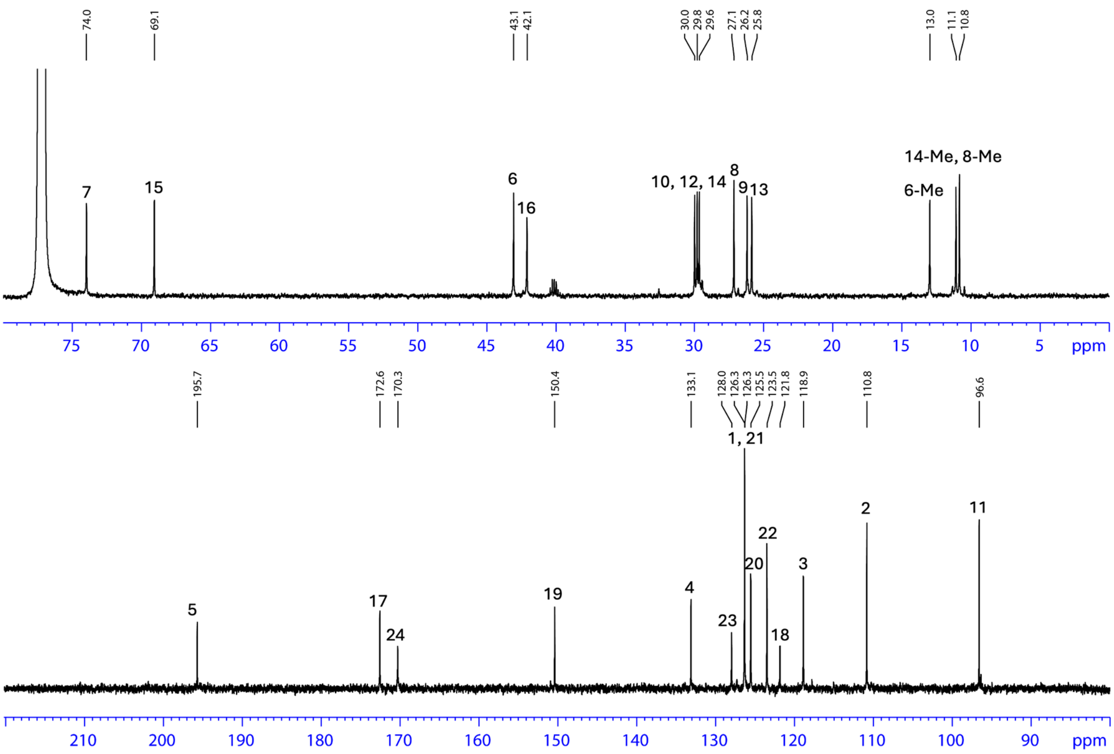

Figure S14. ^{13}C NMR (150 MHz, $CDCl_3$) spectrum of goondoxazole A (**1**).

Mass Spectrum Molecular Formula Report

Analysis Info

Analysis Name	D:\Data\Shengbin\S4S193A39-498.d	Acquisition Date	10/25/2023 4:13:38 PM
Method	tune-med_AP.m	Operator	a.salim
Sample Name	S4S193A39-498	Instrument / Ser#	micrOTOF 213750.00232
Comment			

Acquisition Parameter

Source Type	ESI	Ion Polarity	Positive	Set Nebulizer	0.8 Bar
Focus	Not active			Set Dry Heater	180 °C
Scan Begin	100 m/z	Set Capillary	4500 V	Set Dry Gas	5.0 l/min
Scan End	1000 m/z	Set End Plate Offset	-500 V	Set Divert Valve	Source

Generate Molecular Formula Parameter

Formula, min.		
Formula, max.		
Measured m/z	Tolerance	Charge
Check Valence	Minimum	Maximum
Nirogen Rule	Electron Configuration	
Filter H/C Ratio	Minimum	Maximum
Estimate Carbon		

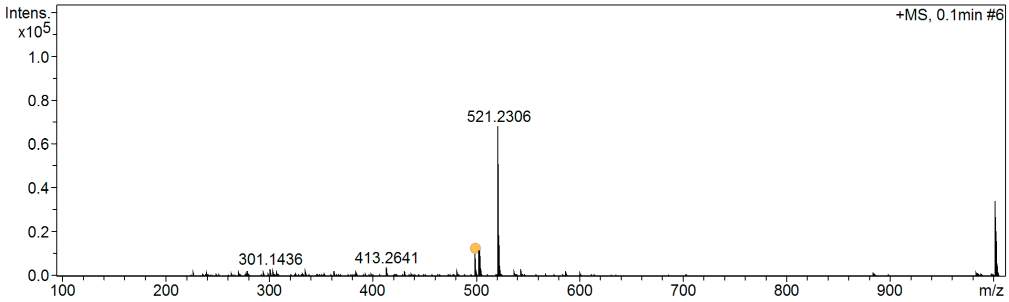

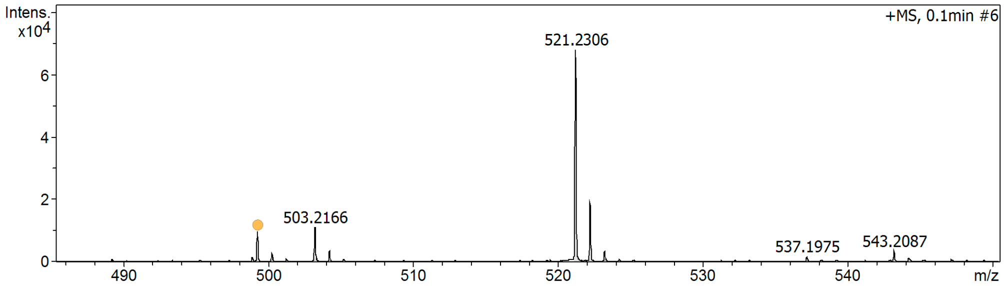

Meas. m/z	#	Ion Formula	m/z	err [ppm]	mSigma	# Sigma	Score	rdb	e⁻ Conf	N-Rule
499.2459	1	C27H35N2O7	499.2439	4.1	18.5	1	51.56	11.5	even	ok

Bruker Compass DataAnalysis 4.1 printed: 10/25/2023 4:19:48 PM Page 1 of 1

Figure S15. HRESIMS spectrum for goondoxazole A (**1**).

Spectroscopic characterisation of goondoxazole B (2)

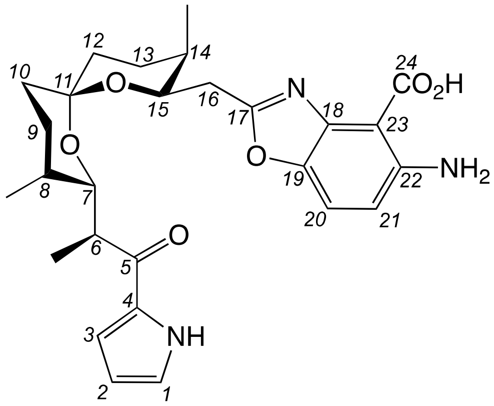

Table S4. 1D and 2D NMR (DMSO-d_6) data for goondoxazole B (**2**).

Pos.	δ_H, mult (*J* in Hz)	δ_C, type	COSY	^1H-^{13}C HMBC	ROESY
1	7.01, br s	125.0, CH	2, 1-NH	3, 4	2, 1-NH
2	6.12, ddd (*3.6*, *2.4*, *2.3*)	109.4, CH	1, 3, 1-NH	1, 3, 4	1, 3
3	6.84, br s	116.2, CH	2, 1-NH	1, 3, 4	2, 6
4	-	132.6, C			
5	-	192.1, C			
6	3.09, dq (*10.3*, *6.9*)	41.6, CH	7, 6-Me	5, 7, 6-Me	3, 6-Me, 8-Me
7	3.34*, m	72.3, CH	6	6, 6-Me, 8-Me	7, 8, 9a, 15, 6-Me
8	1.40^A, m	26.4, CH	8-Me	9	7, 6-Me, 8-Me
9	*a*. 1.74, dddd (*13.8*, *13.3*, *5.4*, *4.0*)	25.5, CH_2	10a, 9b	8-Me	7, 9b
	b. 1.22^B, m		9a	7, 11	9a
10	*a*. 1.41^A, m	29.2, CH_2	9a, 10b	9, 11, 12	10b
	b. 1.26^B, m		10a	9, 12	10a
11	-	95.5, C			
12	*a*. 1.37^C, m	28.9, CH_2	12b	11, 14	12b
	b. 1.11^D, m		12a	11, 14	12a
13	*a*. 1.36^C, m	25.3, CH_2	13b	14, 14-Me	13b, 15
	b. 1.07^D, m		13a	14, 15, 14-Me	13a
14	1.54, m	29.5, CH	15, 14-Me		15, 16b, 14-Me
15	4.13, ddd (*9.6*, *4.0*, *3.2*)	68.5, CH	14, 16	16, 17, 14-Me	7, 13a, 14
16	*a*. 2.94, dd (*14.6*, *9.6*)	32.4, CH_2	15	14, 15, 17	14-Me
	b. 2.90, dd (*14.6*, *4.0*)		15	14, 15, 17	14, 4-Me
17	-	166.1, C			
18	-	140.5, C			
19	-	140.9, C			
20	7.66, d (*9.0*)	116.8, CH	21	18, 22, $23^\#$	
21	6.80, d (*9.0*)	114.1, CH	20	19, 23, $24^\#$	
22	-	150.0, C			
23	-	97.8, C			
24	-	167.4, C			
6-Me	0.64, d (*6.9*)	13.2, CH_3	6	5, 6, 7	6, 7, 8
8-Me	0.85, d (*6.9*)	10.7, CH_3	8	7, 8, 9	6, 8
14-Me	0.88, d (*6.9*)	10.9, CH_3	14	13, 14, 15	14, 16a, 16b
1-NH	11.76, br s	-	1, 2, 3	2, 3, 4	1

$^{A-D}$ - resonance with the same superscript overlap, $^\#$ weak signals, * obscured by solvent.

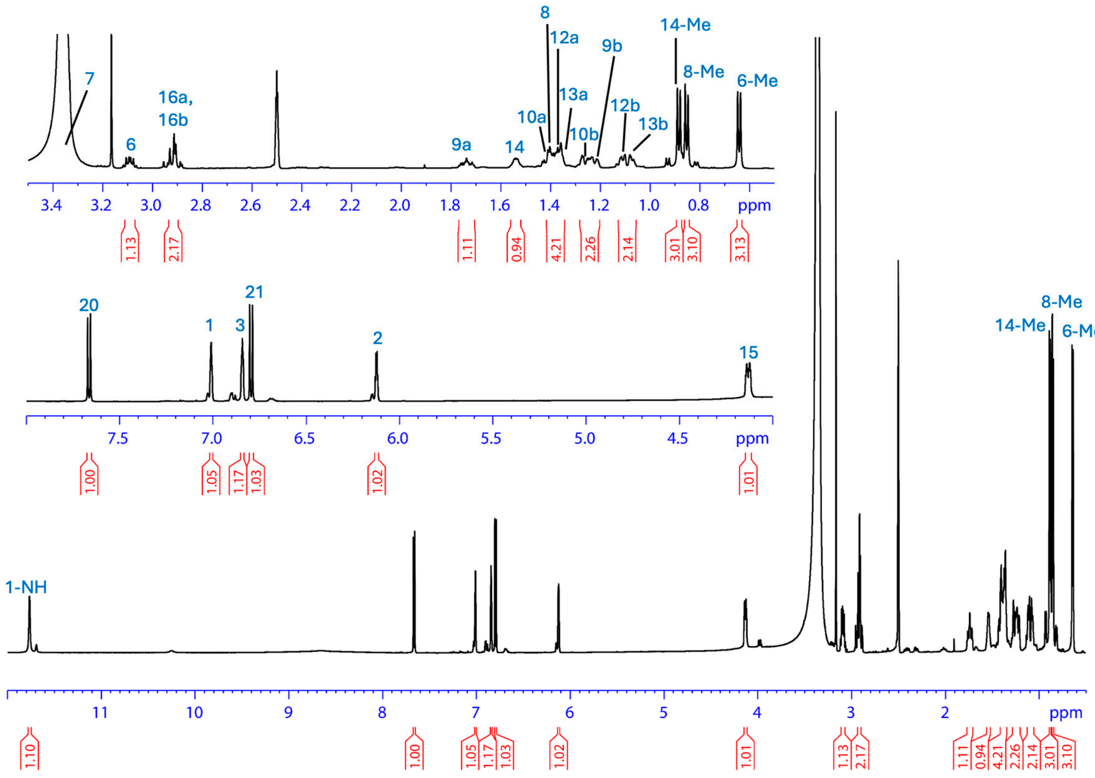

Figure S16. ^{1}H NMR (600 MHz, DMSO-d_6) spectrum of goondoxazole B (**2**).

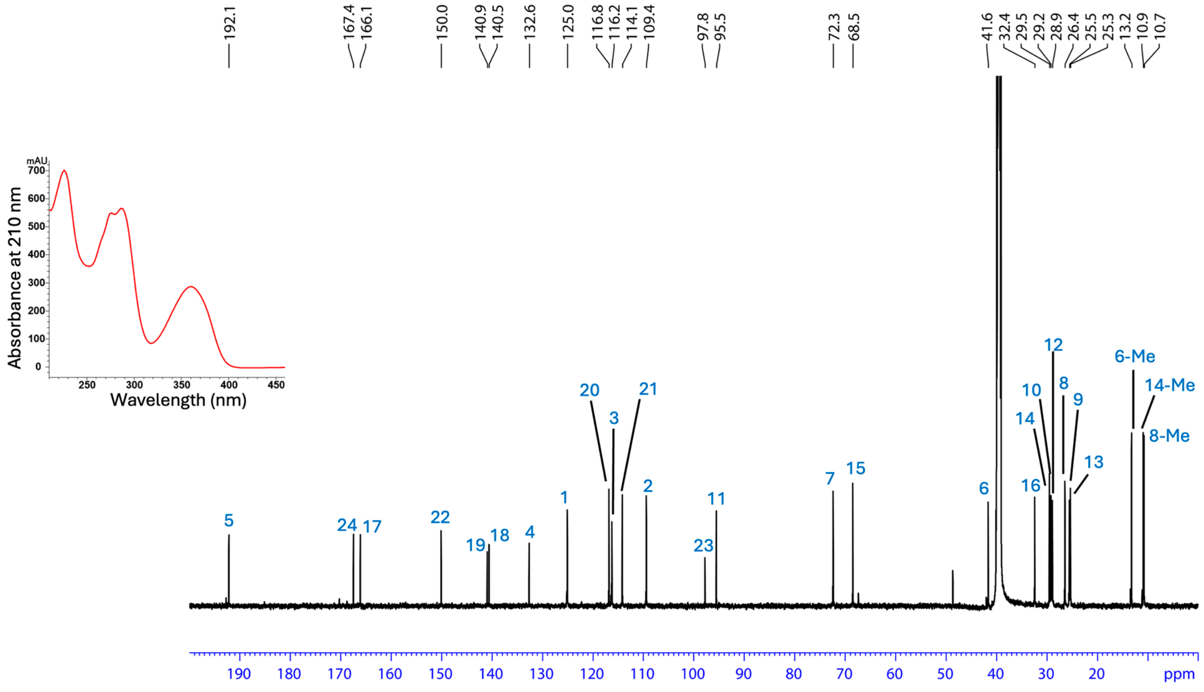

Figure S17. ^{13}C NMR (150 MHz, DMSO-d_6) and UV-vis (inset) spectra of goondoxazole B (**2**).

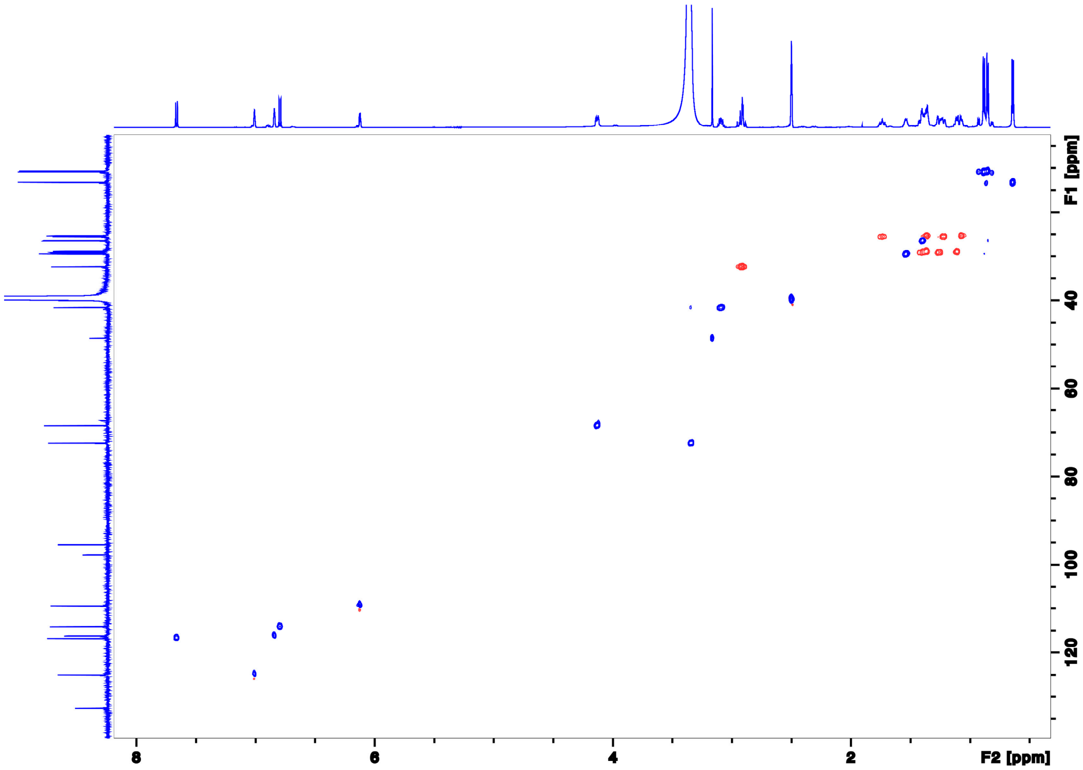

Figure S18. HSQC NMR (DMSO-d_6) spectrum of goondoxazole B (**2**).

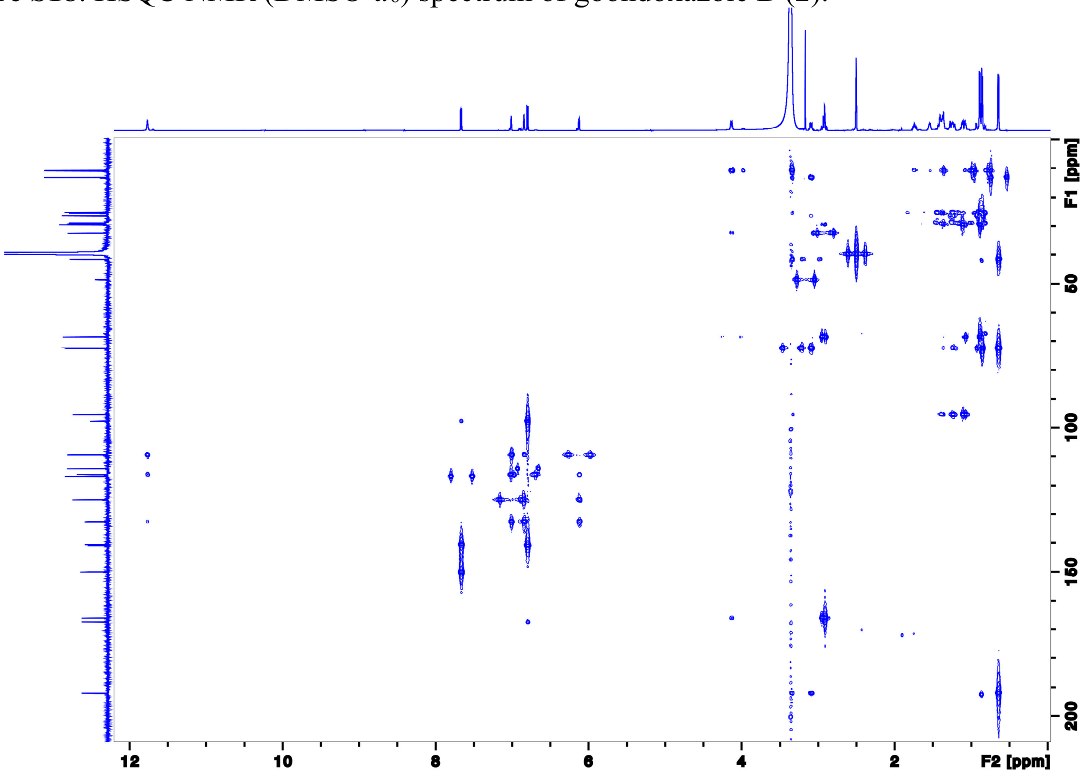

Figure S19. HMBC NMR (DMSO-d_6) spectrum of goondoxazole B (**2**).

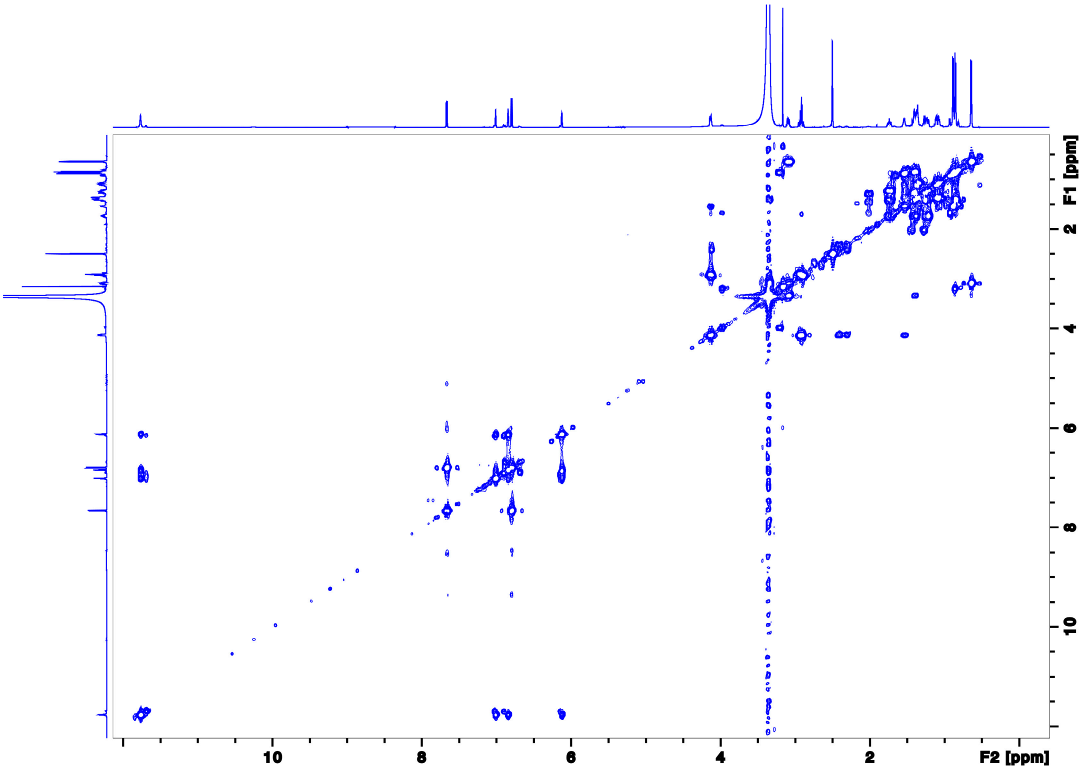

Figure S20. COSY NMR (DMSO-d_6) spectrum of goondoxazole B (**2**).

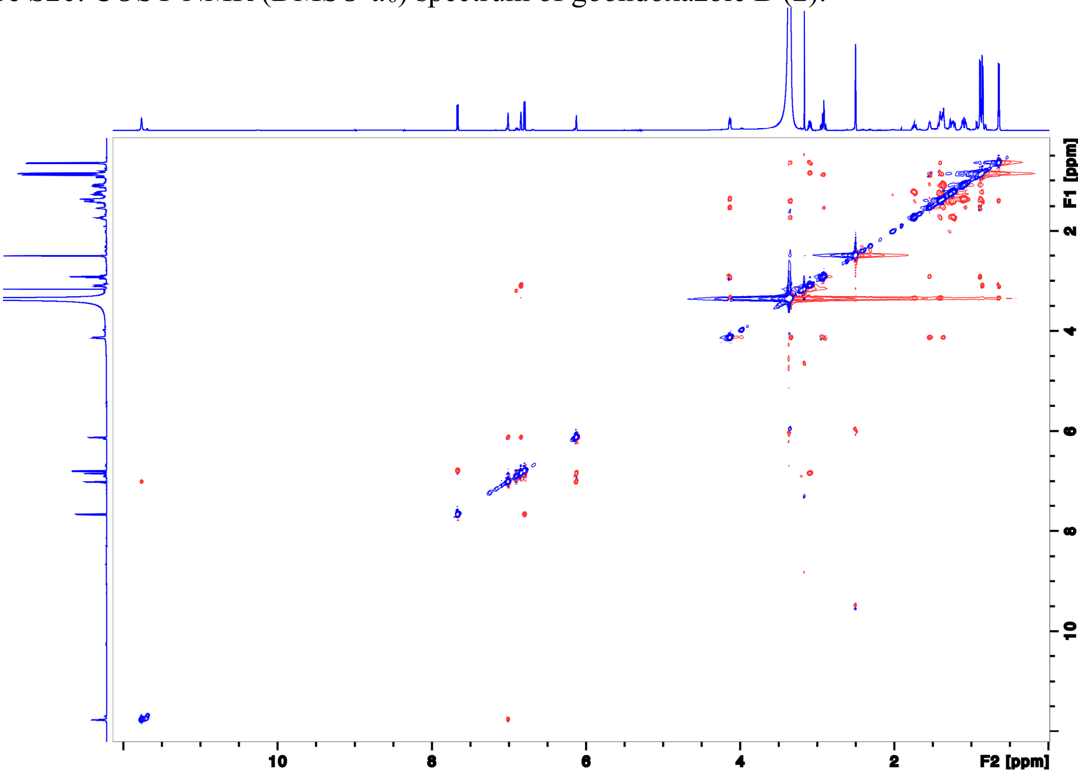

Figure S21. ROESY NMR (DMSO-d_6) spectrum of goondoxazole B (**2**).

Table S5. ^{13}C NMR ($CDCl_3$) comparison of goondoxazole B (**2**) and calcimycin (**5**).

Pos.	**(2)** δ_H, mult (*J* in Hz)	**(2)** δ_C	**(5)**[#] δ_C
1	7.06, br s	124.8	124.3
2	6.24, br s	110.2	110.1
3	6.91, br s	116.6	116.3
4	-	133.2	133.0
5	-	194.1	193.7
6	3.18, m	42.6	42.5
7	-	73.4	72.9
8	1.60, m	27.0	28.3
9	*a*. 1.87, m<br>*b*. 1.36^A, m	26.2	35.2
10	*a*. 1.54^B, m<br>*b*. 1.40^A, m	29.6	32.3
11	-	96.6	98.5
12	*a*. 1.45^A, m<br>*b*. 1.23, br d (*10.5*)	29.6	25.4
13	*a*. 1.43^A, m<br>*b*. 1.09, br d (*12.1*)	25.7	25.7
14	1.50^B, br s	28.9	28.8
15	-	69.0	68.4
16	*a*. 3.07, m<br>*b*. 2.98, dd (*15.3*, *6.6*)	32.4	32.4
17	-	166.5	166.1
18	-	141.4	140.8
19	-	141.9	141.7
20	7.46, br s	116.6	116.7
21	6.62, br d (*7.5*)	114.4	108.4
22	-	149.2	150.8
23	-	96.5	98.2
24	-	167.9	168.1
6-Me	0.89, br s	13.2	13.0
8-Me	0.93, br d (*5.8*)	10.7	10.7
10-Me	-	-	16.1
14-Me	0.90, br s	11.0	11.3
22-NHMe	-	-	30.0

$^{A-B}$ - resonance with the same superscript overlap, [#] J. Antibiot., 1982, 35, 1409-1411

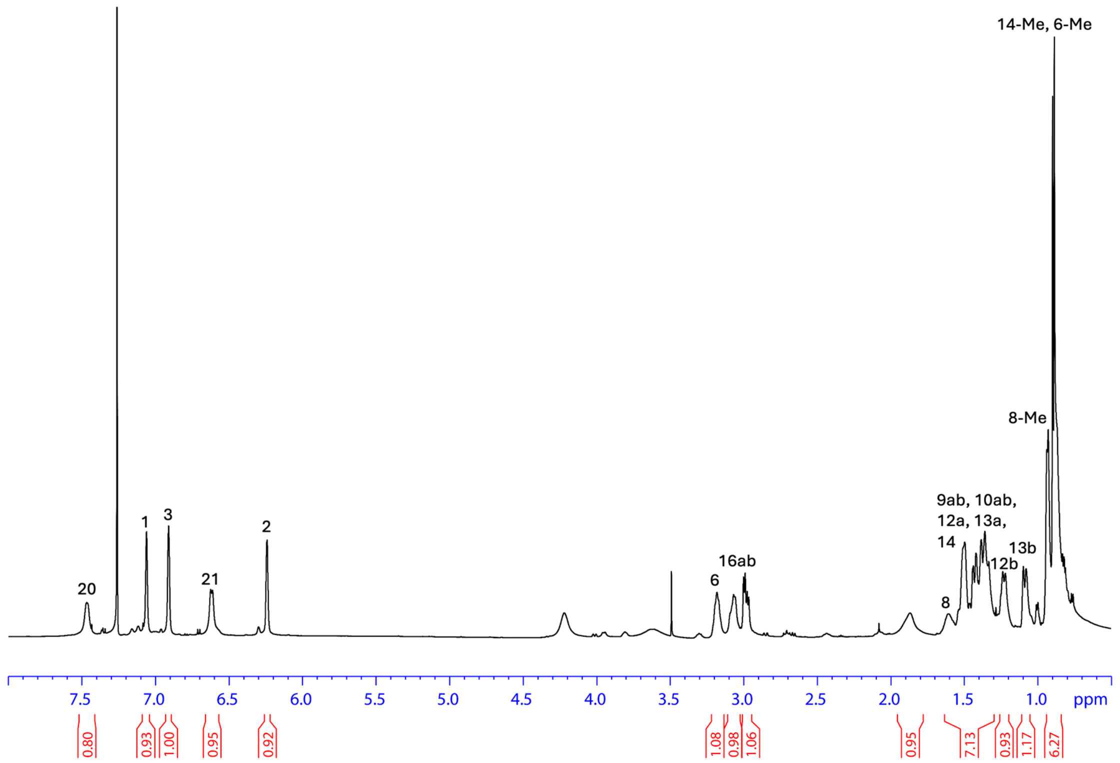

Figure S22. ^{1}H NMR (600 MHz, $CDCl_3$) spectrum of goondoxazole B (**2**).

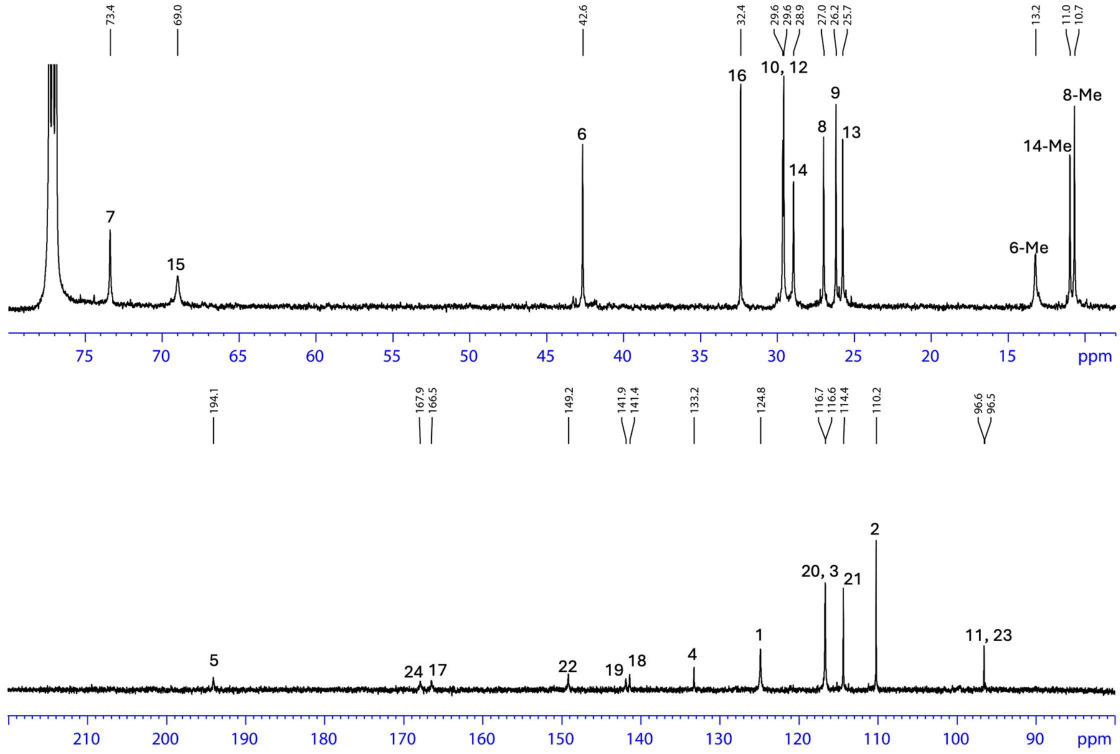

Figure S23. ^{13}C NMR (150 MHz, $CDCl_3$) spectrum of goondoxazole B (**2**).

Mass Spectrum Molecular Formula Report

Analysis Info

Analysis Name	D:\Data\Shengbin\S4S193A39_3.d	Acquisition Date	10/25/2023 4:29:44 PM	
Method	tune-med_AP.m	Operator	a.salim	
Sample Name	S4S193A39_3	Instrument / Ser#	micrOTOF	213750.00232
Comment				

Acquisition Parameter

Source Type	ESI	Ion Polarity	Positive	Set Nebulizer	0.8 Bar
Focus	Not active			Set Dry Heater	180 °C
Scan Begin	100 m/z	Set Capillary	4500 V	Set Dry Gas	5.0 l/min
Scan End	1000 m/z	Set End Plate Offset	-500 V	Set Divert Valve	Source

Generate Molecular Formula Parameter

Formula, min.		
Formula, max.		
Measured m/z	Tolerance	Charge
Check Valence	Minimum	Maximum
Nirogen Rule	Electron Configuration	
Filter H/C Ratio	Minimum	Maximum
Estimate Carbon		

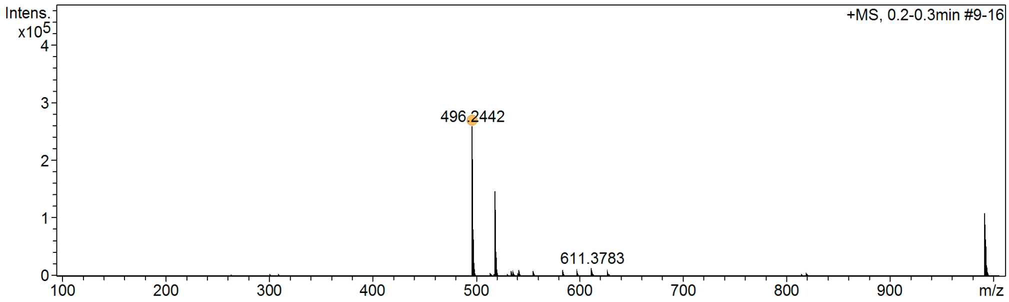

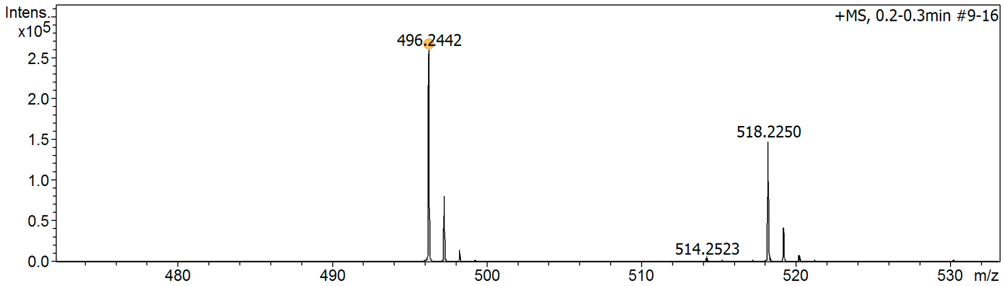

Meas. m/z	#	Ion Formula	m/z	err [ppm]	mSigma	# Sigma	Score	rdb	e⁻ Conf	N-Rule
496.2442	1	C27H34N3O6	496.2442	0.0	2.1	1	100.00	12.5	even	ok

Bruker Compass DataAnalysis 4.1 printed: 10/25/2023 4:36:10 PM Page 1 of 1

Figure S24. HRESIMS spectrum for goondoxazole B (**2**).

Spectroscopic characterisation of goondoxazole C (3)

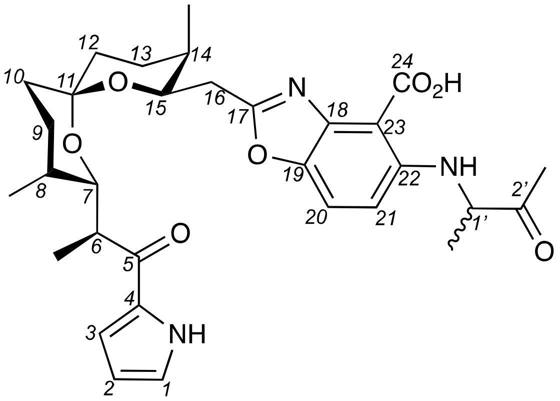

Table S6. 1D and 2D NMR (DMSO-d_6) data for goondoxazole C (**3**).

**Pos.**	δ_H, mult (*J* in Hz)	δ_C, type	COSY	^{1}H-^{13}C HMBC	ROESY
1	7.01, br s	125.1, CH	2, 1-NH	2, 3, 4	2, 1-NH
2	6.12, ddd (*3.5*, *2.5*, *2.3*)	109.4, CH	1, 3, 1-NH	1, 3, 4	1, 3
3	6.84, br s	116.3, CH	2, 1-NH	1, 2, 4	2, 6, 1-NH
4	-	132.6, C			
5	-	192.2, C			
6	3.08, dqd (*10.5*, *6.8*, *1.5*)	41.6, CH	7, 6-Me	5, 7, 6-Me	8-Me
7	3.32*, m	72.4, CH	6	5, 6, 6-Me, 8-Me	8, 15, 6-Me
8	1.39[A], m	26.4, CH	9a, 8-Me		9a, 9b, 6-Me, 8-Me
9	*a*. 1.74, m	25.5, CH$_2$	8, 9b, 10a, 10b		8, 9b
	b. 1.23[B], m		9a	7, 8, 11	9a
10	*a*. 1.41[A], m	29.2, CH$_2$	9a, 10b	9	10b
	b. 1.28[B], m		9a, 10a	9, 11	10a
11	-	95.5, C			
12	*a*. 1.37[C], m	28.9, CH$_2$	12b	14	12b, 15
	b. 1.11[D], m		12a	11, 14	12a
13	*a*. 1.36[C], m	25.3, CH$_2$	13b	14-Me	13b
	b. 1.08[D], m		13a, 14	11, 15, 14-Me	13a, 14
14	1.54, m	29.5, CH	13b, 15, 14-Me		13b, 15, 16a, 16b
15	4.13, m	68.5, CH	14, 16	17, 14-Me	7, 12a, 14
16	*a*. 2.96, dd (*14.6*, *9.6*)	32.5, CH$_2$	15	15, 17	14, 14-Me
	b. 2.92, dd (*14.6*, *4.1*)		15	15	14, 14-Me
17	-	166.8, C			
18	-	141.4, C			
19	-	141.2, C			
20	7.78, d (*9.0*)	117.1, CH	21	18, 22	
21	6.67, dd (*9.0*, *1.2*)	109.5, CH	20	19, 23, 24[#]	1', 1'-Me
22	-	147.8, C			
23	-	98.8, C			
24	-	167.8, C			
6-Me	0.61, dd (*7.2*, *6.8*)	13.2, CH$_3$	6	5, 6, 7	7, 8
8-Me	0.85, br d (*6.9*)	10.7, CH$_3$	8	7, 8, 9	6, 8
14-Me	0.89, br d (*7.0*)	10.9, CH$_3$	14	13, 14, 15	16a, 16b
1'	4.46, dq (*7.4*, *6.9*)	57.3, CH	1'-Me, 22-NH	2', 1'-Me	21, 2'-Me, 22-NH
2'	-	208.4, C			
3'	2.18, d (*0.8*)	26.0, CH$_3$		1', 2'	1', 1'-Me
1'-Me	1.38[C], d (*6.9*)	17.3, CH$_3$	1'	1', 2'	21, 22-NH
1-NH	11.75, br s	-	1, 2, 3	2, 3, 4	1, 3
22-NH	8.44, br t (*7.4*)	-	1'	21, 23[#]	1', 1'-Me

[A-D] - resonance with the same superscript overlap, [#] weak signals, * obscured by solvent.

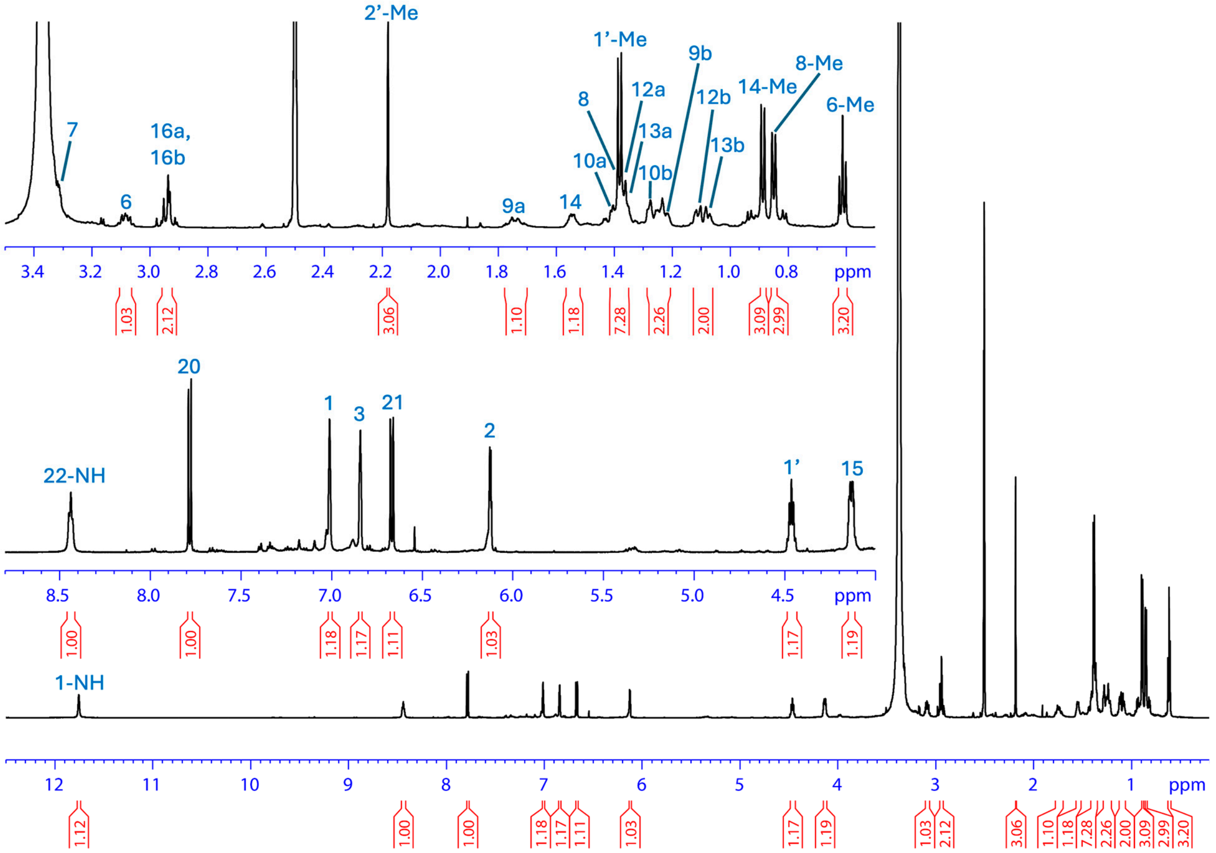

Figure S25. ^{1}H NMR (600 MHz, DMSO-d_6) spectrum of goondoxazole C (**3**).

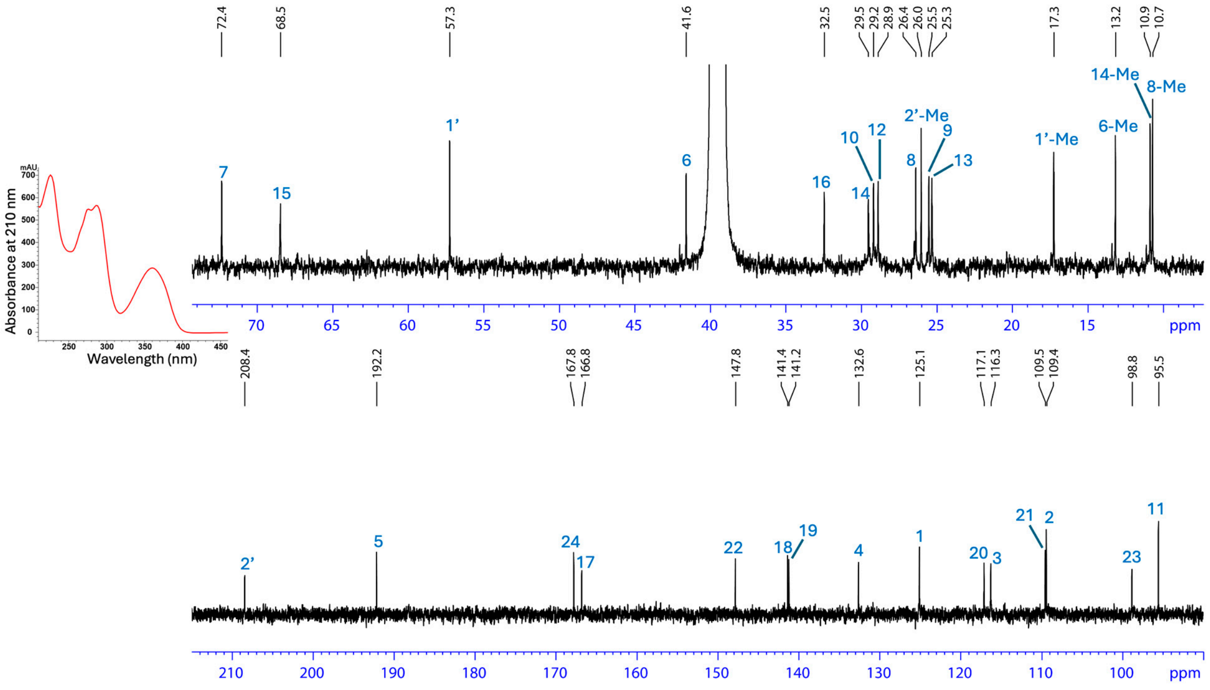

Figure S26. ^{13}C NMR (150 MHz, DMSO-d_6) and UV-vis (inset) spectra of goondoxazole C (**3**).

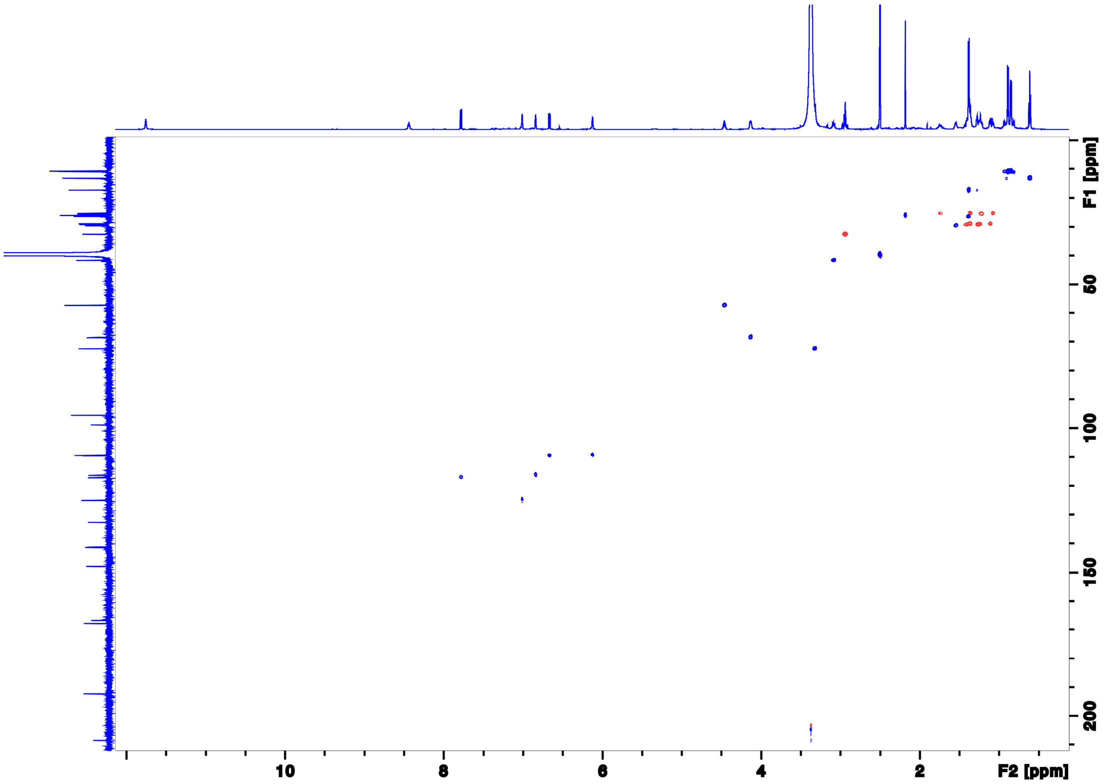

Figure S27. HSQC NMR (DMSO-d_6) spectrum of goondoxazole C (**3**).

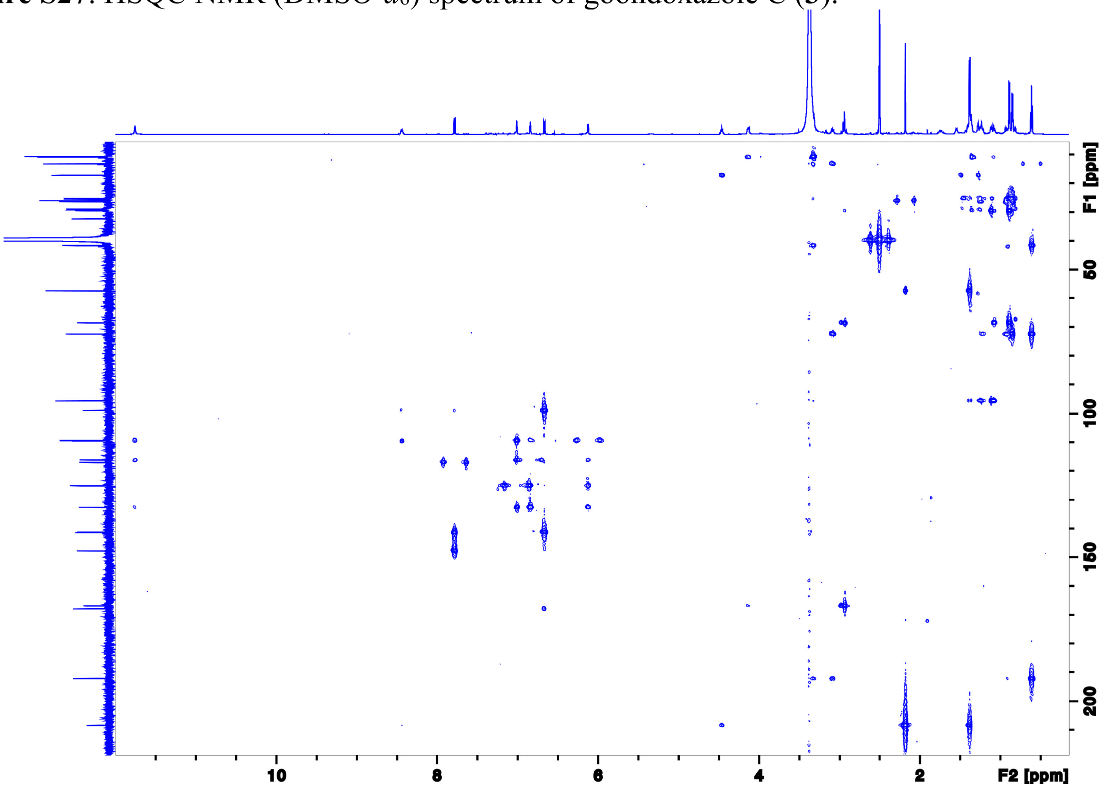

Figure S28. HMBC NMR (DMSO-d_6) spectrum of goondoxazole C (**3**).

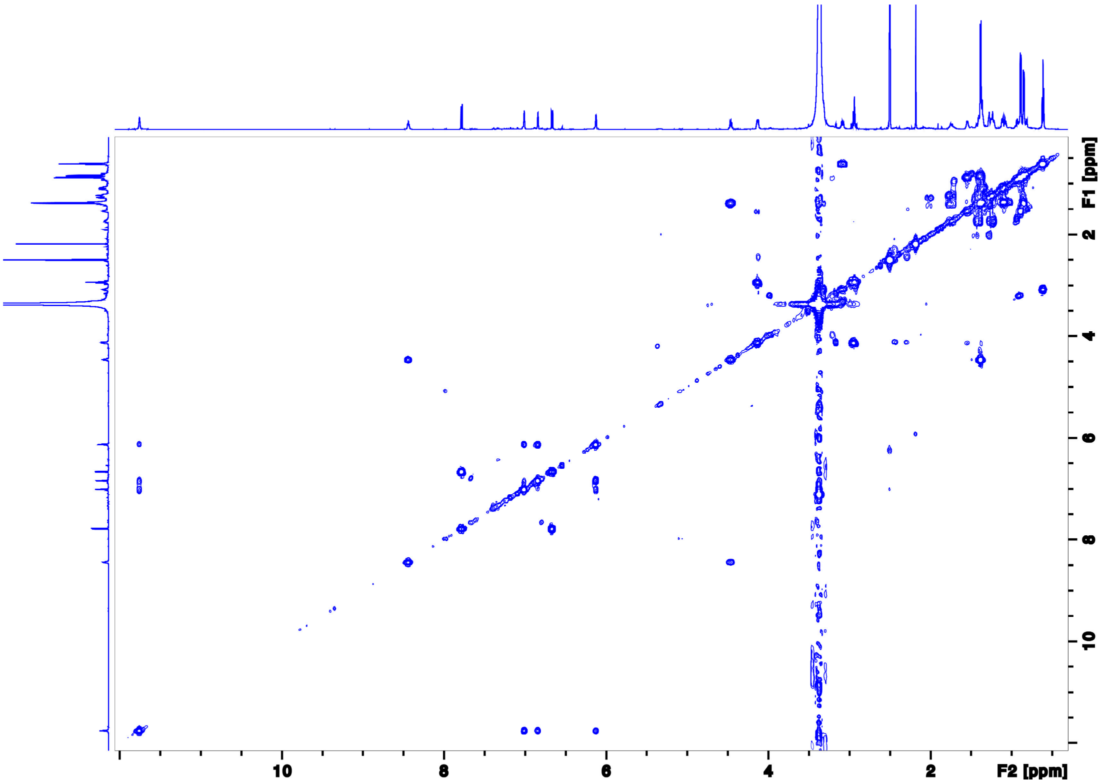

Figure S29. COSY NMR (DMSO-d_6) spectrum of goondoxazole C (**3**).

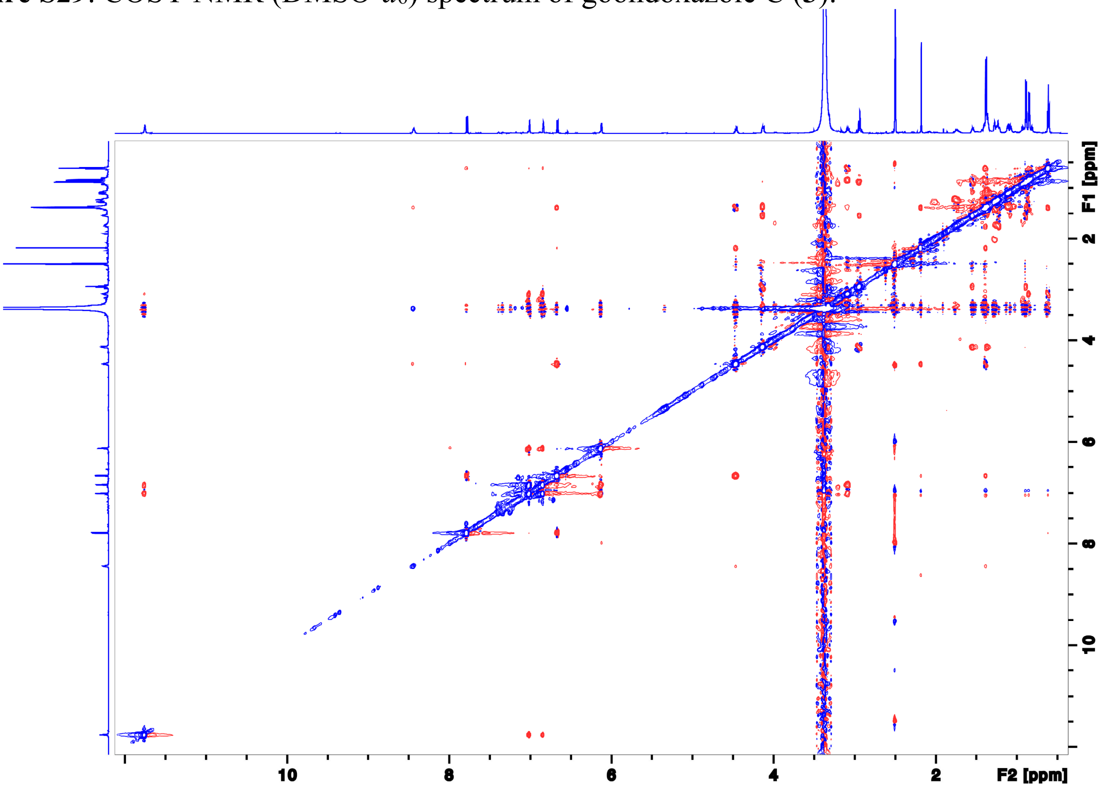

Figure S30. ROESY NMR (DMSO-d_6) spectrum of goondoxazole C (**3**).

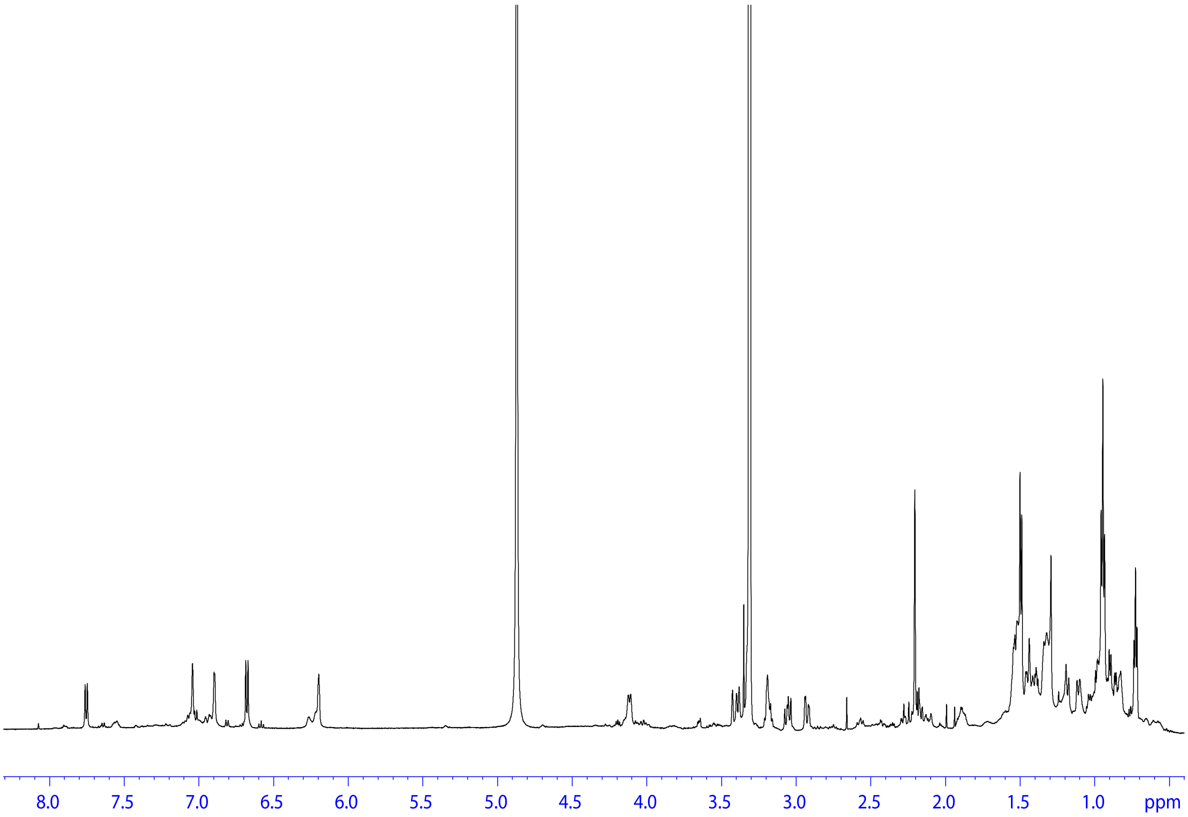

Figure S31. ^{1}H NMR (600 MHz, methanol-d_4) spectrum of goondoxazole C (**3**).

Mass Spectrum Molecular Formula Report

Analysis Info

Analysis Name	D:\Data\Shengbin\S4S193A39_C-9.d	Acquisition Date	10/25/2023 4:11:32 PM
Method	tune-medhigh_AP.m	Operator	a.salim
Sample Name	S4S193A39_C-9	Instrument / Ser#	micrOTOF 213750.00232
Comment			

Acquisition Parameter

Source Type	ESI	Ion Polarity	Positive	Set Nebulizer	0.5 Bar
Focus	Not active			Set Dry Heater	180 °C
Scan Begin	100 m/z	Set Capillary	4500 V	Set Dry Gas	5.0 l/min
Scan End	1500 m/z	Set End Plate Offset	-500 V	Set Divert Valve	Source

Generate Molecular Formula Parameter

Formula, min.					
Formula, max.					
Measured m/z		Tolerance		Charge	
Check Valence		Minimum		Maximum	
Nirogen Rule		Electron Configuration			
Filter H/C Ratio		Minimum		Maximum	
Estimate Carbon					

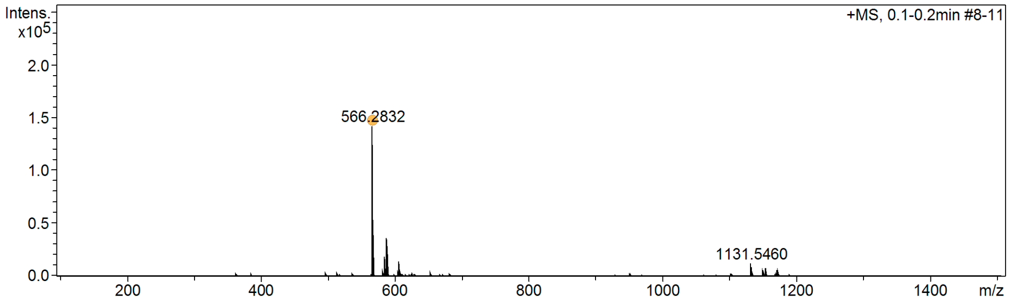

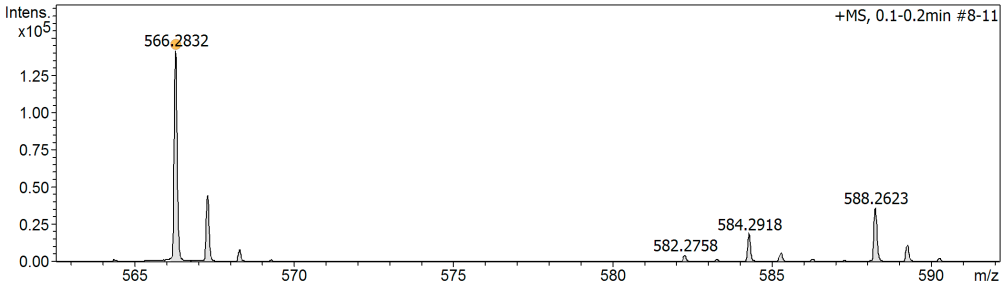

Meas. m/z	#	Ion Formula	m/z	err [ppm]	mSigma	# Sigma	Score	rdb	e⁻ Conf	N-Rule
566.2832	1	C31H40N3O7	566.2861	5.1	21.7	7	12.48	13.5	even	ok

Bruker Compass DataAnalysis 4.1 printed: 10/25/2023 3:48:52 PM Page 1 of 1

Figure S32. HRESIMS spectrum for goondoxazole C (**3**).

Spectroscopic characterisation of calcimycin (5)

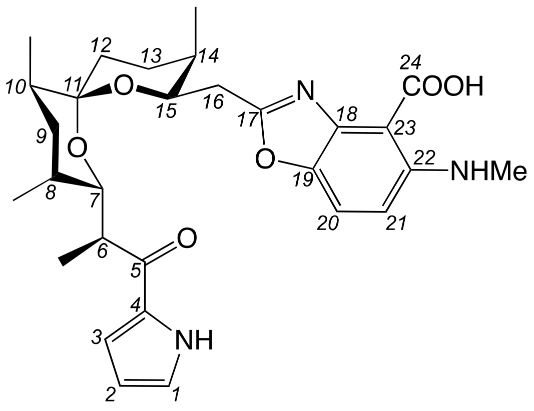

Table S7. ^{1}H NMR (DMSO-d_6) data for goondoxazole A (**1**) and calcimycin (**5**).

Pos.	(**1**)<br>δ_H, mult (*J* in Hz)	(**5**)<br>δ_H, mult (*J* in Hz)
1	7.01, br s	7.04, br s
2	6.12, ddd (*3.6*, *2.4*, *2.3*)	6.12, br s
3	6.84, br s	6.84, br s
4	-	-
5	-	-
6	3.09, dq (*10.3*, *6.9*)	3.08, br s
7	3.34*, m	3.34*, m
8	1.40^{A}, m	1.41^{A}, m
9	*a*. 1.74, dddd (*13.8*, *13.3*, *5.4*, *4.0*)	1.45^{A}, m
	b. 1.22^{B}, m	1.14, m
10	*a*. 1.41^{A}, m	1.51^{B}, m
	b. 1.26^{B}, m	-
11	-	-
12	*a*. 1.37^{C}, m	1.65, m
	b. 1.11^{D}, m	0.88^{C}, m
13	*a*. 1.36^{C}, m	1.32, m
	b. 1.07^{D}, m	1.05, m
14	1.54, m	1.53^{B}, m
15	4.13, ddd (*9.6*, *4.0*, *3.2*)	4.13, m
16	*a*. 2.94, dd (*14.6*, *9.6*)	2.93^{D}, m
	b. 2.90, dd (*14.6*, *4.0*)	2.90^{D}, m
17	-	-
18	-	-
19	-	-
20	7.66, d (*9.0*)	7.77, br s
21	6.80, d (*9.0*)	6.73, br s
22	-	-
23	-	-
24	-	-
6-Me	0.64, d (*6.9*)	0.65, d (*5.6*)
8-Me	0.85, d (*6.9*)	0.86^{C}, d (*6.1*)
10-Me	-	0.80, d (*6.2*)
14-Me	0.88, d (*6.9*)	0.86^{C}, d (*6.1*)
1-NH	11.76, br s	11.79, br s

$^{A-D}$ - resonance with the same superscript overlap, $^{\#}$ weak signals, * obscured by solvent.

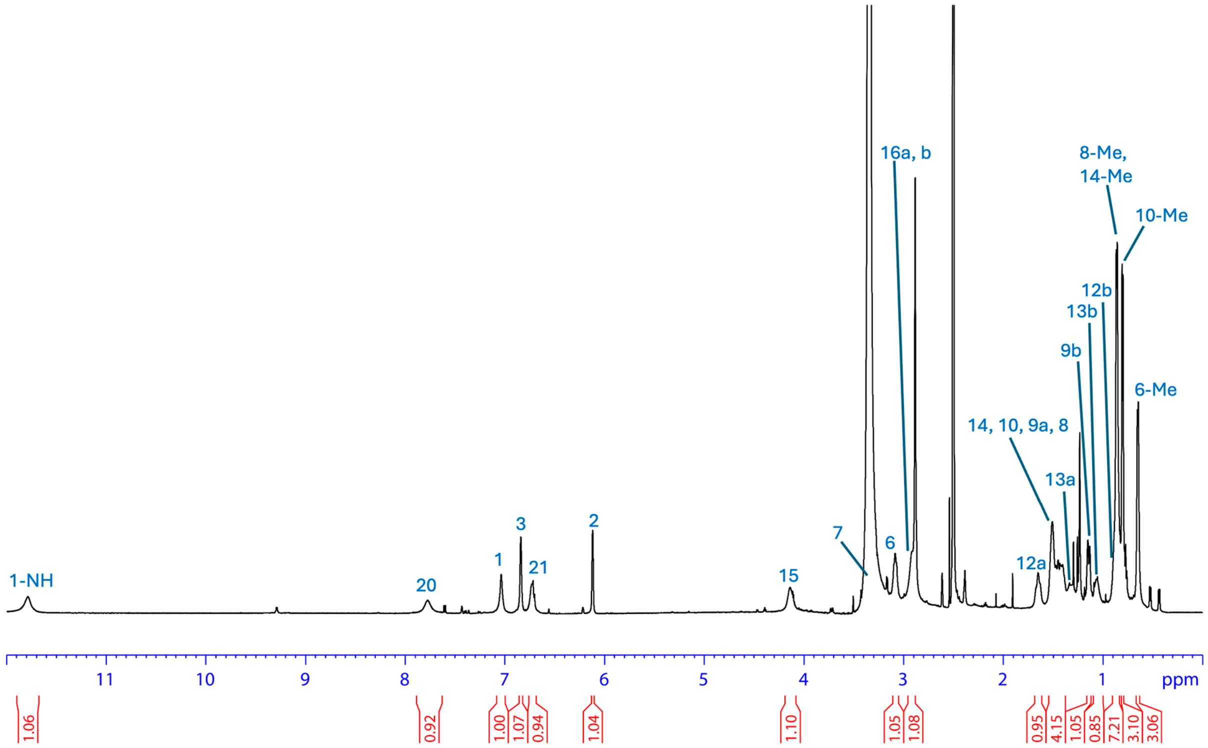

Figure S33. ^{1}H NMR (600 MHz, DMSO-d_6) spectrum of calcimycin (**5**).

Spectroscopic characterisation of A-33853 (12)

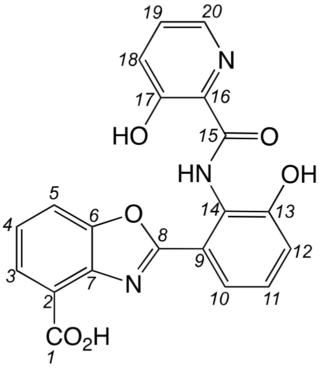

Table S8. 1D and 2D NMR (DMSO-d_6) data for A-33853 (**12**).

Pos.	δ_H, mult (*J* in Hz)	δ_C, type	COSY	^{1}H-^{13}C HMBC
1	-	165.8, C		
2	-	122.9, C		
3	7.87, br d (*7.7*)	126.5, CH	4	1, 5, 7
4	7.45, dd (*8.2*, *7.7*)	125.0, CH	3, 5	2 ,6
5	7.78, br d (*8.2*)	114.6, CH	4	3, 7
6	-	150.5, C		
7	-	140.3, C		
8	-	162.7, C		
9	-	123.7, C		
10	7.68, dd (*7.8*, *1.0*)	120.6, CH	11	8, 12, 14
11	7.39, dd (*8.1*, *7.9*)	127.8, CH	10, 12	9, 12#, 13
12	7.23, dd (*8.1*, *1.0*)	119.7, CH	11	10, 13#, 14
13	-	153.3, C		
14	-	122.6, C		
15	-	167.6, C		
16	-	131.3, C		
17	-	157.2, C		
18	7.44, br d (*8.5*)	125.9, CH	19	16, 20
19	7.60, dd (*8.5*, *4.3*)	129.4, CH	18, 20	17, 18
20	8.29, dd (*4.3*, *1.0*)	140.1, CH	19	16, 18, 19
13-OH	10.18, br s	-		12#, 13#, 14

weak signals.

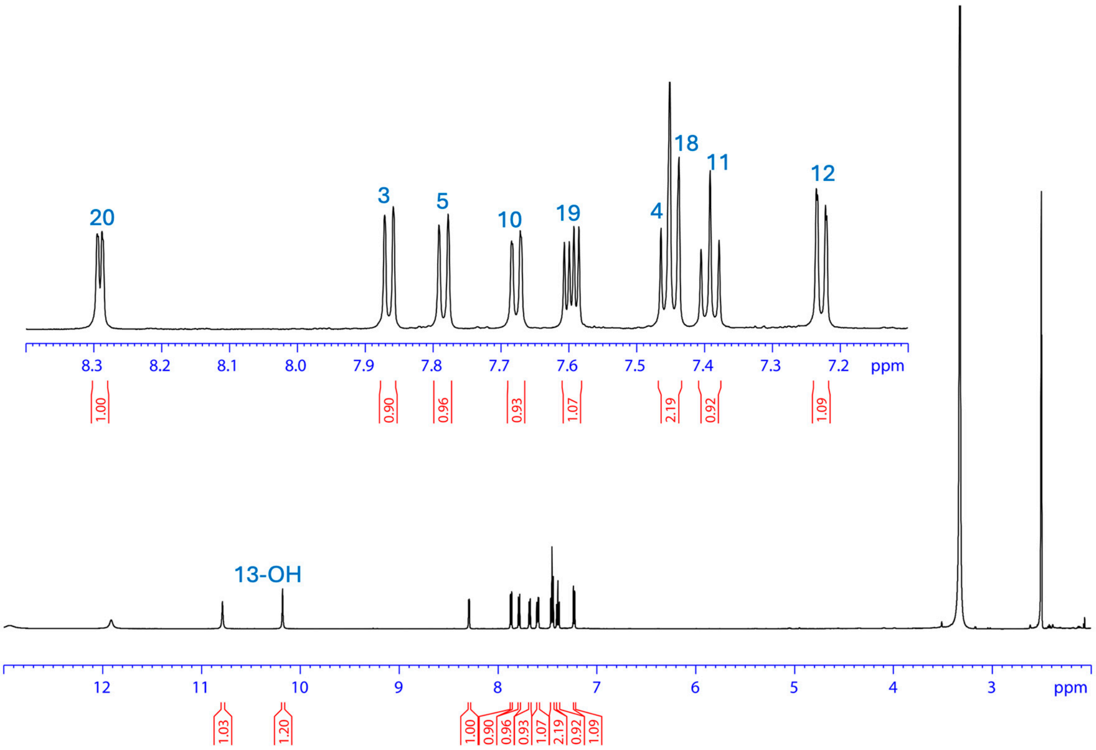

Figure S34. ^{1}H NMR (600 MHz, DMSO-d_6) spectrum of A-33853 (**12**).

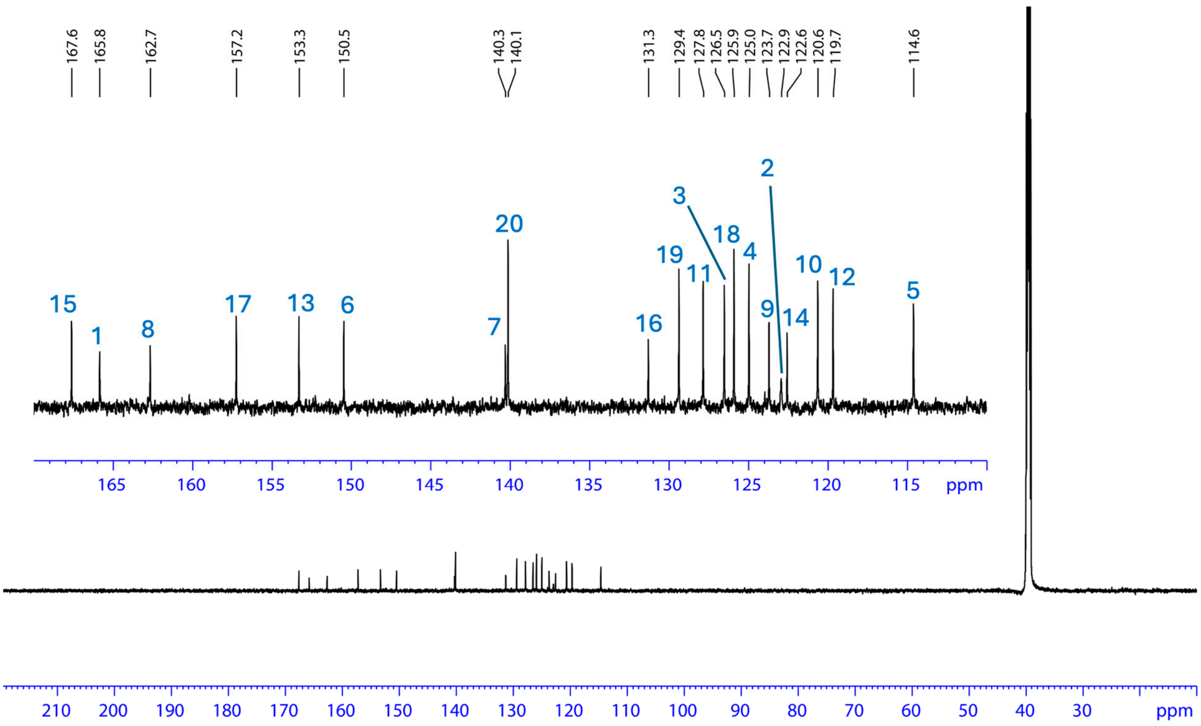

Figure S35. ^{13}C NMR (150 MHz, DMSO-d_6) spectrum of A-33853 (**12**).

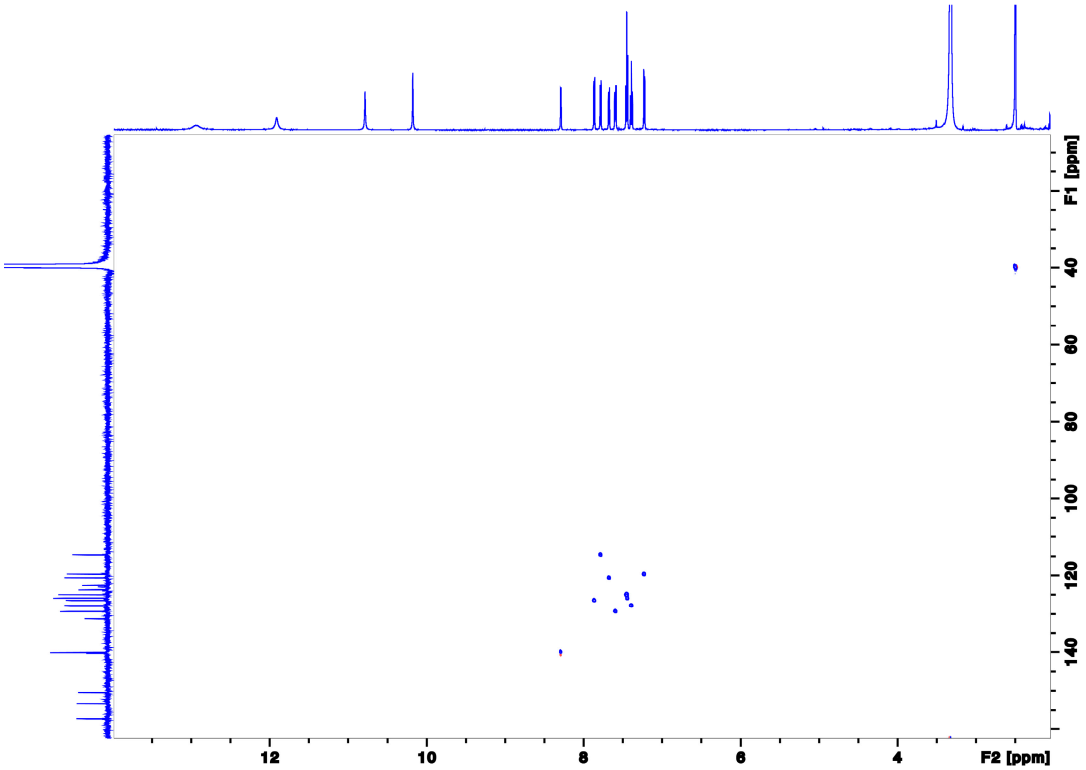

Figure S36. HSQC NMR (DMSO-d_6) spectrum of A-33853 (**12**).

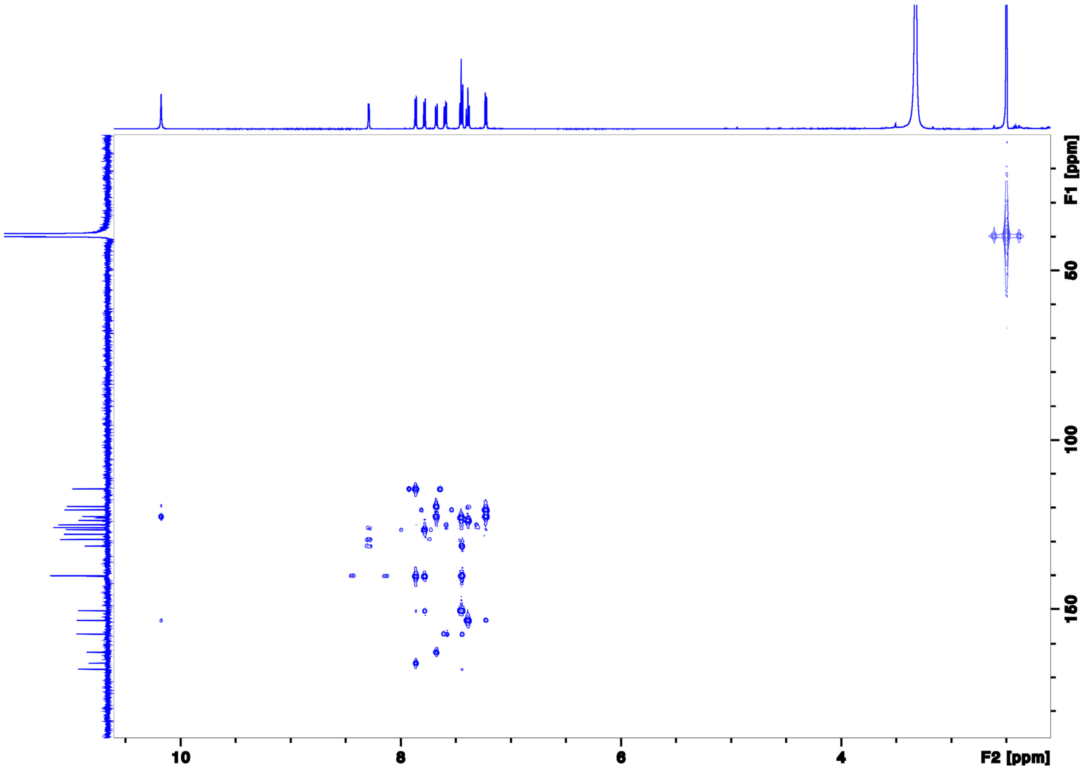

Figure S37. HMBC NMR (DMSO-d_6) spectrum of A-33853 (**12**).

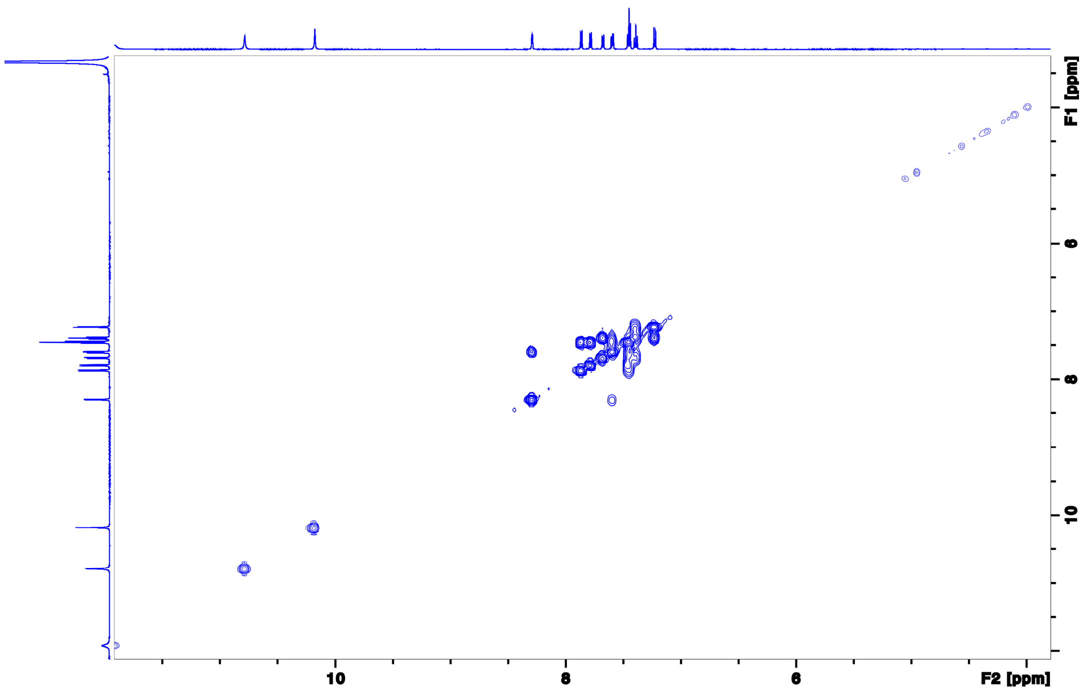

Figure S38. COSY NMR (DMSO-d_6) spectrum of A-33853 (**12**).

Mass Spectrum Molecular Formula Report

Analysis Info

Analysis Name	D:\Data\Jianying\S4S-00200B03-DCM-M-P3.d	Acquisition Date	1/27/2022 2:23:24 PM
Method	tune-medhigh_AP.m	Operator	a.salim
Sample Name		Instrument / Ser#	micrOTOF 213750.00232
Comment			

Acquisition Parameter

Source Type	ESI	Ion Polarity	Positive	Set Nebulizer	0.5 Bar
Focus	Not active			Set Dry Heater	180 °C
Scan Begin	100 m/z	Set Capillary	4500 V	Set Dry Gas	5.0 l/min
Scan End	1500 m/z	Set End Plate Offset	-500 V	Set Divert Valve	Source

Generate Molecular Formula Parameter

Formula, min.		
Formula, max.		
Measured m/z	Tolerance	Charge
Check Valence	Minimum	Maximum
Nirogen Rule	Electron Configuration	
Filter H/C Ratio	Minimum	Maximum
Estimate Carbon		

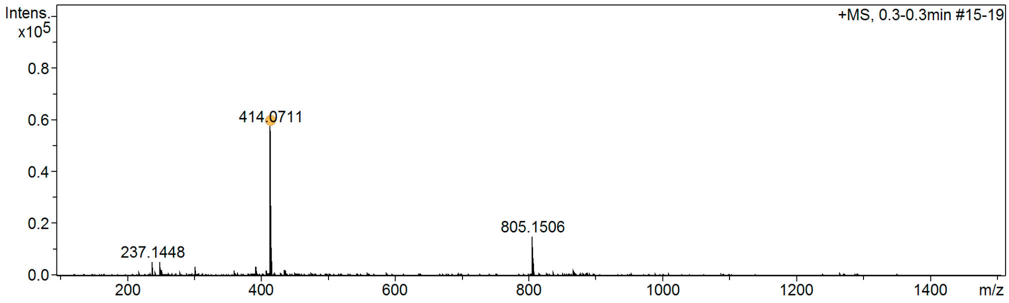

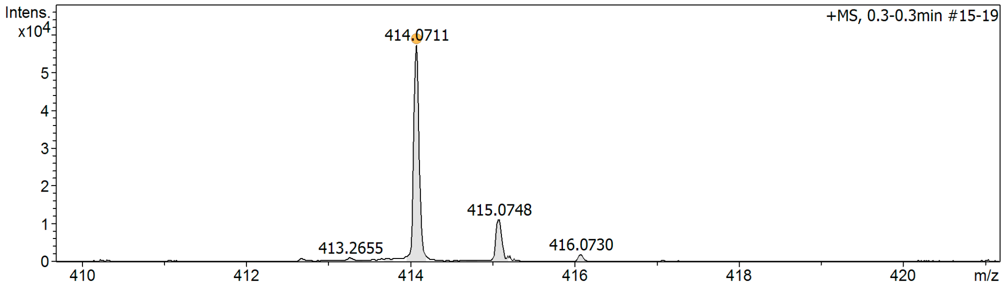

Meas. m/z	#	Ion Formula	m/z	err [ppm]	mSigma	# Sigma	Score	rdb	e⁻ Conf	N-Rule
414.0711	1	C16H9N9NaO4	414.0670	9.9	7.7	1	7.36	16.5	even	ok
	2	C20H13N3NaO6	414.0697	-3.4	17.8	2	95.75	15.5	even	ok
	3	C13H17N3NaO11	414.0755	10.8	19.0	3	5.00	6.5	even	ok
	4	C17H5N13Na	414.0683	-6.7	21.3	4	23.27	21.5	even	ok
	5	C21H9N7NaO2	414.0710	0.2	33.9	5	100.00	20.5	even	ok
	6	C25H13NNaO4	414.0737	-6.3	41.1	6	21.79	19.5	even	ok
	7	C26H9N5Na	414.0750	-9.5	52.6	7	3.91	24.5	even	ok

Bruker Compass DataAnalysis 4.1 printed: 1/27/2022 2:32:45 PM Page 1 of 1

Figure S39. HRESIMS spectrum for A-33853 (**12**).

Spectroscopic characterisation of UK-1 (13)

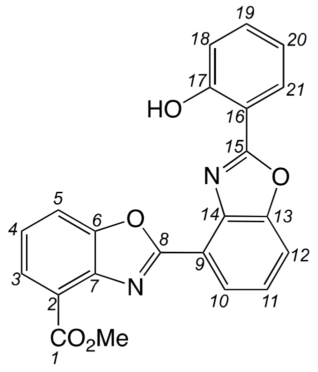

Table S9. 1D and 2D NMR (DMSO-d_6) data for UK-1 (**13**).

**Pos.**	δ_H, mult (*J* in Hz)	δ_C, type	COSY	^{1}H-^{13}C HMBC
1	-	165.3, C		
2	-	121.9, C		
3	8.03, br d (*8.0*)	127.0, CH	4	1, 5, 7
4	7.62, dd (*8.0*, *7.5*)	125.6, CH	3, 5	2, 6
5	8.16, br d (*7.5*)	115.6, CH	4	3, 7
6	-	150.9, C		
7	-	140.8, C		
8	-	161.0, C		
9	-	116.9, C		
10	8.32, br d (*7.9*)	125.1, CH	11	8, 12, 14
11	7.73, dd (*7.9*, *7.6*)	126.2, CH	10, 12	9, 13
12	8.17, br d (*7.6*)	114.8, CH	11	10, 14
13	-	149.8, C		
14	-	138.0, C		
15	-	164.3, C		
16	-	110.0, C		
17	-	158.7, C		
18	7.22, br d (*7.9*)	117.5, CH	19	
19	7.60, m	134.7, CH	18, 20	17, 21
20	7.14, dd (*7.7*, *7.2*)	120.0, CH	19, 21	16$^{\#}$, 18$^{\#}$
21	8.11, br d (*7.6*)	127.6, CH	20	15$^{\#}$, 17, 19
1-OMe	4.06, s	52.3, CH_3		1
17-OH	*	-		

$^{\#}$ weak signals, * not observed.

Table S10. ^{1}H NMR ($CDCl_3$) for UK-1 (**13**) and literature data for UK-1.

**Pos.**	(**13**) δ_H, mult (*J* in Hz)	UK-1# δ_C, mult (*J* in Hz)
1	-	-
2	-	-
3	7.89, dd (*8.1*, *0.8*)	7.84, dd
4	7.47^A, dd (*8.0*, *7.7*)	7.44, t
5	8.10, br d (*7.7*)	8.07, dd
6	-	-
7	-	-
8	-	-
9	-	-
10	8.38, br d (*7.7*)	8.31, dd
11	7.54, dd (*8.3*, *7.8*)	7.48, t
12	7.80, dd (*8.2*, *0.8*)	7.72, dd
13	-	-
14	-	-
15	-	-
16	-	-
17	-	-
18	7.21, m	7.15, d
19	7.49^A, m	7.46, t
20	7.05, dd (*8.0*, *7.6*)	7.00, td
21	8.07, br d (*8.0*)	8.01, dd
1-OMe	4.18, s	4.17, s
17-OH	-	-

^{A} - resonance with the same superscript overlap, # J. Antibiot, 1993, 46, 1095-1100.

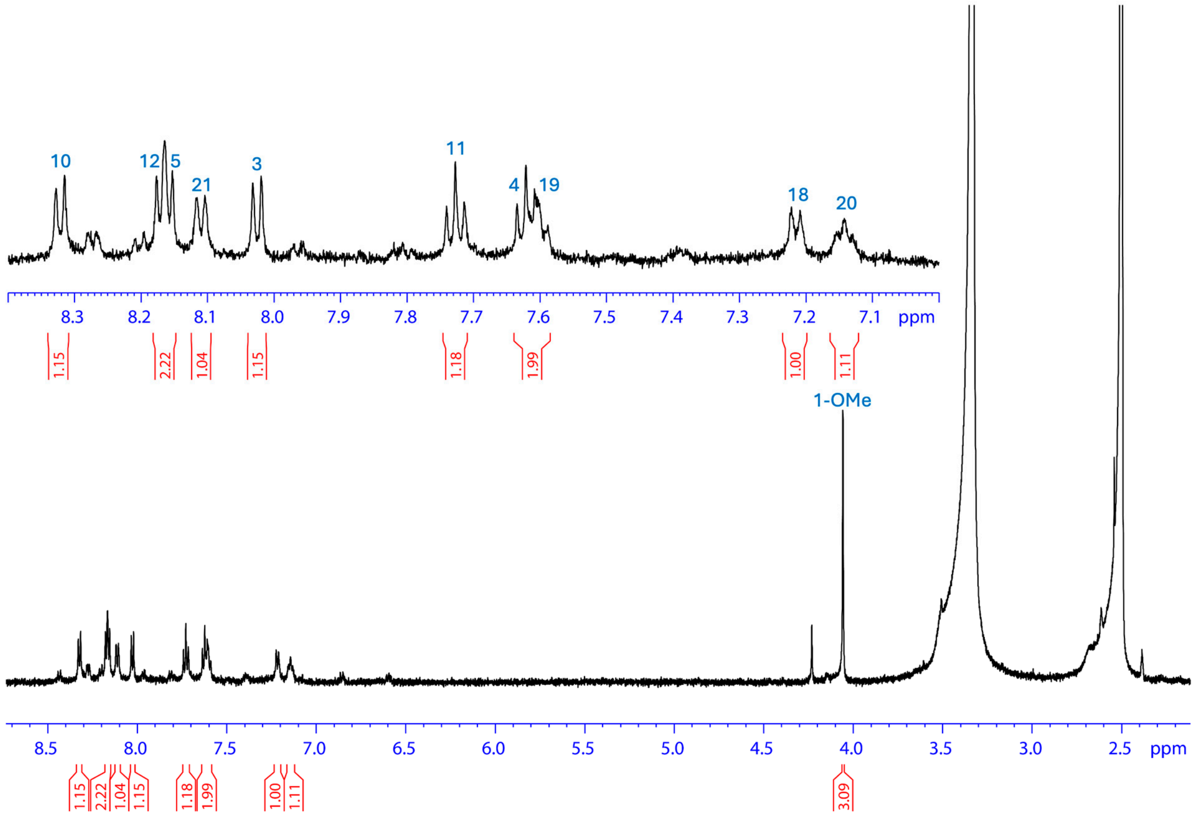

Figure S40. ^{1}H NMR (600 MHz, DMSO-d_6) spectrum of UK-1 (**13**).

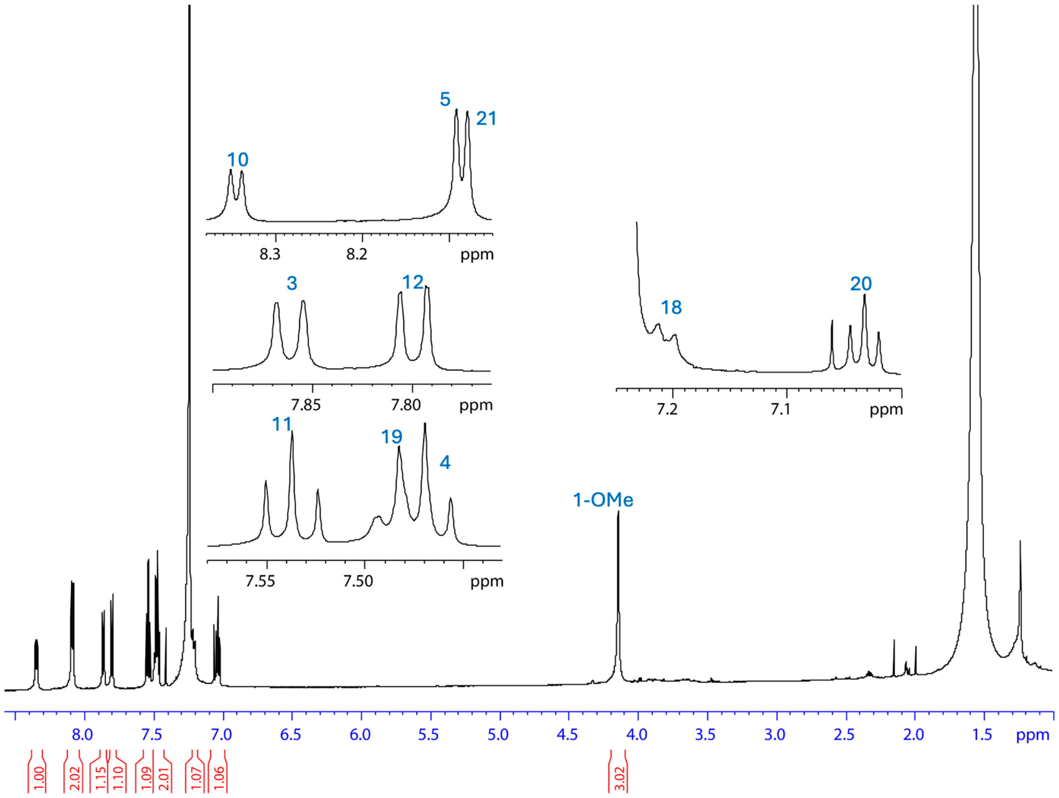

Figure S41. ^{1}H NMR (600 MHz, $CDCl_3$) spectrum of UK-1 (**13**).

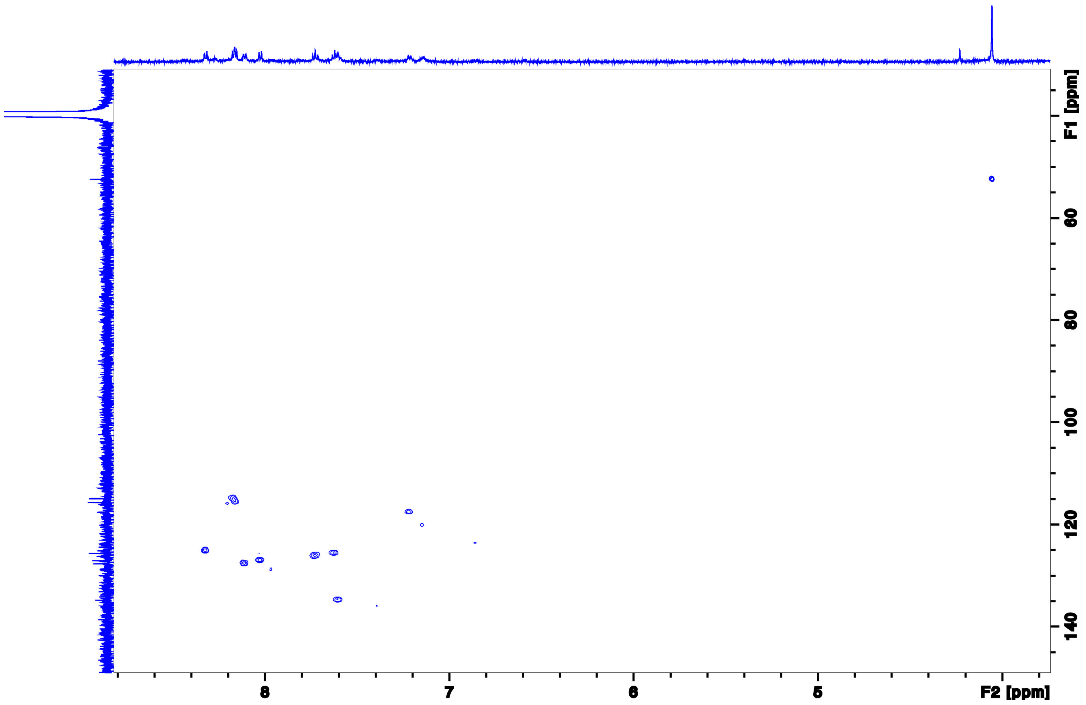

Figure S42. HSQC NMR (DMSO-d_6) spectrum of UK-1 (**13**).

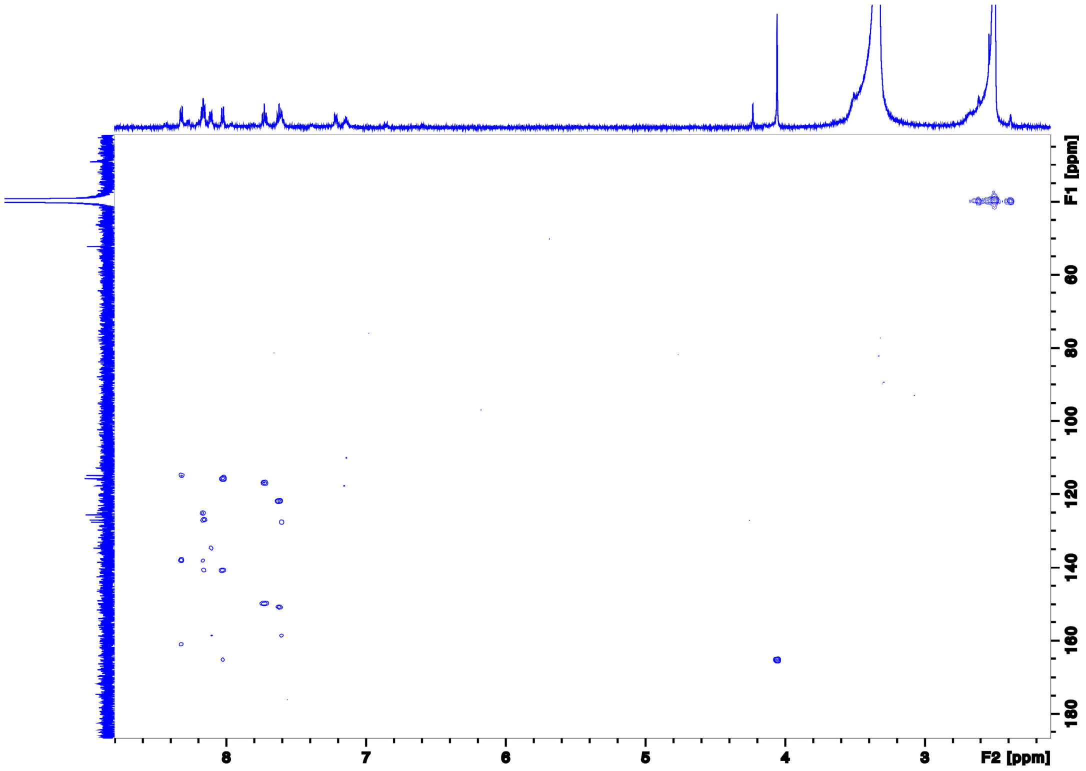

Figure S43. HMBC NMR (DMSO-d_6) spectrum of UK-1 (**13**).

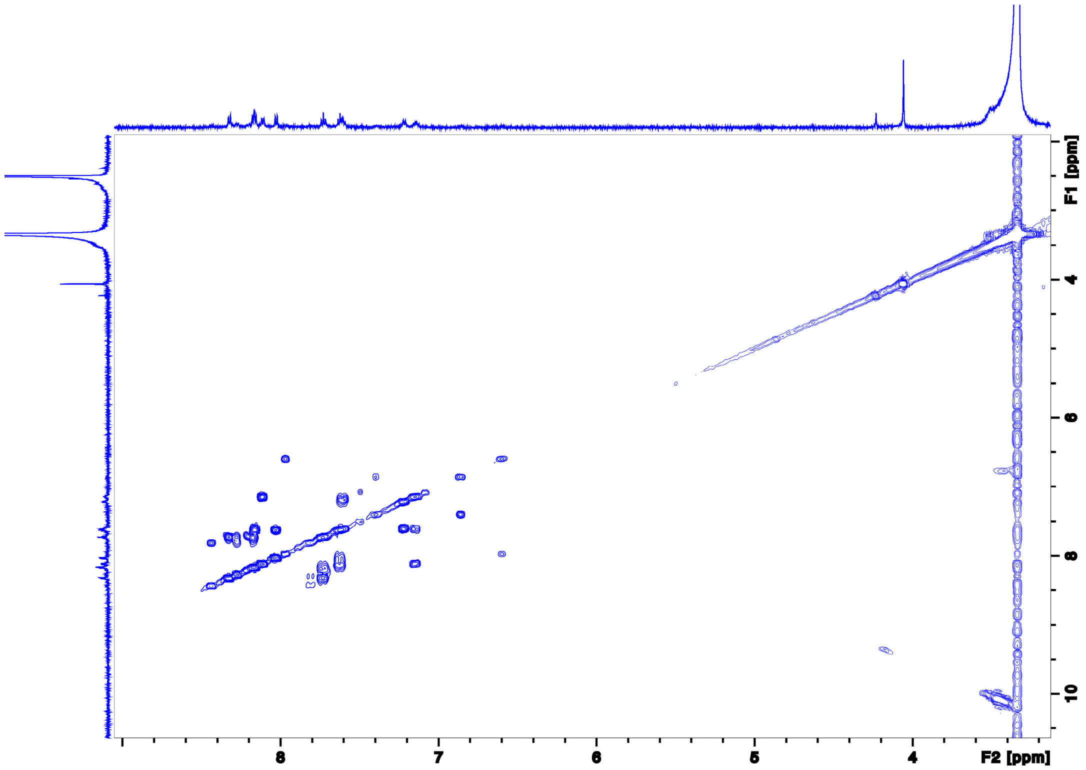

Figure S44. COSY NMR (DMSO-d_6) spectrum of UK-1 (**13**).

Spectroscopic characterisation of nataxazole (14)

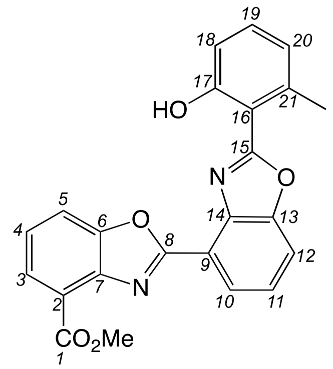

Table S11. 1D and 2D NMR (DMSO-d_6) data for nataxazole (**14**).

**Pos.**	δ_H, mult (*J* in Hz)	δ_C, type	COSY	^1H-^{13}C HMBC
1	-	165.3, C		
2	-	121.8, C		
3	8.01, dd (*7.7*, *1.0*)	127.0, CH	4	1, 5, 7
4	7.61, dd (*8.3*, *7.8*)	125.5, CH	3, 5	2, 6
5	8.14, dd (*8.2*, *1.0*)	115.7, CH	4	3, 7
6	-	151.0, C		
7	-	140.7, C		
8	-	161.4, C		
9	-	116.8, C		
10	8.33, dd (*7.8*, *0.9*)	125.1, CH	11	8, 12, 14
11	7.72, dd (*8.2*, *7.9*)	125.9, CH	10, 12	9, 13
12	8.15, dd (*8.2*, *0.9*)	114.9, CH	11	10, 14
13	-	150.1, C		
14	-	137.5, C		
15	-	164.6, C		
16	-	111.0, C		
17	-	159.3, C		
18	6.99, br d (*8.3*)	114.8, CH	19	15#, 16, 20
19	7.41, dd (*8.3*, *7.6*)	133.1, CH	18, 20	17, 21
20	6.93, br d (*7.5*)	122.1, CH	19, 21-Me	16, 18
21	-	139.3, C		
1-OMe	4.03, s	52.3, CH_3		1
17-OH	12.13, br s	-		16#, 18#
21-Me	2.64, br s	21.8, CH_3	20	15#, 16, 20, 21

weak signals.

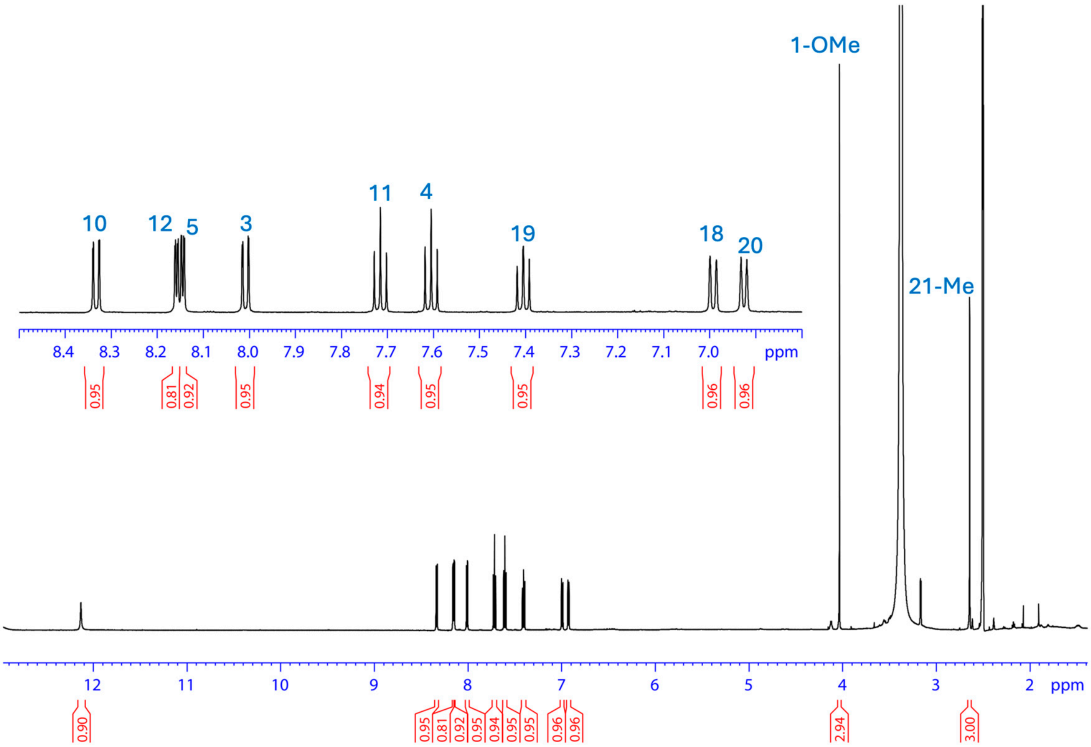

Figure S45. ^{1}H NMR (600 MHz, DMSO-d_6) spectrum of nataxazole (**14**).

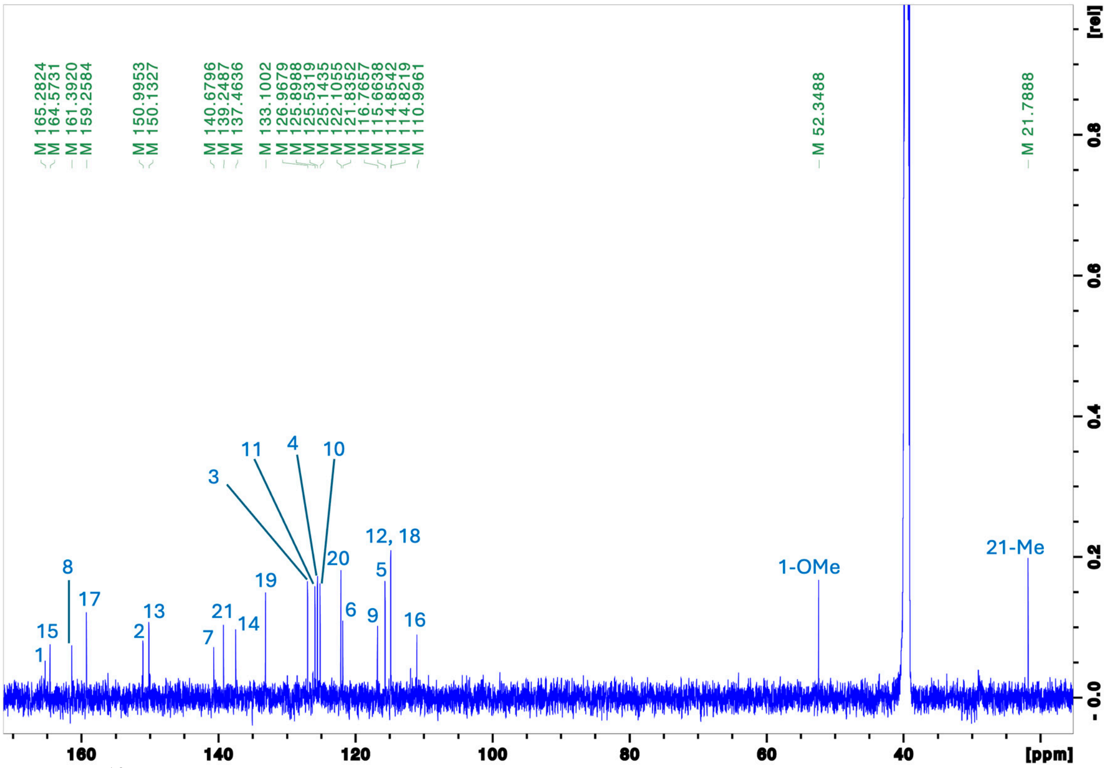

Figure S46. ^{13}C NMR (150 MHz, DMSO-d_6) spectrum of nataxazole (**14**).

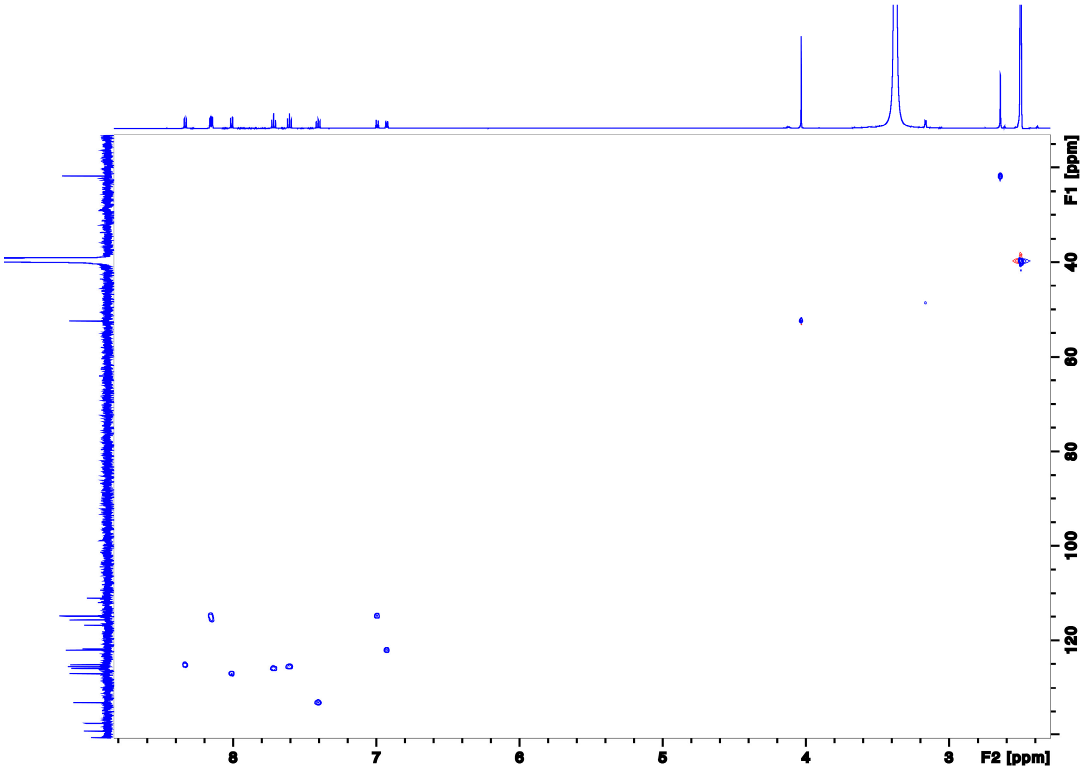

Figure S47. HSQC NMR (DMSO-d_6) spectrum of nataxazole (**14**).

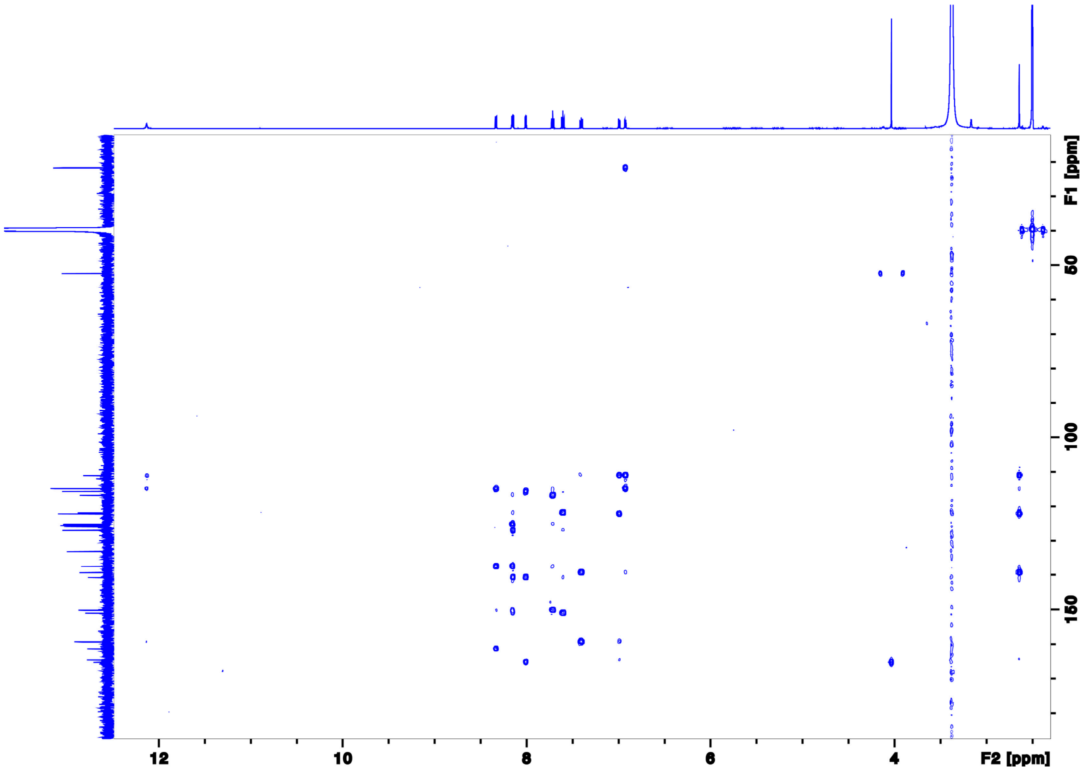

Figure S48. HMBC NMR (DMSO-d_6) spectrum of nataxazole (**14**).

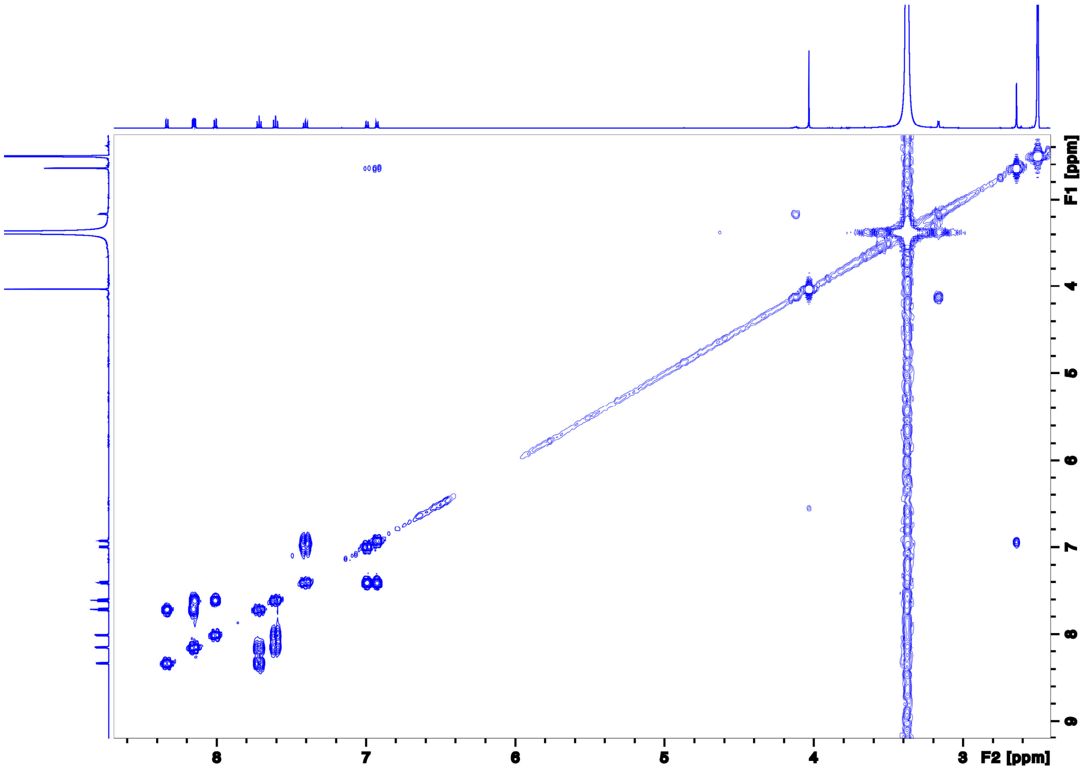

Figure S49. COSY NMR (DMSO-d_6) spectrum of nataxazole (**14**).

Mass Spectrum Molecular Formula Report

Analysis Info

Analysis Name	D:\Data\Marcelo\050520_21_15.d	Acquisition Date	8/6/2020 2:10:37 PM
Method	tune-med_AP.m	Operator	a.salim
Sample Name	PB42_C01_RMTPCI_vial 14_15	Instrument / Ser#	micrOTOF 213750.00232
Comment			

Acquisition Parameter

Source Type	ESI	Ion Polarity	Positive	Set Nebulizer	0.8 Bar
Focus	Not active			Set Dry Heater	180 °C
Scan Begin	100 m/z	Set Capillary	4500 V	Set Dry Gas	5.0 l/min
Scan End	1000 m/z	Set End Plate Offset	-500 V	Set Divert Valve	Source

Generate Molecular Formula Parameter

Formula, min.
Formula, max.
Measured m/z — Tolerance — Charge
Check Valence — Minimum — Maximum
Nirogen Rule — Electron Configuration
Filter H/C Ratio — Minimum — Maximum
Estimate Carbon

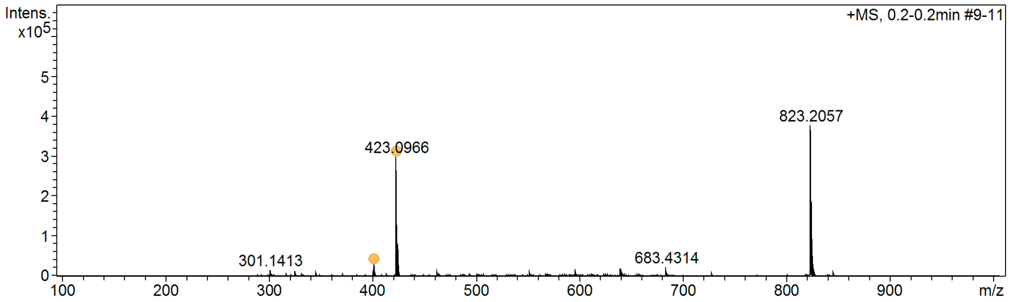

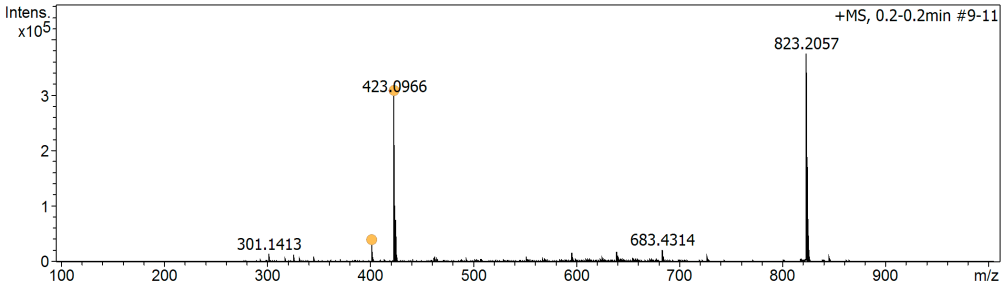

Meas. m/z	#	Ion Formula	m/z	err [ppm]	mSigma	# Sigma	Score	rdb	e⁻ Conf	N-Rule
401.1129	1	C23H17N2O5	401.1132	0.8	10.8	1	100.00	16.5	even	ok
423.0966	1	C23H16N2NaO5	423.0951	3.3	2.6	1	100.00	16.5	even	ok

Bruker Compass DataAnalysis 4.1 printed: 8/7/2020 12:32:21 PM Page 1 of 1

Figure S50. HRESIMS spectrum for nataxazole (**14**).

Spectroscopic characterisation of 5-hydroxynataxazole (15)

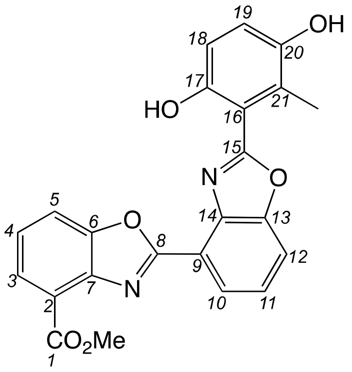

Table S12. 1D and 2D NMR (DMSO-d_6) data for 5-hydroxynataxazole (**15**).

**Pos.**	δ_H, mult (*J* in Hz)	δ_C, type	COSY	^1H-^{13}C HMBC
1	-	165.2, C		
2	-	121.8, C		
3	8.00, dd (*7.7*, *1.0*)	126.9, CH	4	1, 5, 7
4	7.60, dd (*8.3*, *7.7*)	125.5, CH	3, 5	2, 6
5	8.15, dd (*8.3*, *1.0*)	115.7, CH	4	3, 7
6	-	151.1, C		
7	-	140.6, C		
8	-	161.5, C		
9	-	116.8, C		
10	8.33, dd (*7.8*, *0.9*)	125.2, CH	11	8, 12, 14
11	7.71, dd (*8.3*, *7.9*)	125.8, CH	10, 12	9, 13
12	8.14, dd (*8.2*, *0.9*)	114.8, CH	11	10, 14
13	-	150.1, C		
14	-	137.9, C		
15	-	*		
16	-	112.3, C		
17	-	151.5, C		
18	6.81, dd (*8.7*, *0.6*)	114.6, CH	19	16, 20
19	7.00, br d (*8.7*)	120.3, CH	18	17, 21
20	-	147.9, C		
21	-	124.0, C		
1-OMe	4.02, s	52.4, CH_3		
17-OH	*	-		
21-Me	2.38, br s	13.7, CH_3		16, 20, 21

* Not observed.

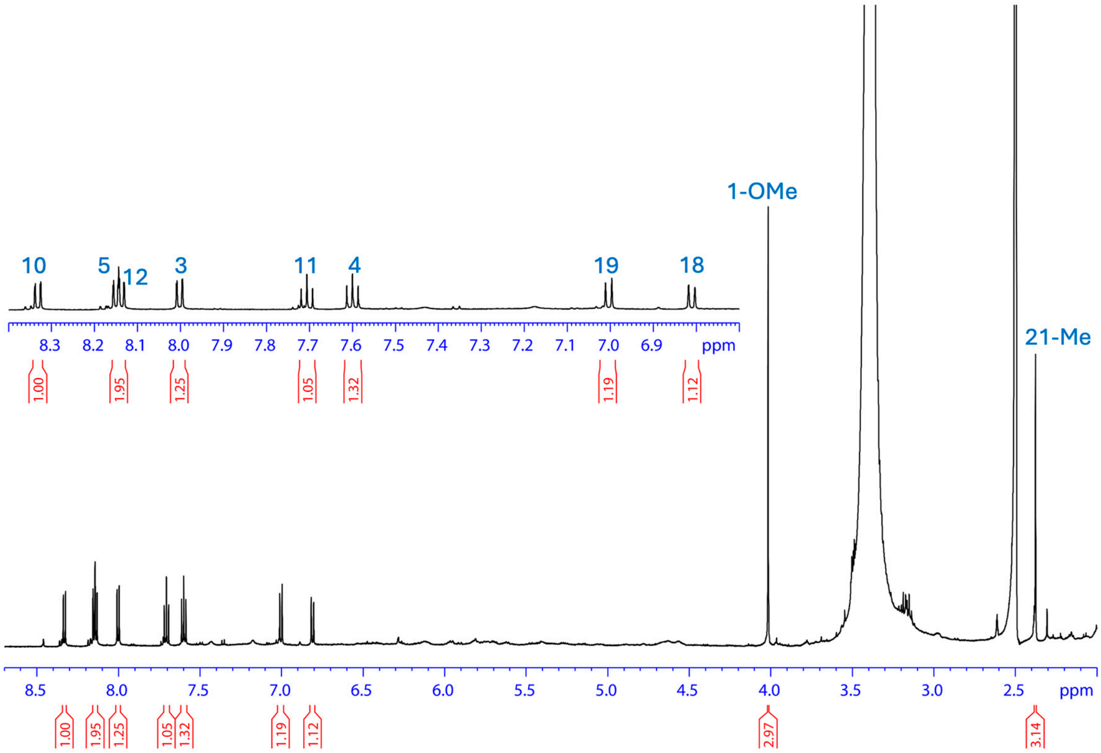

Figure S51. ^{1}H NMR (600 MHz, DMSO-d_6) spectrum of 5-hydroxynataxazole (**15**).

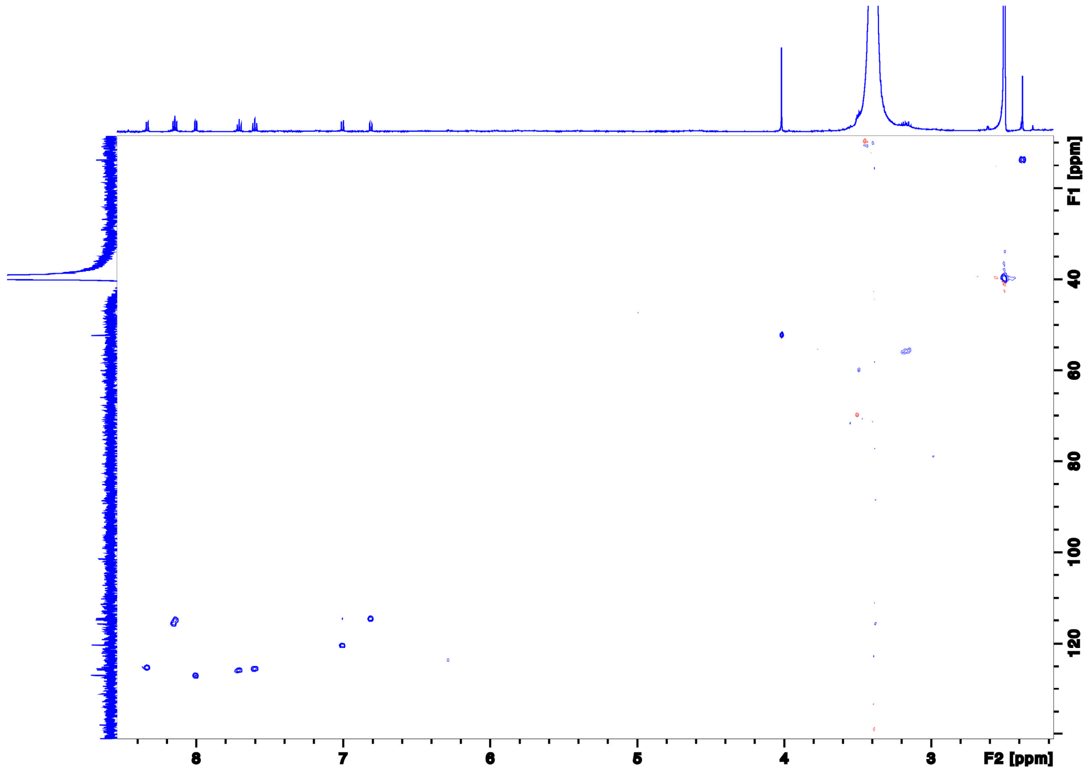

Figure S52. HSQC NMR (DMSO-d_6) spectrum of 5-hydroxynataxazole (**15**).

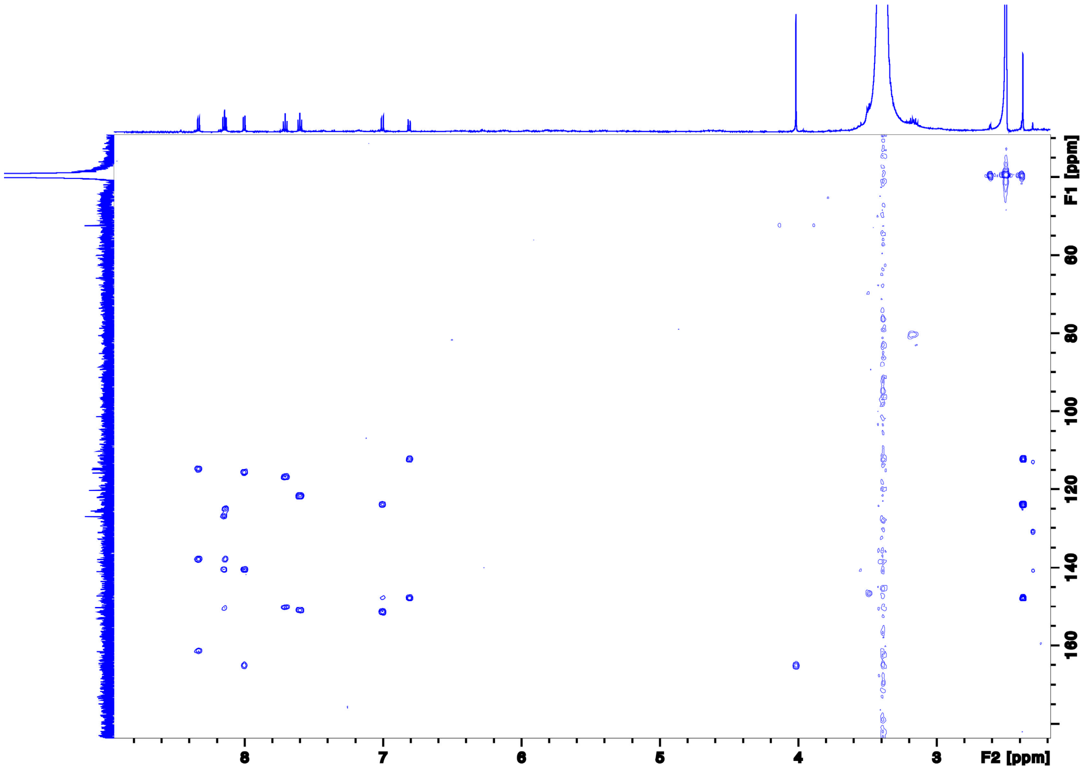

Figure S53. HMBC NMR (DMSO-*d*6) spectrum of 5-hydroxynataxazole (**15**).

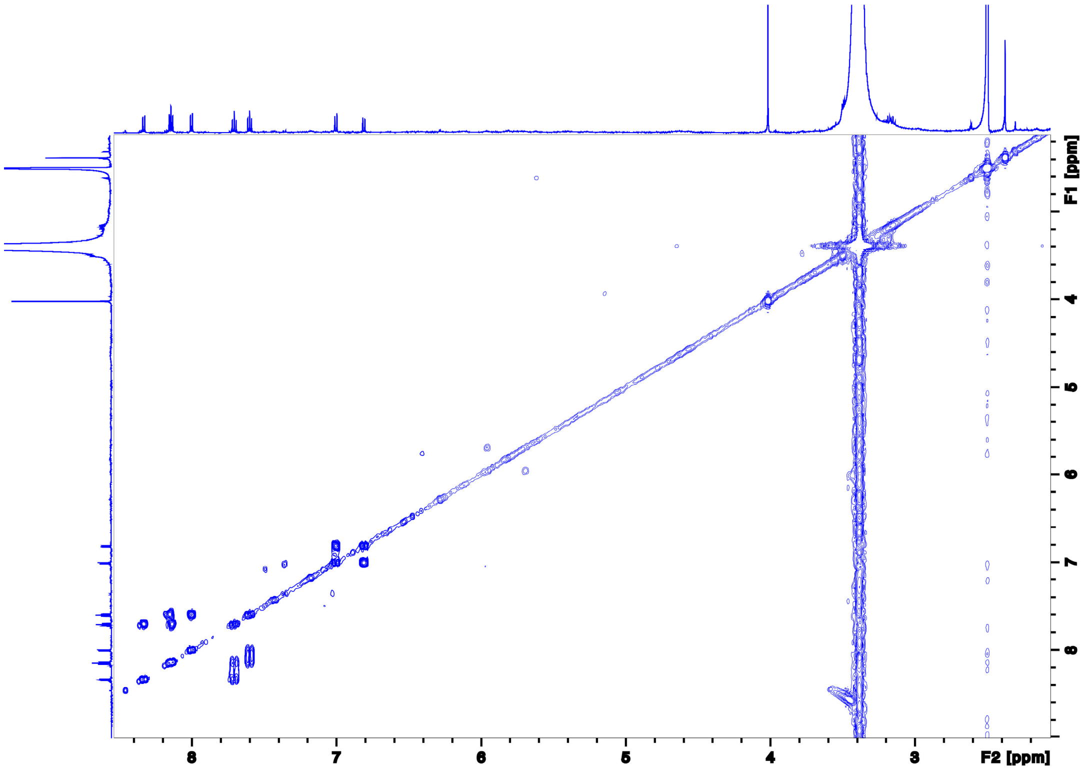

Figure S54. COSY NMR (DMSO-*d*6) spectrum of 5-hydroxynataxazole (**15**).

Mass Spectrum Molecular Formula Report

Analysis Info

Analysis Name	D:\Data\Marcelo\050520_17.d	Acquisition Date	8/6/2020 2:02:48 PM
Method	tune-med_AP.m	Operator	a.salim
Sample Name	PB42_C01_RMTPCl_vial 14_15	Instrument / Ser#	micrOTOF 213750.00232
Comment			

Acquisition Parameter

Source Type	ESI	Ion Polarity	Positive	Set Nebulizer	0.8 Bar
Focus	Not active			Set Dry Heater	180 °C
Scan Begin	100 m/z	Set Capillary	4500 V	Set Dry Gas	5.0 l/min
Scan End	1000 m/z	Set End Plate Offset	-500 V	Set Divert Valve	Source

Generate Molecular Formula Parameter

Formula, min.		
Formula, max.		
Measured m/z	Tolerance	Charge
Check Valence	Minimum	Maximum
Nirogen Rule	Electron Configuration	
Filter H/C Ratio	Minimum	Maximum
Estimate Carbon		

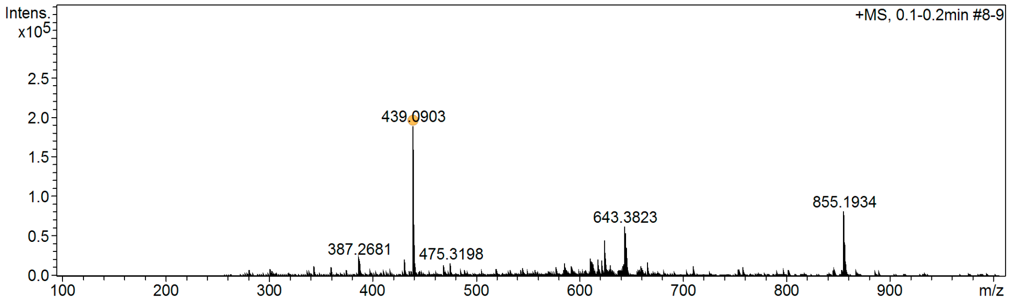

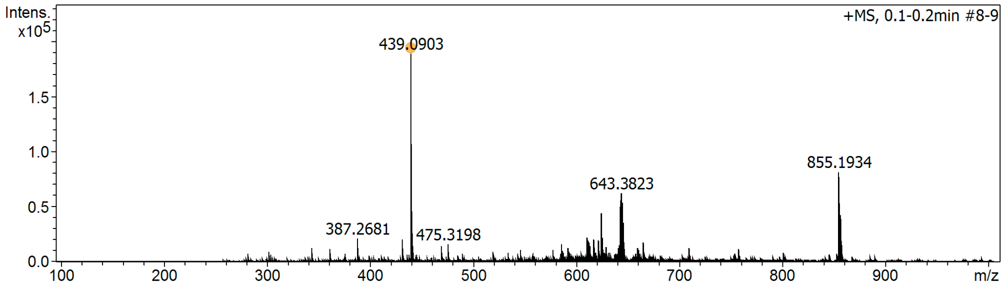

Meas. m/z	#	Ion Formula	m/z	err [ppm]	mSigma	# Sigma	Score	rdb	e⁻ Conf	N-Rule
439.0903	1	C23H16N2NaO6	439.0901	0.6	16.8	1	100.00	16.5	even	ok

Bruker Compass DataAnalysis 4.1 printed: 8/7/2020 12:29:21 PM Page 1 of 1

Figure S55. HRESIMS spectrum for 5-hydroxynataxazole (**15**).

Table S13.The rotation of **1–3** and calcimycin before and after treated metal ion.

compound	$[\alpha]^{24}_{D}$ in $CHCl_3$	NaCl added $[\alpha]^{24}_{D}$ in $CHCl_3$	$CaCl_2$ added $[\alpha]^{24}_{D}$ in $CHCl_3$	$[\alpha]^{24}_{D}$ in MeOH
goondoxazole A (**1**)	+ 110.9	+ 149.0	-	+ 65.2
goondoxazole B (**2**)	− 99.0	+ 75.6	-	+ 78.8
goondoxazole C (**3**)	– 6.2	+ 13.6	-	+ 7.5
calcimycin (**5**)	– 48.3	-	+ 25.9	+ 58.4
X-14885A (**4**)	-	+ 177.0 (lit[#])	-	-

J Am Chem Soc, 1989, 111, 8598-8609, X-14885A.

D. immitis Microfilariae Motility Inhibition Assay

D. immitis microfilariae were resuspended in RPMI 1640 media (Hyclone) and ~250 suspended parasites (in 100 µL) were delivered to microtiter plate wells containing test compounds (e.g., **1** or controls) dissolved in 100% DMSO. Test compounds were delivered to generate 10 doses (3.2-fold serial dilutions) covering the range of 25 – 0.0007 µg/mL. Microtiter plates were then incubated at 37°C and 5% CO_2 for ~72 h, after which they were imaged in a camera-based system and quantitative descriptors of parasite motility were calculated. Values were normalized using positive (1.0 µM gramicidin) and negative (DMSO) controls and motility inhibition calculated for each well. For each dose response curve, an EC_{50} value was calculated using a four-parametric logistic model in Boehringer Ingelheim’s MegaLab application.

Inhibition of Motility of *D. immitis* L4 Larvae Assay

Four to six L4 stage *D. immitis* worms suspended in a 1:1 mixture of NCTC-109 (Thermo Fisher) and iMDM [with sodium bicarbonate and 25 mM HEPES (Sigma-Aldrich)] media supplemented with antibiotics/antimycotics and 1% Fetal Bovine Serum (Cytiva Hyclone) were added to wells of a microtiter plate. Test compounds (e.g., **1** and controls) diluted in 100% DMSO prediluted in culture media were added to the wells containing the L4s for a final assay volume of 100 µL. Compounds were delivered to generate 5 doses (4-fold serial dilution) covering the range of 25 – 0.098 µg/mL. Plates were held for ~72 h at 37 °C and 5% CO_2 and imaged for calculation of motility descriptors as described above. Percentage motility inhibition is calculated by normalization of the motility descriptor values of a test compound well with the average motility of positive (5.0 µM monensin) and negative (DMSO) controls on each plate. EC_{50} values were calculated with Boehringer Ingelheim’s MegaLab application using a four-parametric logistic model.

H. contortus L1-L3 Larvae Development Assay (LDA)

~20 L1 stage *H. contortus* were delivered to microtiter plate wells containing test compounds (e.g., **1** and controls) dissolved in 100% DMSO and nutrient medium. Compounds were delivered to generate 10 doses (3.2-fold serial dilutions) in the range of 25 – 0.0007 µg/mL. Microtiter plates were then incubated at 27 °C and 85% relative humidity. After incubating ~96 h to allow development of L1 to L3 stage, plates were imaged on a camera-based system and quantitative motility descriptors calculated on resulting developed worms. Values were normalized using positive (1.0 µM ivermectin) and negative (DMSO) controls and motility inhibition calculated for each well. For each dose response curve, an EC_{50} value was calculated using a four-parametric logistic model in Boehringer Ingelheim's MegaLab application.

Cytotoxicity assay

Human colorectal (SW620) and lung (NCI-H460) carcinoma cells were cultured in Roswell Park Memorial Institute (RPMI) 1640 medium in flasks supplemented with 10% foetal bovine serum, 2 mM L-glutamine, 100 unit/mL penicillin and 100 µg/mL streptomycin in a humidified 37°C incubator supplied with 5% CO_2. Cells were then harvested with trypsin and dispensed into 96-well microtiter assay plates at 2000 cells/well in 190 µL. Compounds **1**–**3**, **5** and **12**–**15** were dissolved in DMSO and diluted with H_2O to give a 600 µM stock solution (20% DMSO) which were serially diluted with 20 % DMSO to yield concentrations ranging from 600–0.2 µM. An aliquot (10 µL) of analytes (**1**–**3**, **5** and **12**–**15**) as prepared above for cytotoxicity assays were transferred to the 96-well microtiter plate and incubated again for 24 h, after which an aliquot (10 µL) of 3-(4,5-dimethylthiazol-2-yl)-2,5-diphenyltetrazolium bromide (MTT) in sterile water (5 mg/mL) was added to each well, and the microtiter plates were then incubated for a further 3 h at 37°C with 5% CO_2. The medium was aspirated and precipitated formazan crystals were dissolved in DMSO (100 µL/well). The absorbance of each well was measured at 600 nm with a POLARstar Omega plate reader (BMG LABTECH). The negative control was 20% aqueous DMSO, while the positive controls were vinblastine and doxorubicin for both NCI-H460 (600–0.07 µM in 20% DMSO) and SW620 (600–0.2 µM in 20% DMSO). An aliquot (10 µL) of controls as prepared above were transferred to the 96-well microtiter plate. Each analysis was repeated two times, the data represented graphically, and IC_{50} values calculated using GraphPad Prism version 10.2.0 (Figure S56).

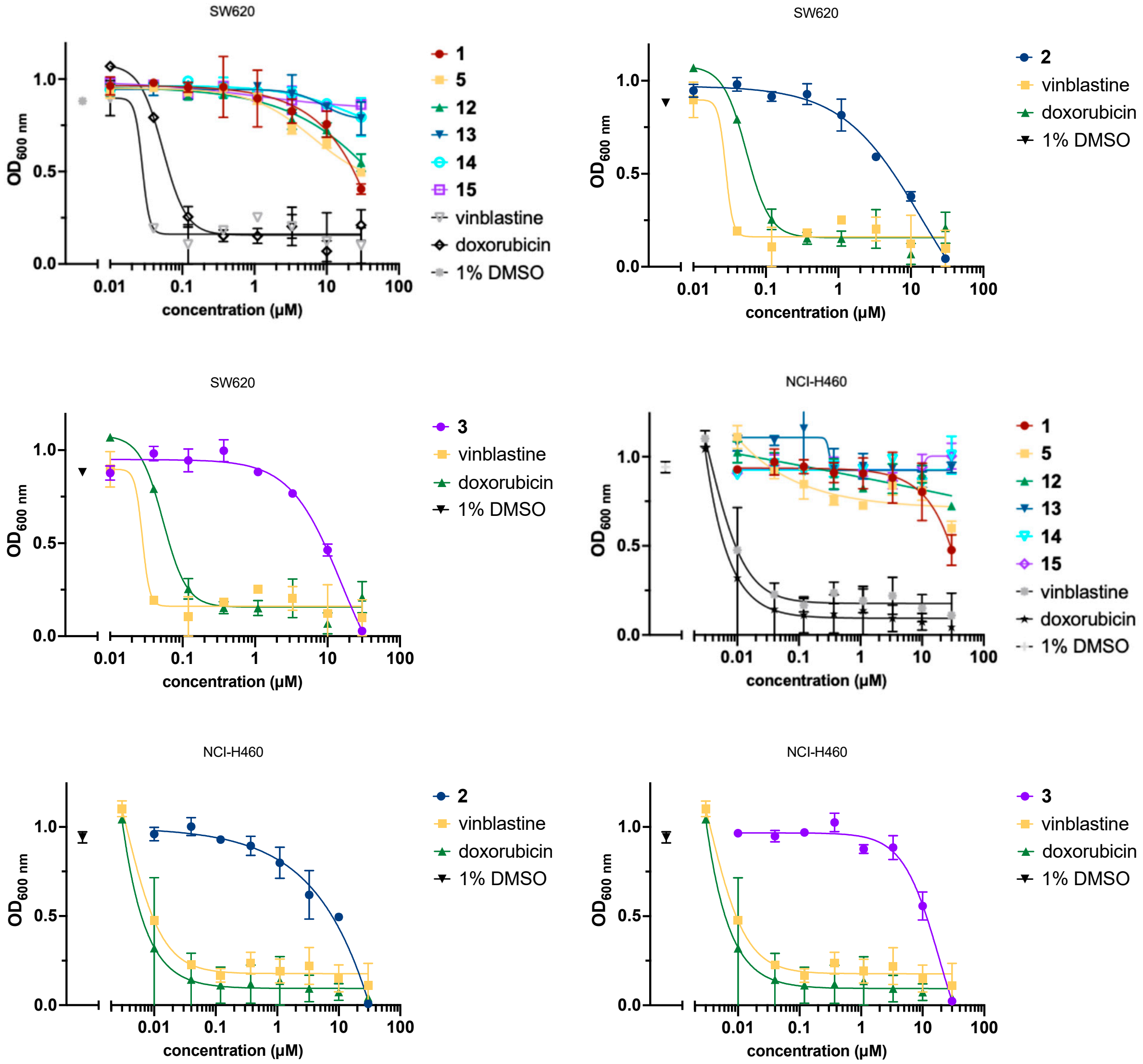

Figure S56. Cytotoxicity of **1–3**, **5** and **12-15**.

Table S14. Cytotoxicity of **1–3**, **5** and **12-15** (IC_{50} μM).

compound	colorectal carcinoma SW620	lung carcinoma NCI-H460
goondoxazole A (**1**)	>30	>30
goondoxazole B (**2**)	10.5	12.2
goondoxazole C (**3**)	7.3	8.7
calcimycin (**5**)	>30	>30
A-33853 (**12**)	>30	>30
UK-1 (**13**)	>30	>30
nataxazole (**14**)	>30	>30
5- hydroxynataxazole (**15**)	>30	>30
vinblastine*	0.03	0.01
doxorubicin*	0.06	0.007

* - positive control.
